# Targeting CD28 on T lineage malignancies with chimeric antigen receptor T cells

**DOI:** 10.1038/s41467-026-78312-3

**Published:** 2026-10-08

**Authors:** Semjon Willier, Julian Färber, Theodora Ispyrlidou, Jonas Wilhelm, Eileen Mayer, Dana Stenger, Jannika Seiferling, Annika E. Brien, Sophia Nikolaides, Paulina Ferrada-Ernst, Jens Dominik Maile, Binje Vick, Andreas Carr, Franziska Blaeschke, Dirk H. Busch, Peter Spieler, Thomas Nerreter, Ramin Lotfi, Martina Rudelius, Theresa Kaeuferle, Christoph Klein, Gabriele Escherich, Irmela Jeremias, Ulrike Burk, Tobias Feuchtinger

**Affiliations:** 1 Department of Pediatrics, Dr. von Hauner Children’s Hospital, LMU University Hospital, LMU Medizin, LMU Munich, Munich, Germany; 2 German Cancer Consortium (DKTK), Partner Site Freiburg, a partnership between DKFZ and University Medical Center Freiburg, Freiburg, Germany; 3 Department of Pediatric Hematology, Oncology and Stem Cell Transplantation, Children’s Hospital, Medical Center and Faculty of Medicine, University of Freiburg, Freiburg, Germany; 4 Research Unit Apoptosis in Hematopoietic Stem Cells, Helmholtz Munich, German Research Center for Environmental Health, Munich, Germany; 5 German Cancer Consortium (DKTK), Partner Site Munich, a partnership between DKFZ and University Hospital LMU Munich, Munich, Germany; 6 Institute for Medical Microbiology, Immunology and Hygiene, TUM School of Medicine and Health, Technical University of Munich (TUM), Munich, Germany; 7 German Cancer Research Center (DKFZ), Heidelberg, Germany; 8 Hopp Children’s Cancer Center Heidelberg (KiTZ), Heidelberg, Germany; 9 Department of Pediatric Hematology and Oncology, Heidelberg University Hospital, Heidelberg, Germany; 10 German Center for Infection Research (DZIF), Partner Site Munich, Munich, Germany; 11 Chair for Cellular Immunotherapy, Department of Medicine II, University Hospital Würzburg, Würzburg, Germany; 12 Institute for Clinical Transfusion Medicine and Immunogenetics Ulm, Ulm, Germany; 13 Institute of Pathology, Ludwig-Maximilians-University of Munich, Munich, Germany; 14 German Center for Child and Adolescent Health (DZKJ), Partner Site Munich, Munich, Germany; 15 Department of Pediatric Hematology and Oncology, University Medical Center Hamburg-Eppendorf (UKE), Hamburg, Germany

**Keywords:** Acute lymphocytic leukaemia, Cancer immunotherapy, Paediatric cancer, T cells

## Abstract

Currently, no immunotherapy is approved for T cell acute lymphoblastic leukemia (T‑ALL). High initial response rates to CD7‑directed chimeric antigen receptor (CAR) T cells are limited by profound T cell aplasia and CD7‑negative immune escape, underscoring the need for alternative targets. Here, we show that CD28 is overexpressed on T‑ALL blasts from children and adolescents compared with lymphoid progenitors from healthy donors and is uniformly upregulated in nodal T-follicular helper cell lymphomas (nTFHL-AI, nTFHL-F). Using CRISPR/Cas9-mediated CD28 knockout to prevent fratricide, we generate highly functional anti‑CD28 CAR T cells with selective cytotoxicity against CD28⁺ T‑ALL. Anti‑CD28 CAR T cells match anti‑CD7 CAR T cells in vitro and in vivo, but cause markedly less lymphodepletion, largely sparing CD8 T cells and NK cells while preferentially depleting CD4 T cells, particularly TH2 and TH17 subsets. These findings establish CD28 as an actionable target for CAR T cell therapy of T cell malignancies.

## Abstract (web summary)

CAR-T cell therapy is currently being evaluated in patients with T-cell acute lymphoblastic leukaemia (T-ALL), however target antigens are limited. Here the authors report that CD28 is highly expressed on primary T-ALL and describe the anti T-ALL activity of anti-CD28 CAR T cells.

## Introduction

Acute lymphoblastic leukemia (ALL) is the most common malignancy in children and adolescents. About 15% of pediatric ALL originate from T cell precursors (T-ALL), while B cell precursor ALL (B-ALL) accounts for about 85%^1^. Improvement of therapy outcomes for patients with T-ALL over the last decades is mainly due to treatment intensification and improved risk stratification^2^. Nevertheless, patients with T-ALL have an inferior outcome compared to patients with B-ALL despite recent advances in the understanding of T-ALL biology^3,4^. Immunotherapy has emerged as a fourth pillar of cancer therapy, with checkpoint blockade and chimeric antigen receptor (CAR) T cells resulting in long-term remissions in patients who previously had no remaining treatment option^5^.

CAR T cells are currently being evaluated for T-ALL in several clinical trials that take advantage of targeting CD7 (search on clinicaltrials.gov: *n* = 15 recruiting trials as of February 2026), CD5 (*n* = 8), or CD1a (*n* = 1). However, while high initial response rates are observed, e.g., after anti-CD7 CAR T cell therapy^6^, relapse rates are high and comprise both CD7-negative and CD7-positive T-ALL^7^. CD7 is expressed early in lymphopoiesis, leading to severe elimination of healthy T cells and precursors through anti-CD7 CAR T cell therapy. As clinical experience with CAR T cells directed against alternative T-ALL antigens is limited^8^, there remains an unmet need for efficient CAR T cell therapy in T-ALL. T-ALL heterogeneity, limited functional relevance of target antigens for T-ALL biology, and shared antigen expression by physiologic T cells and T-ALL blasts are main obstacles on the path to effective CAR T cell therapy in T-ALL.

CD28 has been identified as the first T cell costimulatory molecule more than 40 years ago, and its tremendous functional relevance for expansion and proliferation of T cells has been shown along the way^9^. CD28 provides a distinct costimulatory signal that synergizes with the TCR to enhance lymphokine production and render T cells less susceptible to immunosuppressive agents^10^. This is facilitated by CD28 signaling through PI3K and Akt reprogramming of T cell glucose metabolism to enable sustained T cell proliferation^11^. However, subsequent to CD28 activation, PI3K-independent signaling via VAV and SLP-76 can occur and links CD28 to adapter complexes and cytoskeletal remodeling in T cell activation^12^. Moreover, efficient activation of NF-κB, NFAT and AP-1 was shown to require integration of TCR and CD28 signals via PKCθ as a key effector of CD28-dependent transcriptional control of IL-2 and other cytokines^13^. Finally, CD28 activation can increase IL-2 by stabilizing IL-2 mRNA^14^.

CD28 is expressed on the majority of mature T cells. Surprisingly, families with inborn errors of CD28 have shown only mild symptoms with increased susceptibility to specific viral infections^15^. Additionally, the success of immune checkpoint inhibition by blockade of CTLA-4, the high-affinity CD80/CD86 ligand antagonizing CD28 function in T cells, has underscored the potential of CD28 co-stimulation in physiologic T cells^16^. However, while CD28 overexpression on multiple myeloma has been observed decades ago^17^, information on CD28 in T cell malignancies remains scarce.

Noteworthy, CD28 gain-of-function mutations have been observed in adult T cell leukemia/lymphoma (ATLL), and CD28 expression has been reported as an adverse risk factor in ATLL^18^. In line with this finding, CD28 ligation was reported to lead to CD25 expression and a reduction of CD95-induced apoptosis in AIDS‑associated non‑Hodgkin lymphoma^19^. Triggered by this data and the observation of activating CD28 mutations in some T cell lymphomas^20^, we set out to investigate surface expression and functionality of co-stimulatory molecules in pediatric T-ALL as targets for CAR T cell immunotherapy.

Here, we show that CD28 is overexpressed on pediatric and adolescent T‑ALL blasts and uniformly upregulated in defined T cell lymphoma subtypes. Anti‑CD28 CAR T cells mediate potent, selective antileukemic activity comparable to anti‑CD7 CAR T cells in vitro and in vivo, while causing substantially less lymphodepletion and largely preserving CD8 T cells and NK cells. These findings establish CD28 as a promising target for a potentially less immunosuppressive CAR T cell approach to T cell malignancies.

## Results

### CD28 is overexpressed on primary pediatric T-ALL

**Fig. 1:**
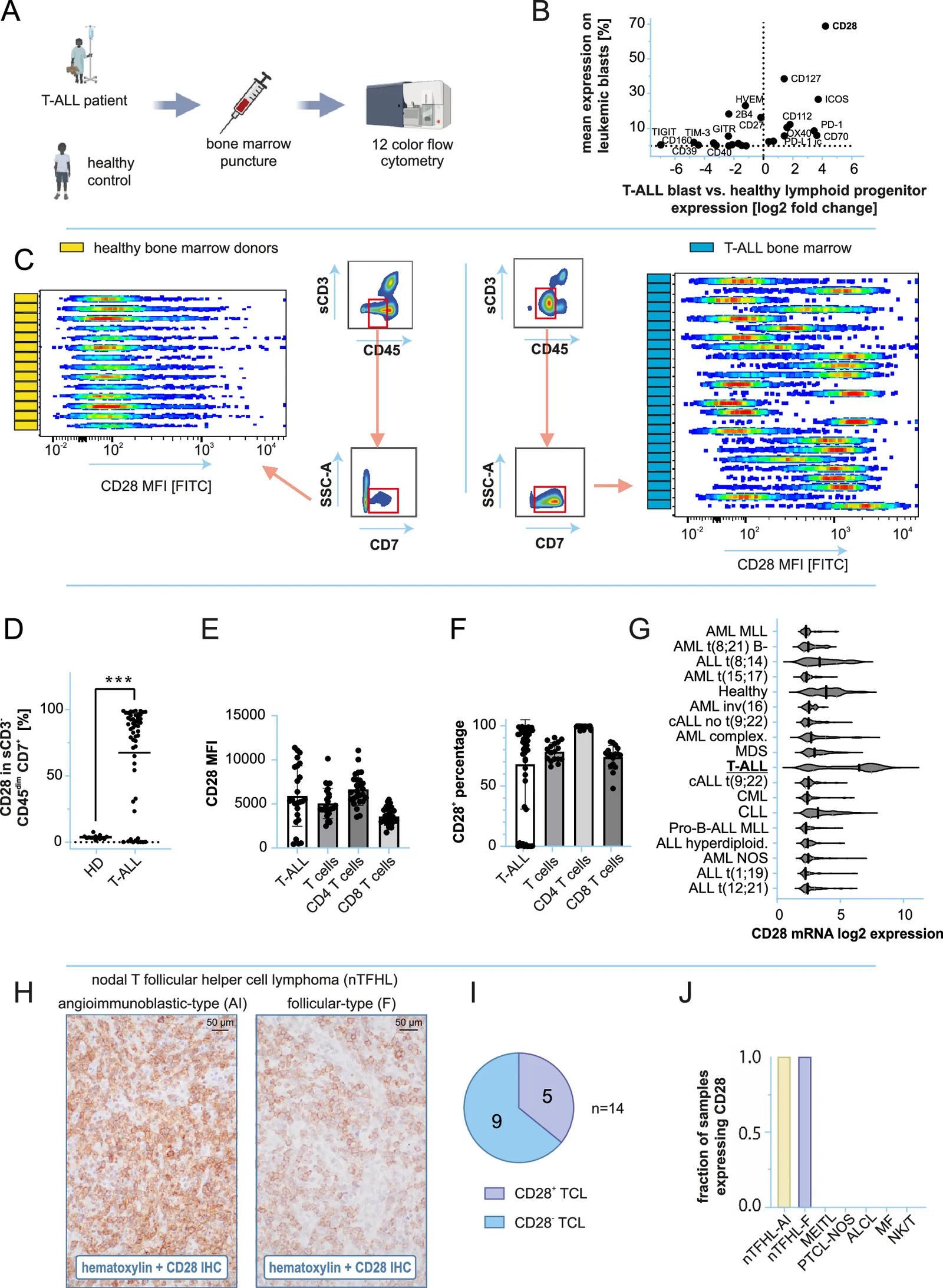
CD28 is overexpressed on pediatric T-ALL and in nodal T follicular helper cell lymphoma. **A** Sample analysis workflow. Created in BioRender. Burk, U. (2026) https://BioRender.com/w0ckthy. **B** Mean surface expression of 25 molecules on T-ALL blasts and differential expression in T-ALL (*n* = 54) vs. healthy lymphoid progenitors (*n* = 14). CD28 shows a mean expression on T-ALL of 67.5% and overexpression in T-ALL vs. healthy lymphoid progenitors of >23-fold. **C** Gating strategy on physiologic lymphoid progenitors (left) and T-ALL blasts (right) after gating on lymphocytes, single and living cells by gating on CD45^dim^ sCD3^−^ cells and CD7^+^ cells. CD28 expression on lymphoid progenitors of healthy donors (*n* = 14, left) and representative T-ALL samples (*n* = 25, right). **D** CD28 surface expression in healthy lymphoid progenitors (*n* = 14) and T-ALL blasts (*n* = 52; mean 3.7 vs. 67.5%, median 3.4 vs. 85.8%, *p* = 0.00017, two-sided Mann-Whitney test). **E** CD28 expression by flow cytometry median fluorescence intensity (MFI) in T-ALL blasts (mean MFI = 5893), physiologic bone marrow T cells (mean MFI = 5057), and healthy CD4 (mean MFI = 6639) and CD8 (mean MFI = 3545) bone marrow T cells from patients with T-ALL (*n* = 26). Data are presented as mean values ± SD. **F** CD28 expression by percentage in the same populations (T-ALL, *n* = 50; T cells, *n* = 17; CD4 and CD8 T cells, *n* = 17). T-ALL (mean=67.5%, SD 37%) and CD4 T cells (99%, SD 1.4%). **G** Reanalysis of published microarray from the MILE study (GSE13159) confirms CD28 overexpression in T-ALL (*n* = 174) by mRNA profiling of 2113 samples from hematologic malignancies. **H** CD28 expression in nodal T follicular helper cell lymphoma (nTFHL) angioimmunoblastic-type (left panel, representative of three samples) and nTFHL follicular-type (right panel, representative of two samples) on 4%-PFA-fixed samples. **I** After testing a total of 14 T cell lymphomas (TCL), 5 samples were positive for CD28. **J** Of these, 3/3 nTFHL-AI and 2/2 nTFHL-F tested were positive for CD28, while 0/9 of other T cell lymphomas showed CD28 expression (MEITL Monomorphic epitheliotropic intestinal T cell lymphoma, PTLC-NOS Peripheral T Cell Lymphoma, not Otherwise Specified, ALCL Anaplastic large cell lymphoma, MF Mycosis fungoides, NK/T NK/T cell lymphoma). Source data are provided as a Source data file.

In order to identify novel targets for immunotherapy, we compiled a list of 25 potential antigens with known implications in T cell functionality^21^. We analyzed a cohort of 54 pediatric patients with T-ALL at the time of diagnosis and 14 healthy who underwent bone marrow puncture as donors for bone marrow transplantation (Fig. 1A, Table 1). After gating on lymphoid progenitors defined as CD45^dim^/CD3^dim^ and CD7^+^ (Fig. 1C, Supplementary Fig. 1A, B), we analyzed protein surface expression of potential target molecules. While we observed reduced expression of several co-inhibitory surface molecules (TIGIT, CD160 and TIM-3), co-stimulatory molecules such as CD127, ICOS and CD28 showed increased expression in T-ALL blasts when compared to physiologic lymphoid progenitors (Fig. 1B, C, Supplementary Table 2). Indeed, 81.5% of patients expressed CD28 with a mean expression of 67.5% in T-ALL blasts vs. 3.7% in healthy lymphoid progenitors (*p* = 0.0002, Fig. 1D, Supplementary Table 3). CD28 expression in CD28^+^ T-ALL cases was comparable to that observed on physiologic CD4 T cells measured by median fluorescence intensity (MFI, Fig. 1E) and percentage of CD28 expression was slightly lower than in physiologic T cells (Fig. 1F). In analogy, CD7 expression MFI was lower than in physiologic T cells (Supplementary Fig. 1C). The observation of CD28 overexpression in T-ALL was substantiated by analysis of published mRNA expression data, where we observed highest expression of CD28 in T-ALL (Fig. 1G, GSE13159 and Supplementary Fig. 2B)^22^. CD28 expression was independent of patient age and unchanged in primary and relapsed T-ALL samples (Supplementary Fig. 2A, F). While CD28 expression showed a weak inverse correlation with 2B4 expression on T-ALL (Supplementary Fig. 2C, *p* = 0.0023, slope −0.48), we did not observe a clear pattern of co-stimulatory and -inhibitory marker surface expression on T-ALL.

**Table 1:** Characteristics of patients with T-ALL and healthy donors

	T-ALL patients	Healthy BM^a^ donors
Patients, *n*	54	14
Number of samples	58	14
Age at diagnosis, Mean (range)	9.1 years (3.0–17.0)	10.3 years (1.7–19.1)
Gender, *n* (%)		
Male	50 (87)	7 (50)
Female	8 (13)	7 (50)
Treatment status, *n* (%)		
Therapy naive	45 (77.6)	-
Treated	7 (12.1)	-
Unknown status	6 (10.3)	-
Future relapse, *n* (%)	11 (20.4)	-
Death, *n* (%)	12 (22.2)	-
Time to relapse, mean (range)	1.8 (0.3–8.5)	-
Monitoring period, mean (range)	4.6 (0.1–12.1)	-

^a^*BM* bone marrow donor.

In B cells, CD28 is expressed during physiologic maturation towards plasma cells and is involved in multiple myeloma pathogenesis, while it is not expressed in immature B cell malignancies such as B-ALL^17,23^. In line with CD28 overexpression in immature T cell malignancies (e.g., T-ALL), we investigated CD28 expression also in mature T cell malignancies. When we analyzed T cell lymphoma patient samples, we identified two recently defined T cell lymphoma entities (nodal T-follicular helper cell lymphoma angioimmunoblastic-type or nTFHL-AI and follicular-type or nTFHL-F)^15^, which consistently express CD28 in 3/3 and 2/2 samples studied (Fig. 1H), while other T cell lymphomas analyzed did not show CD28 expression (Fig. 1I, J). Thus, CD28 may represent an immunotherapeutic target in pediatric T-ALL, nTFHL-AI, nTFHL-F, and multiple myeloma.

Investigating CD7 and CD28 expression in physiologic T cells in the bone marrow of healthy donors and patients with T-ALL, we found that CD7 was expressed more frequently than CD28 in both healthy donors (91.6 vs. 81.8%) and patients with T-ALL (96.0 vs. 74.8%; Supplementary Fig. 2D, E). Moreover, analysis of published single-cell RNA-seq data indicated that CD28 expression occurs later in T cell development than both CD7 and CD2 (Supplementary Fig. 2H–K)^24^. Both observations could indicate lower on-target off-tumor toxicity when targeting CD28 compared to targeting CD7 in patients with T-ALL.

To assess the functional relevance of CD28 overexpression in T-ALL, we examined its contribution to leukemic-cell proliferation. Although CD86-expressing cells were detectable in T-ALL bone marrow at lower frequencies than in healthy controls (Supplementary Fig. 2G), co-culture of CCRF-CEM cells with CD86-expressing monocyte-derived dendritic cells (moDCs) increased proliferation under low-serum conditions (Supplementary Fig. 3A, D). IL-7 alone similarly enhanced CCRF-CEM proliferation (Supplementary Fig. 3B), whereas CD28 knockout did not affect basal proliferation (Supplementary Fig. 3C). Moreover, blockade of CD80/CD86 using the CTLA4-Fc fusion protein abatacept did not reduce moDC-induced proliferation, indicating that this effect was independent of CD28 ligation (Supplementary Fig. 3E). In contrast, under JAK inhibition with ruxolitinib, CD28 ligation by monoclonal antibodies increased proliferation of both CCRF-CEM and Jurkat cells in an abatacept-sensitive manner (Supplementary Fig. 3F, G). These data suggest that JAK blockade unmasks a latent, JAK-independent CD28-mediated pro-survival program, potentially involving PI3K/AKT signaling. CD28 was internalized following soluble antibody exposure, more prominently in Jurkat than in CCRF-CEM cells (Supplementary Fig. 14). Together, these findings indicate that CD28 is dispensable for basal T-ALL proliferation but can provide context-dependent survival signals, supporting its evaluation as a CAR T-cell target.

### Anti-CD28 CAR T cells eliminate T-ALL in vitro, and CD28 knockout T cells retain effector functionality while avoiding fratricide

**Fig. 2:**
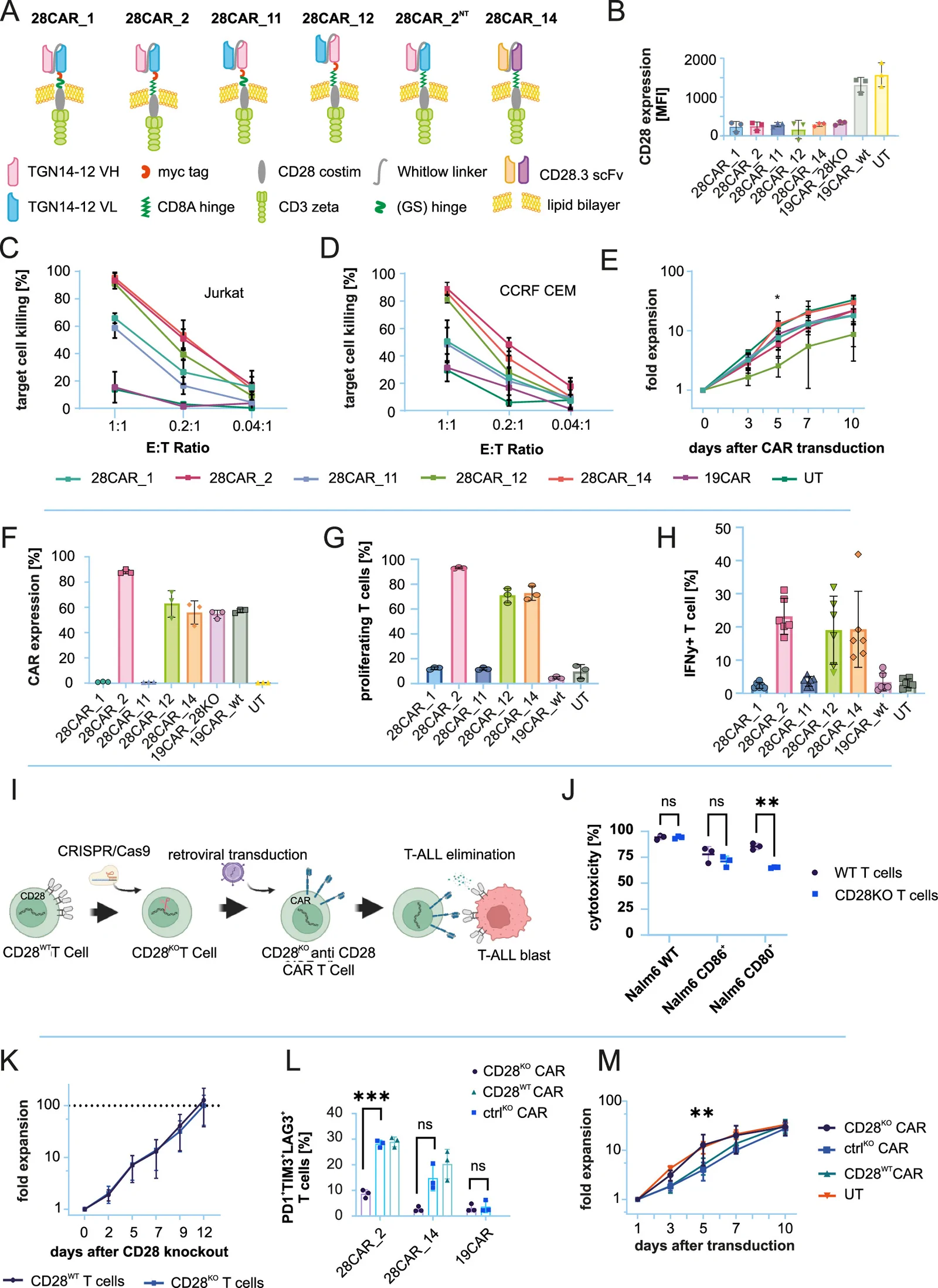
Identification of two highly functional anti-CD28 CAR molecules, 28CAR_2 and 28CAR_14. **A** Functional and structural features of TGN1412-based CARs and 28CAR_14. TGN1412 VH/VL: variable heavy/light domain. **B** Five anti-CD28 CARs reduced CD28 surface expression on transduced WT T cells (mean MFI 168–300) compared with controls (19CAR: 1330; UT: 1591) and CD28 KO control (19CAR_28KO: 335). **C**, **D** Cytotoxicity of anti-CD28 CAR T cells against CCRF-CEM or Jurkat cells at E:T ratios of 1:1, 0.2:1, and 0.04:1, quantified after 48 h. **E** Anti-CD28 CAR T cell expansion. At day 5, 28CAR_12 expanded only 2.6-fold versus 5.9-12.8-fold for all other constructs (*p* = 0.0422, Kruskal–Wallis test). **F** Detection of the myc tag. Anti-CD28 CARs with a short (G4S)₃ spacer (28CAR_1, 28CAR_11) were not surface-detectable (1% each) despite downregulating CD28, whereas constructs with the longer CD8α-derived hinge were detected (56–88%). **G** Proliferation of anti-CD28 CAR T cells after 72 h co-culture with CCRF-CEM cells (CTV dilution). 28CAR_2, 28CAR_12 and 28CAR_14 T cells proliferated more strongly (93%, 71%, 82%) than all other constructs. **H** Interferon gamma (IFNγ) release (intracellular cytokine staining) after 24 h co-culture with CCRF-CEM. Only 28CAR_2, 28CAR_12 and 28CAR_14 responded (19.0–23.2% versus 2.4–3.6%). **I** Schematic of anti-CD28 CAR T cell generation by CRISPR/Cas9-mediated CD28 knockout and retroviral transduction. Created in BioRender. Burk, U. (2026) https://BioRender.com/p5vkppb. **J** Blinatumomab-redirected (0.5 µg/ml) cytotoxicity against CD19^+^ Nalm6 cells. Onlyagainst CD80 overexpressing Nalm6 cells, CD28 KO T cells showed reduced cytotoxicity compared to WT T cells (85.5 vs. 65.2%, *p* = 0.0077, paired, two-sided *t*-test, no multiple comparison correction). **K** T cell expansion after CD28 KO on day 0 was unchanged compared with WT T cells. **L** Concurrent LAG-3^+^PD-1^+^TIM-3^+^ frequency as a correlate of fratricide 7 days after T cell activation. CD28 KO reduced fratricide in 28CAR_2 (*p* = 0.0009), with a trend in 28CAR_14 (*p* = 0.059) and no change in 19CAR T cells (*p* = 0.38, paired two-tailed *t* tests). **M** Transduction without initial CD28 KO reduced 28CAR_14 T cell expansion consistent with fratricide; CD28 knockout restored expansion to UT levels (day 5, *p* = 0.0087, Kruskal–Wallis test). **B**–**H**, **J**–**M** Three independent T cell donors; data are presented as mean values ± SD. Source data are provided as a Source data file.

We designed 20 chimeric antigen receptor (CAR) molecules against CD28 (Fig. 2A, Table 2) and identified five functional CAR molecules that led to reduced surface expression of CD28 within the transduced T cell population (Fig. 2B). Five CAR molecules conferred CD28 specific cytotoxicity against two T-ALL cell lines (Fig. 2C, D, Supplementary Fig. 4F). However, 28CAR_12 conferred markedly reduced expansion in transduced T cells after CD28 KO (Fig. 2E). Three anti-CD28 CAR molecules were detectable on the cell surface by staining of a myc tag on transduced T cells (Fig. 2F). 28CAR_1 and 28CAR_11 CAR T cells yielded reduced cytotoxicity, interferon gamma (IFNγ) and tumor necrosis factor alpha (TNFα) release, and proliferation after target cell contact (Fig. 2G, H, Supplementary Fig. 4A, B). Interestingly, CAR molecules harboring a short hinge domain (28CAR_1, 28CAR_11) could scarcely be detected by myc tag staining and showed lower recombinant CD28 protein binding compared to EGFRt expression coded on the same vector after a F2A sequence (Supplementary Fig. 4C). Consequently, 28CAR_2 and 28CAR_14 were identified as lead anti-CD28 CAR constructs. Asking whether anti-CD28 CAR T cell killing was IFNγ-dependent^25^ we performed assays using IFNγ blockade and observed, as expected, CAR T cell killing independent of IFNγ (Supplementary Fig. 12A). By analogy, inhibition of TNFα did not impact T-ALL killing by anti-CD28 CAR T cells (Supplementary Fig. 12B). At the same time, we observed high Granzyme B expression in anti-CD28 CAR T cells by intracellular cytokine staining (Supplementary Fig. 16A–D). Perforin and granzymes A and B were markedly increased in co-culture supernatants after anti-CD28 CAR T cell exposure to T-ALL target cells, indicating that the perforin–granzyme pathway mediates their cytotoxicity against CD28^+^ target cells (Supplementary Fig. 16E).

**Table 2:** Anti-CD28 CAR design, based on five single-chain variable fragments

28CAR ID	scFv	scFv orientation	Hinge
28CAR_1	TGN1412	Light – heavy	Short
28CAR_2	TGN1412	Light – heavy	Long
28CAR_3	CD28.3	Light – heavy	Short
28CAR_4	CD28.3	Light – heavy	Long
28CAR_5	Cl1B4	Light – heavy	Short
28CAR_6	Cl1B4	Light – heavy	Long
28CAR_7	cl83	Light – heavy	Short
28CAR_8	cl83	Light – heavy	Long
28CAR_9	CD28#5	Light – heavy	Short
28CAR_10	CD28#5	Light – heavy	Long
28CAR_11	TGN1412	Heavy – light	Short
28CAR_12	TGN1412	Heavy – light	Long
28CAR_13	CD28.3	Heavy – light	Short
28CAR_14	CD28.3	Heavy – light	Long
28CAR_15	Cl1B4	Heavy – light	Short
28CAR_16	Cl1B4	Heavy – light	Long
28CAR_17	cl83	Heavy – light	Short
28CAR_18	cl83	Heavy – light	Long
28CAR_19	CD28#5	Heavy – light	Short
28CAR_20	CD28#5	Heavy – light	Long

As cytotoxic lymphocyte killing relies on the LFA-1–ICAM-1/ICAM-2 adhesion axis, we asked whether LFA-1 has a role in anti-CD28 CAR T cell killing. To that end, we performed co‑cultures of primary human CAR T cells with repetitive addition of GFP‑labeled Jurkat cells in the presence or absence of an anti‑LFA‑1 blocking antibody (Supplementary Fig. 17). LFA‑1 blockade consistently increased the fraction of surviving Jurkat cells in two independent donors for anti‑CD28 CAR T cells using 28CAR_2 and 28CAR_14 and for anti‑CD7 CAR T cells. While killing after the first T-ALL challenge was attenuated in one donor, killing after the third T-ALL challenge was reduced in the other. No difference between antibody conditions was observed for poorer-performing 28CAR_12. Together, these data indicate that LFA‑1 blockade has a modest impact on repetitive T‑ALL killing by anti-CD28 CAR T cells, while overall cytotoxic capacity is maintained. This is in line with recent findings that LFA-1 primarily enhances cytotoxic lymphocyte synapse stability and cytotoxic efficiency rather than acting as an absolute requirement for target cell killing^26^.

Since CD28 is widely expressed on physiologic T cells and involved in essential T cell functions, we set out to determine the functionality of CD28 knockout (KO) T cells. To that end, we performed Clustered Regularly Interspaced Short Palindromic Repeats (CRISPR)/Cas9 mediated KO of CD28 in T cells and observed a mean KO rate of 94.0% (Fig. 2I, Supplementary Fig. 4D). To test CD28 KO T cell cytotoxic functionality, CD28 KO T cells were co-cultured with either WT Nalm6 cells, CD86 overexpressing Nalm6 cells or CD80 overexpressing Nalm6 cells in the presence of CD3/CD19 bispecific T cell engager blinatumomab. As CD80 and CD86 are natural ligands of CD28, their presence should lead to a functional advantage of CD28-expressing T cells in our setup. We observed unaltered cytotoxicity against WT and low-affinity CD28 ligand CD86-overexpressing Nalm6, while CD28 KO T cell cytotoxicity was significantly reduced against high-affinity CD28 ligand CD80-overexpressing Nalm6 cells (Fig. 2J). CD28 KO T cells expanded comparably to WT T cells until day 12 after electroporation, and CD28 KO was stable over time (Fig. 2K, Supplementary Fig. 4E). These results confirm that in the presence of a ligand, CD28 expression becomes functionally relevant, whereas in the absence of a ligand, CD28 knock-out T cells retain functionality. CD28 KO in T cells before transduction of anti-CD28 CAR molecules resulted in a reduced frequency of LAG-3⁺TIM-3⁺PD-1⁺ triple-positive T cells both in 28CAR_2 and 28CAR_14 transduced T cells (Fig. 2L). In line with this, the profile of T cell expansion after CAR transduction showed signs of fratricide in CD28 WT T cells, with significantly slowed T cell expansion without CD28 KO prior to 28CAR_14 transduction (Fig. 2M). Consequently, CD28 KO was included in the subsequent anti-CD28 CAR T cell generation.

In order to ascertain the safety of anti-CD28 CAR T cells with a potentially superagonistic scFv (scFv of TGN1412)^27,28^, we co-cultured CTV-labeled WT T cells with anti-CD28 CAR T cells. After 24 and 48 h of co-culture, we observed neither altered activation nor altered proliferation of target WT T cells (Supplementary Fig. 5A–E).

### Two lead anti-CD28 CARs match anti-CD7 CAR activity against T-ALL

**Fig. 3:**
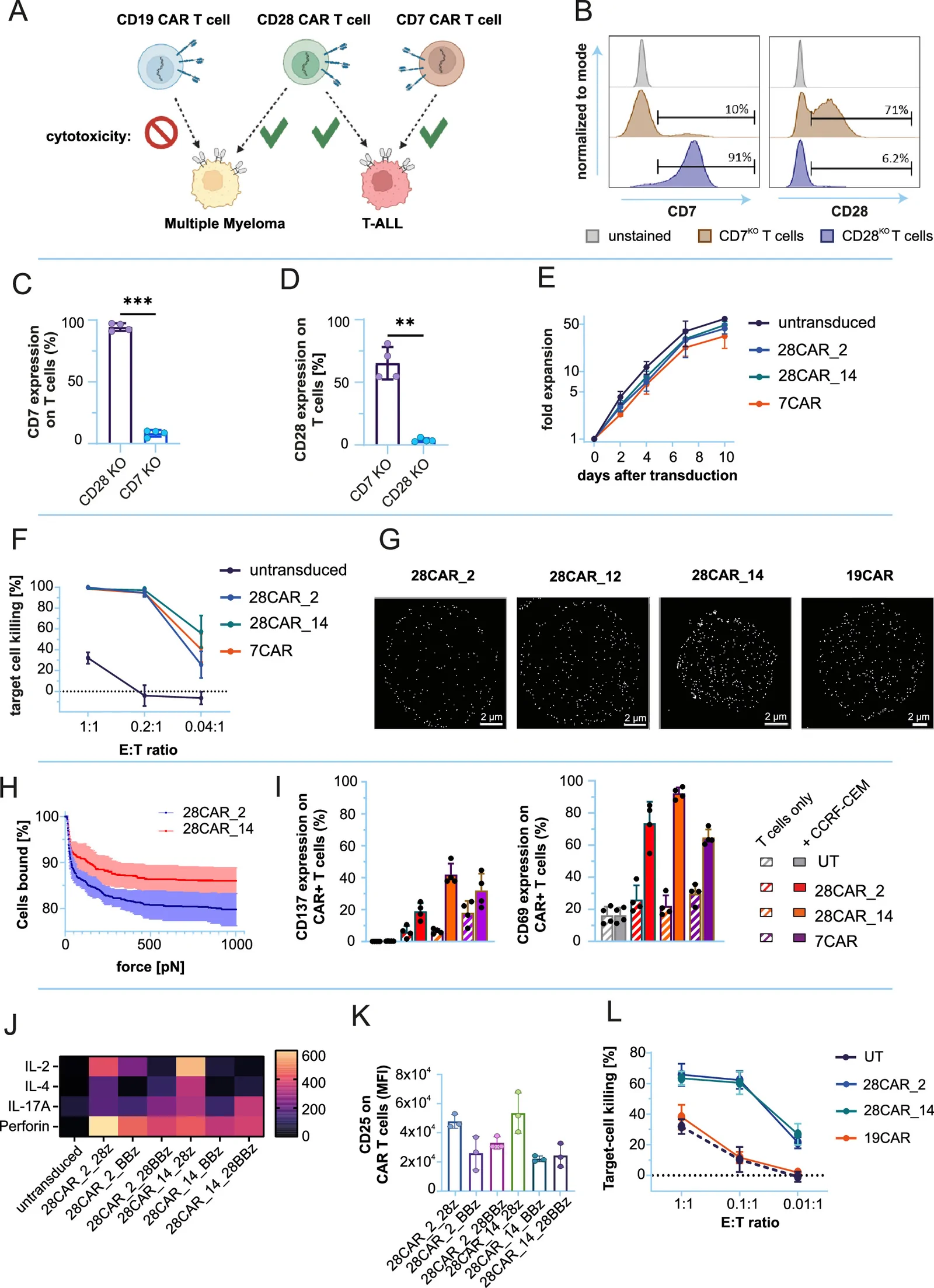
Two lead anti-CD28 CARs show anti-T-ALL activity comparable to anti-CD7 CARs and eliminate multiple myeloma cell lines in vitro. **A** Graphical summary of anti-CD28 CAR T cell activity against T-ALL and MM cell lines compared with anti-CD7 and anti-CD19 CAR T cells. Created in BioRender. Burk, U. (2026) https://BioRender.com/6jpvx98. **B** Representative flow cytometry plots showing CD7 and CD28 knockout (KO) efficiency following CRISPR/Cas9 editing. **C**, **D** After 14 days of expansion, CD7KO reduced CD7 expression from 94% to 8% (paired, two-tailed *t*-test, *p* < 0.0001, **C**). CD28 expression decreased from 66% to 4% following CD28KO (paired, two-tailed *t*-test, *p* = 0.0022, **D**). **E** Expansion of 28CAR_2, 28CAR_14 and 7CAR T cells following CD7 or CD28 KO. **F** Cytotoxicity against CCRF-CEM cells at the indicated E:T ratios. **G** dSTORM imaging of 28CAR_2, 28CAR_14 and anti-CD19 CAR molecules on representative T cells (297 cells analyzed, cf. Supplementary Fig. 4G). **H** Cellular avidity for Jurkat cells was measured by z-Movi. At 1000 pN, 28CAR_14-transduced CD28KO Jurkat cells showed greater avidity than 28CAR_2-transduced CD28KO Jurkat cells (86.6% versus 82.2%; *p* = 0.029). Shading denotes mean ± SD of four paired replicates performed on the same chip. **I** Following 24 h co-culture with GFP-expressing CCRF-CEM cells or no targets, CD69 expression was comparable among CAR T cells, whereas CD137 expression was lower in 28CAR_2 (18.8%) than in 28CAR_14 (41.5%) and 7CAR T cells (30.8%). **J** Cytokines in supernatants collected after 48 h co-culture with CCRF-CEM cells (E:T, 1:2). IL-2 was highest in CD28-costimulated anti-CD28 CAR T cells. IL-4 was undetectable in 4-1BB-costimulated anti-CD28 CAR T cells. **K** CD25 expression, measured as MFI, was higher in anti-CD28 CAR T cells with a CD28 costimulatory domain after 48 h co-culture with CCRF-CEM cells. **L** Cytotoxicity against MM.1S cells after 48 h. 28CAR_2 and 28CAR_14 eliminated MM.1S cells at an E:T ratio of 0.01:1, whereas anti-CD19 CAR T cells did not exceed background cytotoxicity of UT cells. Unless otherwise stated, data represent mean ± s.d. from four independent T cell donors (**C**–**E**, **I**) or three independent donors (**F**, **J**–**L**). Source data are provided as a Source data file.

As current clinical trials using anti-CD7 CAR T cells have yielded promising results, we used them as a benchmark control for anti-CD28 CAR T cells (Fig. 3A). First, we performed CRISPR/Cas9 mediated KO of CD28 or CD7 in CD3/CD28 activated primary T cells and observed KO rates of ~ 85% (CD7) and >90% (CD28, Fig. 3B–D). Expansion of 28CAR_2 and 28CAR_14 T cells was similar to anti-CD7 CAR T cell expansion, and the same was true for cytotoxicity against T-ALL cell line CCRF-CEM (Fig. 3E, F). Cellular composition analysis yielded comparable results for 28CAR_2, 28CAR_14 and 7CAR T cells, while central memory T cells were slightly reduced in 28CAR_2 T cells (Supplementary Fig. 7A, B). 28CAR_2 and 28CAR_14 surface expression patterns resembled anti-CD19 CAR expression patterns, albeit with a generally lower CAR surface density of anti-CD28 CAR molecules as measured by direct stochastic optical reconstruction microscopy (dSTORM, Fig. 3G, Supplementary Fig. 4G)^29^. Cellular avidity as measured on the z-Movi device showed superior avidity of 28CAR_14 transduced Jurkat cells when compared with 28CAR_2 transduced Jurkat cells (Fig. 3H, Supplementary Fig. 4H). Also, activation of anti-CD28 CAR T cells, as measured by CD137 and CD69 surface expression, reached similar levels as anti-CD7 CAR T cells, with 28CAR_14 T cells exceeding 28CAR_2 T cell activation (Fig. 3I).

Next, we examined the functionality of anti-CD28 CAR T cells with 41BB and 28BB third-generation co-stimulatory domains. While cytotoxicity was maintained in anti-CD28 CAR T cells harboring alternative co-stimulation, CD28 co-stimulation conferred the highest short-term cytotoxicity (Supplementary Fig. 6A, B) and IL-2/Perforin secretion (Fig. 3J). CD25 expression was highest in CARs containing a CD28 costimulatory domain (Fig. 3K).

Moreover, while incubation of CCRF-CEM cells with TGN1412 antibody (ichorbio, scFv used in 28CAR_2) led to reduced CCRF-CEM proliferation, CD28.2 antibody (eBioscience, closely related to CD28.3, used in 28CAR_14) did not alter CCRF-CEM proliferation (Supplementary Fig. 8A, B). In summary, these data indicate that CD28 costimulatory domains lead to the strongest short-term functionality in both lead molecules 28CAR_2 and 28CAR_14 CAR T cells and that TGN1412-based CAR molecules might exert a T cell-independent effect on T-ALL.

### Anti-CD28 CAR T cells eliminate multiple myeloma cell lines in vitro

Finally, we tested both anti-CD28 CAR T cells for cytotoxicity against two multiple myeloma (MM) cell lines (MM.1S and RPMI 8226, Supplementary Fig. 9). As low CD19 expression on multiple myeloma has been previously reported^30^, we used anti-CD19 CAR T cells as a reference. We observed effective killing of MM cell lines by both 28CAR_2 and 28CAR_14 T cells, while anti-CD19 CAR T cells showed no specific cytotoxicity against MM (Fig. 3L).

### Anti-CD28 CAR T cells confer increased survival and reduced T-ALL growth in a T-ALL xenograft mouse model

**Fig. 4:**
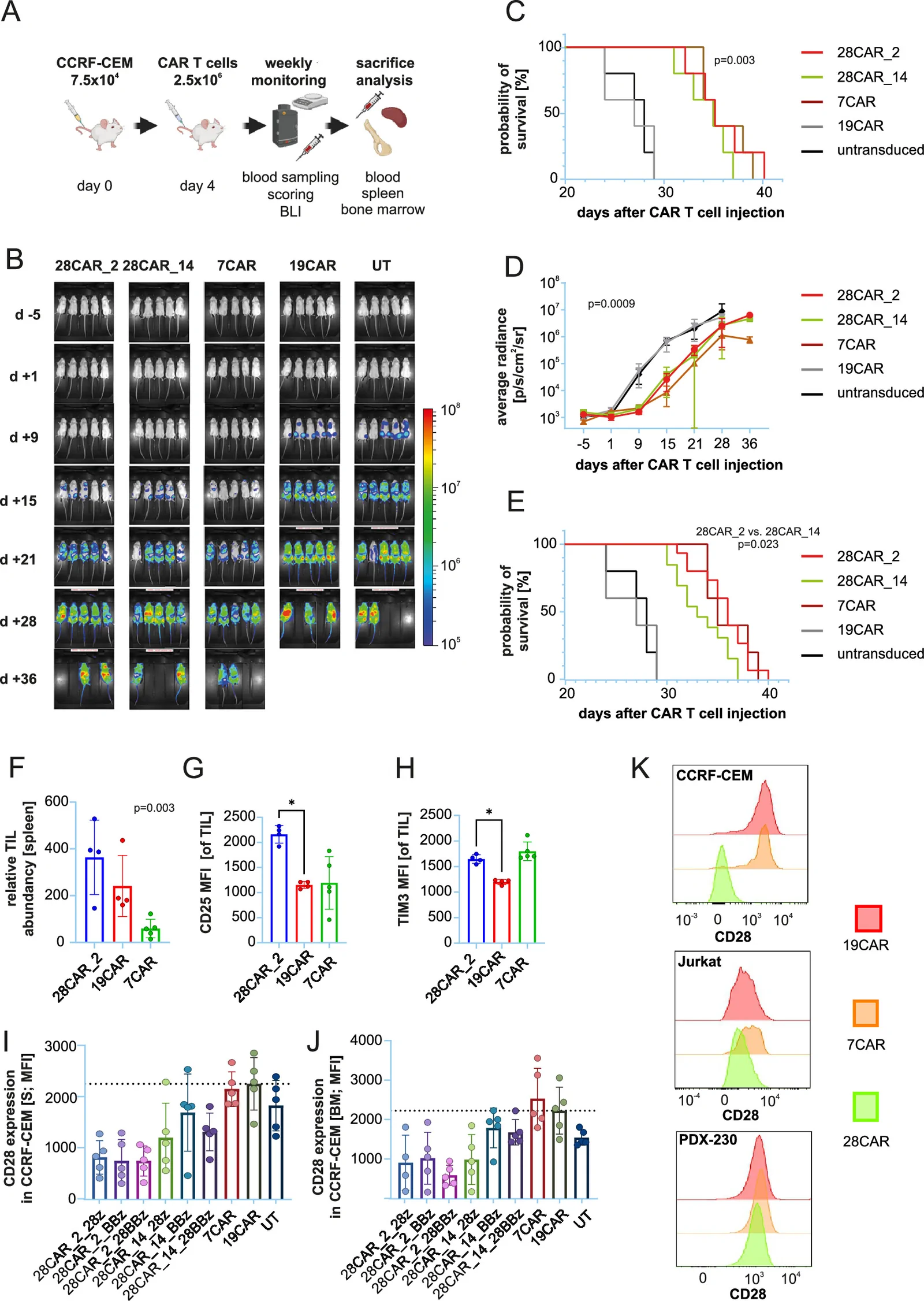
Anti-CD28 CAR T cells eliminate T-ALL in vivo comparably to anti-CD7 CAR T cells. **A** Schematic of the in vivo T-ALL experiment. Created in BioRender. Burk, U. (2026) https://BioRender.com/i1aw0fk. **B** Representative bioluminescence imaging (BLI) of mice treated with the indicated CAR or untransduced (UT) T cells from day −5 to day 36 after CAR T cell injection. **C** Mice treated with 28CAR_2, 28CAR_14, or 7CAR T cells harboring CD28 costimulatory domains showed prolonged survival (median, 35 d each) compared with UT (28 d) or 19CAR T cells (27 d; *n* = 5 per group, log-rank test for trend, *p* = 0.0003). **D** BLI quantification showed delayed T-ALL growth in mice treated with anti-CD7 or anti-CD28 CAR T cells (two-way ANOVA, *p* = 0.0009). **E** Survival of mice treated with pooled 28CAR_2 or 28CAR_14 T cells across CD28, 4-1BB and 28BB costimulatory domains (*n* = 15 mice per CAR; *n* = 5 mice each for 7CAR, 19CAR and UT). Median survival was 36 d for 28CAR_2 and 33 d for 28CAR_14 (log-rank Mantel–Cox test, *p* = 0.023). **F**–**H** Splenic tumor-infiltrating T cells were quantified and phenotyped by flow cytometry 3 d after CAR T cell injection (*n* = 5 mice per group). 28CAR_2-treated mice had more GFP^−^CD45^hi^CD3^+^ events (363) than 19CAR-treated (241) or 7CAR-treated mice (59; Kruskal-Wallis test: *p* = 0.003). Mean TIM-3 MFI was 1648, 1199, and 1799, respectively (Kruskal–Wallis test, *p* = 0.0009; 28CAR_2 versus 19CAR, two-sided Mann–Whitney *U* test, *p* = 0.029). Mean CD25 MFI was 2162, 1153, and 1193, respectively (Kruskal–Wallis test, *p* = 0.011; 28CAR_2 versus 19CAR, two-sided Mann–Whitney *U* test, *p* = 0.029). CD28 surface expression on CCRF-CEM cells recovered from spleen (**I**) or bone marrow (**J**) was reduced after treatment with CD28-targeting CAR T cells (*n* = 5 mice per group, 28CAR_2_28z spleen: 4 mice). **K** Independent in vivo experiments using CCRF-CEM, Jurkat, or ALL-230 patient-derived xenograft (PDX) T-ALL cells. Representative plots show reduced CD28 expression on relapsed CCRF-CEM cells, but unchanged CD28 expression on relapsed Jurkat and ALL-230 cells recovered from bone marrow. Data are mean ± s.d.; *n* = 5 mice per group unless otherwise indicated. Source data are provided as a Source data file.

We went on to validate anti-CD28 CAR T cell functionality against T-ALL in a xenograft mouse model using CCRF-CEM in NXG mice (Fig. 4A). After set-up of a CCRF-CEM T-ALL xenograft mouse model (see methods), we included both 28CAR_2 and 28CAR_14 CAR T cells harboring either CD28, 41BB, or 28BB costimulatory domains. We observed significantly reduced leukemia growth as measured by bioluminescence imaging with both 28CAR_2 and 28CAR_14 constructs (Fig. 4B, D). This inhibition of leukemia growth was independent of the costimulatory domain contained in the CAR construct (Supplementary Fig. 10J). Survival was prolonged by administration of anti-CD28 CAR T cells harboring a CD28 costimulatory domain to the same extent as it was by anti-CD7 CAR T cells (Fig. 4C). Mice treated with 28CAR_2 T cells harboring either a 41BB, CD28 or 28BB costimulatory domain had significantly increased survival when compared to 28CAR_14 treated mice (*n* = 15 per group, median survival 36 vs 33 days, *p *= 0.023, Fig. 4E). Moreover, 28CAR_2 T cells showed significantly increased persistence 11 days after injection compared to both 28CAR_14 and 7CAR T cells (Supplementary Fig. 10E). Accordingly, subsequent experiments focused on 28CAR_2 T cells. At the same time, the costimulatory domain of the CAR molecule had no impact on mouse survival (Supplementary Fig. 10J).

In order to define the phenotype of best-performing anti-CD28 CAR T cells (28CAR_2) tumor-infiltrating lymphocytes (TILs, Supplementary Fig. 15A), we repeated the above-mentioned experiment and sacrificed mice on day 3 after CAR T cell injection. Here, we observed a significant increase in 28CAR_2 TILs within the spleen when compared to 7CAR T cells, as well as increased CD25 MFI in 28CAR_2 T cells and increased TIM3 expression in both 28CAR_2 T cells and 7CAR T cells when compared to 19CAR T cell control-treated mice, while CD127 expression was reduced in 7CAR T cells (Fig. 4F–H, Supplementary Fig. 10F, G).

Of note, expression of CD28 was reduced on CCRF-CEM cells in both the bone marrow and spleen of sacrificed mice treated with either anti-CD28 CAR T cells (Fig. 4I, J, Supplementary Fig. 15B). This was not observed for CD7 surface expression on CCRF-CEM cells in anti-CD7 CAR-treated mice (Supplementary Fig. 10H, I). In order to elucidate whether reduced CD28 expression on T-ALL relapse could generally reduce the applicability of anti-CD28 CAR T cells, we employed two alternative T-ALL mouse models based on a cell line (Jurkat) and a patient-derived xenograft (ALL-230). 28CAR_2 T cells led to prolonged survival in both models, while 7CAR T cells showed efficacy only in the Jurkat model, where it was more efficient (Supplementary Fig. 11, left and middle panels). Importantly, while CD28 expression on relapsed or persisting CCRF-CEM cells showed a marked decrease again, CD28 expression on relapsed or persisting Jurkat and ALL-230 cells showed constant CD28 surface expression (Fig. 4K and Supplementary Fig. 11, right panel). Interestingly, CD28 expression was highest in CCRF-CEM when compared to Jurkat and ALL-230 (Supplementary Fig. 11D). Consequently, 28CAR_2 T cells are a candidate therapy for treatment of patients with T-ALL with CD28^+^ disease, and immune escape is not a general impediment for this therapy.

### Anti‑CD28 CAR T cells induce milder, CCR4⁺/CCR6⁺‑focused lymphodepletion than anti‑CD7 CAR T cells

**Fig. 5:**
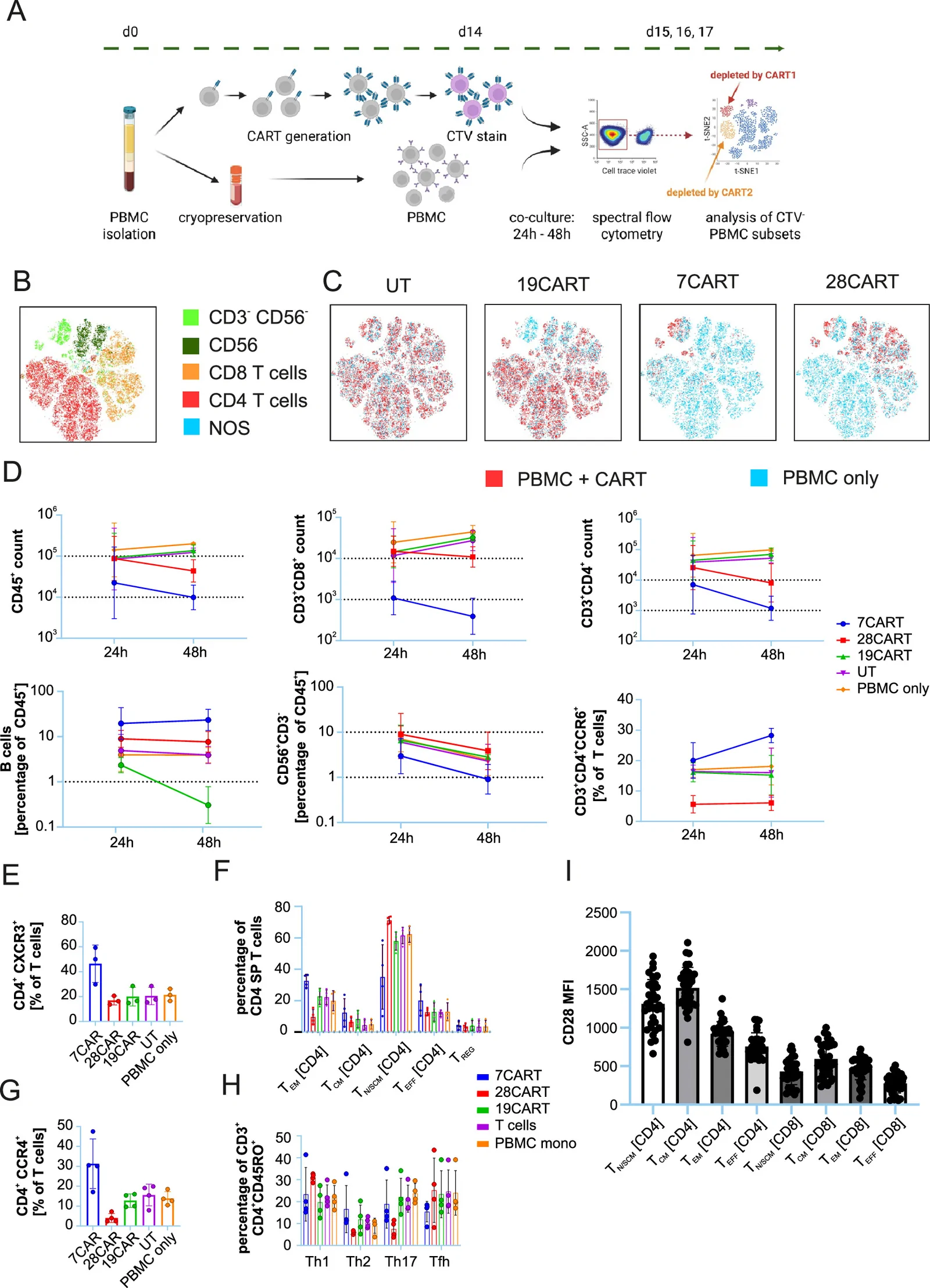
Anti-CD28 CAR T cells preferentially deplete physiological Th2 and Th17 cells while sparing CD8 T cells compared with anti-CD7 CAR T cells. **A** Workflow for autologous PBMC–CAR T cell co-cultures and cytometric assessment of lymphodepletion. Created in BioRender. Burk, U. (2026) https://BioRender.com/i0mvula. **B** t-SNE of CTV-negative PBMCs cultured alone, identifying major lymphocyte populations. CD3^−^CD56^−^ lymphocytes predominantly comprise B cells. NOS not otherwise specified. **C** t-SNE overlays of PBMCs co-cultured with autologous CAR T cells. UT T cells did not alter PBMC subsets. Anti-CD19 CAR T cells depleted CD3^−^CD56^−^ lymphocytes; anti-CD7 CAR T cells depleted CD4 and CD8 T cells and NK cells; anti-CD28 CAR T cells preferentially depleted CD4 T cells. **D** Absolute CD45^+^, CD4^+^ and CD8^+^ T cell counts and frequencies of B cells, NK cells and CCR6^+^CD4^+^ T cells after 24 and 48 h. Anti-CD7 CAR T cells produced broader depletion, whereas anti-CD28 CAR T cells preferentially depleted CCR6^+^CD4^+^ T cells. B cells were defined as CD3^−^CD56^−^HLA-DR^hi^CXCR5^+^ cells. **E** CXCR3^+^CD4^+^ T-cell frequency, a Th1 correlate, was unchanged after anti-CD28 CAR T-cell co-culture. **F** After 48 h co-culture, CD4 T-cell depletion was concentrated in effector-memory CD4 T cells, defined as CD45RO^+^CD197^-^ helper T cells (33, 10, 23, 22, and 20% for 7CAR, 28CAR, 19CAR, UT, and PBMC-only, respectively; Kruskal–Wallis test, *p* = 0.0058). **G** CCR4^+^ CD4 T cell frequency was reduced after 48 h of co-culture with anti-CD28 CAR T cells (31%, 4%, 13%, 16%, and 14% for 7CAR, 28CAR, 19CAR, UT, and PBMC-only, respectively; Kruskal–Wallis test, *p* = 0.0077). **H** Frequency alteration after CAR T cell co-culture of Th1 (CD3^+^CD4^+^CD8^−^CD45RO^+^CXCR3^+^CCR4^−^CCR6^−^), Th2 (CD3^+^CD4^+^CD8^−^CD45RO^+^CXCR3^−^CCR4^+^CCR6^−^), Th17 (CD3^+^CD4^+^CD8^−^CD45RO^+^CXCR3^−^CCR6^+^CCR4^−^) and follicular helper CD4 T cells (CD3^+^CD4^+^CD8^−^CD45RO^+^CXCR5^+^). Anti-CD28 CAR T cells preferentially depleted Th2 (*p* = 0.028) and Th17 cells (*p* = 0.040; two-sided Friedman tests). **I** CD28 surface expression, measured as MFI, was highest in CD45RO^+^ CD4 T cell subsets (T_CM_ and T_EM_). Physiologic bone marrow T cells from healthy donors or patients with T-ALL (cf. Table 1). Data are mean ± s.d. from 3 (**D**, **E**) or 4 (**F**–**H**) independent healthy donors or 35 donors (**I**). Source data are provided as a Source data file.

T cell depletion represents a major hurdle for CAR T cell therapy against T-ALL antigens shared by physiologic T cells and, indeed, fatal viral infections have been described following anti-CD7 CAR T cell therapy^31^. Therefore, we sought to compare features of lymphodepletion conferred by anti-CD28 and anti-CD7 CAR T cell treatment. Co-cultures of CellTrace Violet (CTV)-labeled CAR T cells with autologous PBMCs were established (Fig. 5A), and CTV⁻ PBMCs were analyzed after 24 h and 48 h using a 25-color spectral flow panel adapted from OMIP-071 (Supplementary Table 4)^32^. Using anti-CD19 CAR T cells as a benchmark, we observed the expected depletion of B cells compared with PBMCs co-cultured with untransduced T cells (Fig. 5B–D).

First, CD28 surface expression was almost abolished in remaining CD4^+^ T, while CD28 expression in CD8^+^ T cells and CD7 expression in both CD4^+^ and CD8^+^ T cells was reduced ~50% after 48 h of co-culture (Supplementary Fig. 13A). Total lymphocyte counts were reduced by anti-CD28 CAR T cells only at 48 h, but not at 24 h, and then remained approximately fivefold higher than after anti-CD7 CAR T cell exposure (Fig. 5D, top left). A similar pattern was seen for CD4⁺ T cells, whereas CD8⁺ T cell numbers were only mildly affected by anti-CD28 CAR T cells but were depleted more than tenfold by anti-CD7 CAR T cells (Fig. 5D, top middle and right). CD56⁺CD3⁻ NK cells were depleted exclusively by anti-CD7 CAR T cells, while anti-CD28 CAR T cells were associated with a relative increase in NK-cell frequency (Fig. 5D). These findings are consistent with CD28 surface expression patterns in healthy donor and T-ALL bone marrow, showing significantly lower CD28 expression on CD8⁺ compared with CD4⁺ T cells and homogeneous CD7 expression in CD4^+^ and CD8^+^ T cells (Fig. 5I, Supplementary Fig. 13B).

Concurrently, the frequencies of CD3⁺CD4⁺CCR6⁺ and CD3⁺CD4⁺CCR4⁺ T cells were markedly reduced by anti-CD28 CAR T cells, whereas CD4⁺CXCR3⁺ T cell frequencies remained unchanged in these co-cultures (Fig. 5E, G). In line with CD28 surface expression data from healthy bone marrow donors, we observed preferential depletion of T_EM_ CD4^+^ T cells with a compensatory enrichment of T_N/SCM_ 48 h after co-culture (Fig. 5F, I) by anti-CD28 CAR T cells. Finally, combinatorial gating on Th1, Th2, Th17 and follicular helper T cells (identified by use of surface markers CD4, CD45RO, CXCR3, CCR4, CCR6 and CXCR5) confirmed preferential depletion of Th2 and Th17 T cells by anti-CD28 CAR T cells (Fig. 5H).

## Discussion

The clinical experience of anti-CD19 CAR T cells has revolutionized immunotherapy for malignant diseases, although in the majority of high-risk patients significant challenges remain on the path to long-lasting remissions. Immunotherapy of T-ALL involves additional challenges such as shared target antigen expression by T-ALL and healthy T cells. While anti-CD7 CAR T cells have shown great promise in clinical trials, long-term remission due to CAR T cells is only achieved in a minority of patients. This is why additional therapies are necessary, in particular therapies suitable for highly pre-treated patients with T-ALL.

We have identified CD28 to be highly overexpressed in both the majority of T-ALL from children and adolescents and in nodal T follicular helper cell lymphoma. We have additionally found that ligation of CD28 on T-ALL cell lines leads to partial resistance to JAK inhibition by ruxolitinib, suggesting a JAK-independent pro-survival program downstream of CD28. These findings support that CD28 bears functional relevance in T-ALL, as has been described both in healthy T cells^9^, malignant T cells^18^, and other neoplasias^23^. CD28 KO in primary human T cells showed unaltered expansion after activation and retained effector functionality. Two anti-CD28 CAR molecules conferred high and specific cytotoxicity of primary human T cells in vitro against two T-ALL cell lines as well as in three T-ALL xenograft mouse models, including a patient-derived xenograft (PDX) model. 28CAR_2 CAR T cells outperformed 28CAR_14 CAR T cells and proved equally efficient as anti-CD7 CAR T cells in T-ALL xenograft mouse models.

We observed CD28 expression to be significantly reduced in CCRF-CEM cells harvested from mouse bone marrow and spleen upon sacrifice, while this was not the case for CD7. Interestingly, two alternative T-ALL mouse models using T-ALL cell line Jurkat or ALL-230 PDX cells did not show reduced CD28 surface expression upon T-ALL occurrence after CAR T cell therapy, indicating that CD28 loss under selective pressure can happen in some T-ALL cells. Interestingly, internalization of CD28 upon soluble antibody exposure was pronounced in Jurkat cells when compared to CCRF-CEM in vitro (Supplementary Fig. 14). This indicates that CD28 downregulation is a short-term effect observed in vitro, while CD28 immune escape occurs by a distinct mechanism.

In 2006, a clinical trial using a CD28 targeting antibody designed to expand regulatory T cells led to a non-intentional cytokine storm in 6/6 healthy volunteers that resulted in life-threatening disease^28^. In light of this unexpected side effect at the time, we performed co-culture studies of WT T cells with anti-CD28 CAR T cells and subsequently analyzed activation of WT T cells. Importantly, we did not observe any signs of T cell activation when the scFv of TGN1412 was used in comparison to other anti-CD28 CAR molecules. This indicates that the elimination speed of CD28^+^ cells is such that these cells are eliminated or at least interrupted in their function, before CD28 ligation by superagonistic TGN1412 triggers uncontrolled T cell activation.

Anti-CD28 CAR T cells induced a more selective and overall milder lymphodepletion than anti-CD7 CAR T cells, sparing CD8⁺ T cells and NK cells while preferentially depleting CD28-high CD4⁺ effector subsets, in particular Th2 and Th17 cells. This pattern, which aligns with CD28 expression in healthy donor and T-ALL bone marrow, suggests that anti-CD28 CAR T cell therapy may reduce the risk of profound pan-lymphocytic immunosuppression associated with anti-CD7 CAR T cells. Indeed, a recent clinical study of anti-CD7 CAR T cells for T-ALL documented a patient who died of an infection while still in remission^33^. This observation underlines the clinical relevance of anti-T-cell CAR T cell therapy-mediated immune deficiency. Of note, CD28^+^ long-lived plasma cells are likely to be compromised by anti-CD28 CAR T cells, which could necessitate immunoglobulin substitution and re-vaccination^34^.

In line with anti-CD7 CAR T cells, anti-CD28 CAR T cells could be used for T-ALL eradication prior to allogeneic stem cell transplantation, such as a bridge to transplant. However, as a recent landmark study illustrated, anti-CD7 CAR T cell therapy as a standalone approach, where CD7^−^ physiologic T cells expanded after CAR T cell infusion and retained relevant T cell effector functions^6^. With regard to the overall milder lymphodepletion conferred by anti-CD28 CAR T cells, it seems reasonable to envision this immunotherapy as a standalone therapy, too. In addition, patients with CD7-negative relapse after anti-CD7 CAR T cell therapy would profit from an alternative immunotherapy.

Together with the observation that CD28-deficient T cells retain effector functions, this sets the stage for CD28 to be used as a target for cancer immunotherapy. Even more so, as CD28 represents an immunotarget not only for (pediatric) T-ALL but also for a subset of T cell lymphomas and even for a non-T cell malignancy, multiple myeloma. Of interest, in the latter, a functional contribution of CD28 to multiple myeloma pathogenesis has been demonstrated recently^35^.

In conclusion, CD28 represents a target for synthetic immunotherapy against T-ALL, yielding anti-T-ALL efficacy comparable to that of anti-CD7 CAR T cells with reduced depletion of physiologic T cells. The extent of anti-CD28 CAR-mediated immunodeficiency and efficacy against T-lineage malignancies will require evaluation in clinical trials.

## Methods

This study was approved by the Institutional Ethical Review Board (“Ethikkommission bei der LMU München”, approval number 17-163) or by the ETHIK-KOMMISSION of the Albert-Ludwigs-University of Freiburg (approval number 24-1047_3-S1). The study was performed in accordance with the Declaration of Helsinki.

### Patients

Patient and healthy donor characteristics are summarized in Table 1. Written informed consent for research use of biological material was obtained from patients and/or their legal guardians for all samples obtained through the Cooperative Study Group for Childhood Acute Lymphoblastic Leukemia (CoALL) and for the majority of samples obtained through the Hauner Hematology Biobank. For a limited number of clinically archived samples for which re-contacting the patient and/or legal guardians to obtain consent was not reasonably feasible (e.g., because the patient had died or could no longer be contacted), the ethics approval permitted use of anonymized residual material without individual informed consent. Bone marrow samples from patients with T-ALL and healthy bone marrow donors were provided by the Hauner Hematology Biobank and CoALL biobank. All samples were exhausted during the analyses. Healthy bone marrow donors were age-matched children without evidence of hematologic disease who underwent bone marrow aspiration for donor evaluation.

### CD28 immunohistochemistry

For immunohistochemistry, bone marrow was decalcified, fixed in 4% paraformaldehyde, and embedded in paraffin. After antigen retrieval with antigen retrieval buffer (pH = 9; AR9 Akoya Bioscience), sections were incubated with anti-CD28 (Abcam ab243228, clone EPR22076 1:750 dilution). Staining was detected using the ImmPRESS Polymer Kit (Vector) according to the manufacturer’s protocol.

### Flow cytometry

All patient samples analyzed in this project were subjected to mononuclear cell isolation using density centrifugation (Biocoll, Merck), cryopreservation with 5% human serum albumin (Biotest Pharma) and 10% dimethyl sulfoxide (Sigma-Aldrich), and storage in liquid nitrogen. Prior to analysis, samples were thawed, washed in phosphate-buffered saline (PBS) once, and subsequently subjected to flow cytometry staining using the antibodies documented in Supplementary Table 1. Flow cytometry analysis was performed on a BD LSR Fortessa (Becton, Dickinson and Company) and on the MACSQuant Analyzer 10 (Miltenyi Biotec). Spectral flow cytometry was performed using the ID7000 Spectral Cell Analyzer (Sony Biotechnology). Flow cytometry data was analyzed using FlowJo 10.10.0 (FlowJo LLC) and the ID7000 software (Sony Biotechnology). Lymphocyte gating was performed in an SSC-A vs. FSC-A plot to exclude debris; doublets were excluded using an FSC-H vs. FSC-A plot, and only viability-dye (7AAD) negative cells were gated as living cells. MFI values were reported as untransformed raw values and compared only among samples acquired using identical instrument settings.

To determine intracellular cytokine accumulation upon target-cell contact, T cells were co-cultured with CD28^+^ CCRF-CEM cells at an E:T ratio of 1:1. Intracellular cytokine staining was performed after 24 h with addition of brefeldin A (Sigma-Aldrich) 4 h before staining. Subsequently, cells were stained for surface markers, fixed and permeabilized using the Invitrogen™ eBioscience™ Intracellular Fixation & Permeabilization Buffer Set (Cat. No. 88-8824-00; Thermo Fisher Scientific) according to the manufacturer’s instructions. Intracellular staining was performed using fluorochrome-conjugated antibodies against TNF-α, IFN-γ, and granzyme B, followed by flow-cytometric acquisition and analysis.

Flow-cytometric analyses were performed after sequential gating on lymphocytes based on forward- and side-scatter properties, singlets, and viable cells. T-ALL blasts were identified as CD45^dim^ surface CD3-negative (sCD3^−^) CD7-positive cells. Healthy lymphoid progenitors from bone marrow donors were identified using the same gating strategy. Physiological T cells were defined as viable singlet lymphocytes expressing surface CD3; CD4^+^ and CD8^+^ T-cell subsets were defined as CD3^+^CD4^+^CD8^−^ and CD3^+^CD4^−^CD8^+^ cells, respectively. B cells were defined within viable singlet lymphocytes as CD3^−^CD56^−^HLA-DR^hi^CXCR5^+^ cells. For autologous PBMC–CAR T-cell co-culture experiments, CTV-negative cells were gated before subsequent identification of lymphocyte subsets, thereby excluding CTV-labeled CAR T cells and untransduced T-cell controls from the analysis. Th1, Th2, Th17 and T follicular helper-like cells were defined among CD3^+^CD4^+^CD8^-^CD45RO^+^ T cells as Th1 (CXCR3^+^CCR4^−^CCR6^−^), Th2 (CXCR3^−^CCR4^+^CCR6^−^), Th17 (CXCR3^−^CCR4^−^CCR6^+^) and T follicular helper-like cells (CXCR5^+^), respectively. In xenograft experiments, leukemia cells recovered from mouse tissues were identified after gating on lymphocytes, singlets, and viable cells as GFP-positive CD45^+^ events for CCRF-CEM and Jurkat cells or mCherry-positive CD45^+^ events for ALL-230 PDX cells. Persisting human CAR T cells recovered from mouse tissues were identified after gating on lymphocytes, singlets, and viable cells as GFP-negative CD45^+^CD3^+^ events. In co-culture experiments with GFP-labeled T-ALL cells, T cells were identified after gating on lymphocytes, singlets, and viable cells as GFP-negative CD3^+^ cells and further subdivided into CD4^+^CD8^−^ and CD4^−^CD8^+^ T-cell populations.

t-Distributed stochastic neighbor embedding (t-SNE) analysis. High-dimensional flow-cytometry data from autologous PBMC–CAR T-cell co-culture experiments were analyzed using the built-in t-distributed stochastic neighbor embedding (t-SNE) algorithm in FlowJo v10.10.0 (BD Life Sciences). Following exclusion of debris, doublets, and non-viable events, CTV-negative cells were selected to exclude CTV-labeled CAR T cells or untransduced T-cell controls. A maximum of 100,000 randomly downsampled CTV-negative events per condition were obtained from PBMC-only cultures and PBMCs co-cultured with untransduced T cells, anti-CD19 CAR T cells, anti-CD7 CAR T cells, or anti-CD28 CAR T cells. Events from all five conditions were concatenated before performing a joint t-SNE analysis, enabling direct visualization of phenotypic differences between conditions in the same embedding. The t-SNE calculation included compensated expression values for CD3, CD4, CD8, CD45RO, CD197, CXCR3, CCR4, CCR6, CXCR5, CD56 and HLA-DR. Forward- and side-scatter parameters, viability dye, CTV, and markers not listed above were excluded from the t-SNE calculation. The analysis was performed using default FlowJo t-SNE settings. t-SNE maps were used for visualization and population annotation only; subset frequencies and absolute cell counts were determined by conventional manually gated flow-cytometry analyses.

### Isolation of peripheral blood mononuclear cells (PBMCs) and T cell activation

PBMCs were isolated from peripheral blood of healthy donors by density gradient centrifugation (Biocoll). To avoid interference from magnetic beads attached to the T cells during electroporation, we chose an untouched T cell isolation using the EasySep Human T Cell Enrichment Kit (STEMCELL Technologies) according to the supplier’s information. T cells were activated using T Cell TransAct, human (Miltenyi Biotec) according to the manufacturer’s information. T cells were cultured in TexMACS medium (Miltenyi Biotec)/ 2.5% human AB serum (Institute for Clinical Transfusion Medicine, Ulm, Germany) + 12.5 ng/ml human IL-7 and IL-15, premium grade (Miltenyi Biotec), and split every 2–3 days.

### Cell lines

The human T-ALL cell lines CCRF-CEM (Leibniz Institute DSMZ–German Collection of Microorganisms and Cell Cultures GmbH, ACC 240) and Jurkat (DSMZ, ACC 282), the B-ALL cell line Nalm6 (DSMZ, ACC 128) and the human multiple myeloma cell line RPMI-8226 (DSMZ, ACC 402), were obtained from DSMZ (Braunschweig, Germany). The human multiple myeloma cell line MM.1S was obtained from the American Type Culture Collection (ATCC, CRL-2974). The retroviral packaging cell lines 293Vec-Galv and 293Vec-RD114 were kindly provided by BioVec Pharma Inc. (Canada). The patient-derived T-ALL xenograft (PDX) model ALL-230, stably expressing enhanced firefly luciferase and mCherry, was kindly provided by Irmela Jeremias. No cell lines were newly established from primary human or vertebrate cells in this study. The sex of the original donor of the ALL-230 PDX model was not available to the authors.

CCRF-CEM, Jurkat, Nalm6, MM.1S and RPMI-8226 were cultured in RPMI 1640 medium (Bio&SELL GmbH) supplemented with 10% fetal bovine serum (Sigma-Aldrich), 1% penicillin-streptomycin (Gibco) and 1% L-glutamine (200 mM; Biochrom). Cell-line identity was confirmed by short tandem repeat profiling (DSMZ), and cell lines were routinely tested for mycoplasma contamination using the VenorGEM Mycoplasma Detection PCR kit (Minerva Biolabs).

### CD28 and CD7 knockout in primary T cells and T-ALL cell lines

CD28 and CD7 knockout in primary T cells and T-ALL cell lines. CRISPR/Cas9-mediated knockout of CD7 or CD28 was performed 2 days after T-cell activation. Alt-R CRISPR-Cas9 tracrRNA and Alt-R CRISPR-Cas9 crRNA targeting CD7 (5′-CCAAGACATCATTTACTACG-3′) or CD28 (5′-GCTTGTAGCGTACGACAATG-3′) were synthesized by Integrated DNA Technologies. For ribonucleoprotein (RNP) complex formation, 50 pmol crRNA and 50 pmol tracrRNA were mixed at a 1:1 molar ratio and annealed by heating for 5 min at 95 °C. The annealed guide RNA was combined with 50 pmol Alt-R S.p. Cas9 Nuclease 3NLS (Integrated DNA Technologies) and 100 pmol Alt-R Cas9 Electroporation Enhancer (Integrated DNA Technologies) and incubated for 15 min at room temperature.

For each electroporation, 1 × 10^6^ activated T cells were resuspended in 15 µl P3 Primary Cell Nucleofector Solution supplemented according to the manufacturer’s instructions (Lonza). Five microliters of the RNP mixture were added, and cells were electroporated in a 20-µl Nucleocuvette Strip using a 4D-Nucleofector device (Lonza) with program EO-115. Immediately after electroporation, cells were transferred to pre-warmed fresh medium. Cells electroporated with a non-targeting control crRNA were used as controls. Editing efficiency was assessed by amplicon sequencing of the CD7 and CD28 target loci and by flow-cytometric quantification of surface CD7 and CD28 expression.

### Design of CD28-directed chimeric antigen receptors (CARs)

CARs were designed as published previously^36^. Single-chain variable fragments were derived from publicly available sources: CD28.3 from GenBank under accession number AF451974, TGN1412 from patent US008709414B2, and cl184, cl83, and CD28-scFv#5 from other publications^37,38^. Both sequences of the heavy and light variable fragments were tested with both a CD8a-derived long hinge domain (FVPVFLPAKPTTTPAPRPPTPAPTIASQPLSLRPEACRPA-AGGAVHTRGLDFACD) or a short linker (GGGGS)_3_. A published anti-CD19 CAR sequence was used as a scaffold^36^. Anti-CD28 CAR molecule design is detailed in Table 2. Alternatively to the CD28 costimulatory domain, 41BB or combined CD28 + 41BB (28BB) costimulatory intracellular sequences were used. Either an extracellular myc tag or a F2A-linked truncated epidermal growth factor receptor (EGFRt) was used for validation of T cell transduction. CAR molecules without tag were stained with recombinant FITC-labeled human CD28 (AcroBio).

### Retroviral transduction of primary T cells

For retroviral transduction, 2nd generation anti-CD19, anti-CD7 and anti-CD28 CAR sequences containing FMC63, 3A1f or one of five CD28 scFvs containing CD8 transmembrane and spacer domains and either 41BB, CD28 or 28BB costimulatory domain were cloned into pMP71 (kindly provided by Christopher Baum, Medizinische Hochschule Hannover, Hannover, Germany) via EcoRI and NotI using Gibson Assembly® Master Mix (NEB)^39^. A myc tag (EQKLISEEDL) was included in the CAR for detection and purification. CAR molecules cloned into pMP71 were transfected into 293Vec-Galv cells (kindly provided by BioVec Pharma Inc) using TransIT-293 Transfection Reagent (Mirus Bio) according to the supplier’s information with 2.5 µg of plasmid DNA. Retroviral supernatant was used for transduction of 293Vec-RD114 cells (kindly provided by BioVec Pharma Inc) to create a stable producer system, which were subsequently FACS-sorted for myc-positive cells. Viral titers were not routinely determined; transduction efficiency was assessed by flow cytometry. Retroviral supernatant of 293Vec-RD114 cells was used to transduce primary T cells from healthy donors. Therefore, 24-well plates were coated with 2.5 μg RetroNectin Reagent (Takara Bio) per well at 37 °C for 2 h. Plates were blocked with 2% Albumin Fraction V (Carl Roth) in PBS (Gibco) for 30 min and washed with a 1:40 dilution of HEPES 1 M (Thermo Fisher Scientific) in PBS. Virus supernatant was harvested and filtered (0.45 μm). 1 ml virus supernatant was transferred into each well of the plate and centrifuged at 3000 × *g* for 90 min at 32 °C. Virus supernatant was discarded, and 1 × 10^6^ T cells in 1 ml TexMACS medium (Miltenyi Biotec) + 2.5% human AB serum (Institute for Clinical Transfusion Medicine, Ulm, Germany) + 12.5 ng/ml human IL-7 and IL-15 (Miltenyi Biotec) + 2 μg/ml Protamine sulfate (Sigma-Aldrich) were added. Plates were centrifuged for 10 min at 450 × *g*, 32 °C. Transduction was performed on day 2 after T cell activation. When additional CRISPR/Cas9-mediated knockout was performed, transduction was performed on day 4 after T cell activation.

### Co-Culture experiments in vitro

CD7^+^/CD28^+^ CCRF-CEM or Jurkat cells were either labeled according to the CellTrace Violet Cell Proliferation Kit (Thermo Fisher Scientific) or transduced with a lentivirus coding for GFP and luciferase (Addgene #104834; virus production in HEK293 cells using #8454 and #104834 with subsequent sort of GFP^+^ cells). Next, they were co-cultured with untransduced T cells or CAR T cells for 24–48 h at different E:T ratios. Specific cytotoxicity (%) = [1 − viable target-cell count in co-culture/viable target-cell count in target-only control] × 100. To that end, either target cell count was measured on a MACSQuant Analyzer 10 (Miltenyi Biotec) or luminescence was quantified 5 min after addition of Bright-Glo^TM^ (Promega) using the LumistarOmega (BMG Labtech). As an alternative for T cell engagement of leukemia, CD3/CD19 bispecific T cell engager blinatumomab (Amgen) was used at 0.5 µg/ml.

In addition to the above-mentioned one-time readouts, live cell imaging was performed using the Incucyte SX5 platform. If not stated otherwise, 3.5 × 10^4^ cells per well in 200 µl on a 96-well plate were either cultured alone (T-ALL cell line) or together with CAR T cells at specified effector-to-target ratios. For cell-by-cell analysis, 20× objective imaging was performed to differentiate, e.g., GFP-labeled T-ALL cells and non-GFP-labeled CAR T cells. Quantification of cellular abundance was performed as recommended by measuring GFP integrated intensity in at least technical triplicates (Sartorius, Incucyte Software Version 2023A-2025B). Incucyte GFP integrated intensity was normalized to the first measurement to provide a “relative T-ALL” count (e.g., in Supplementary Fig. 17). For repetitive killing, 3.5 × 10^4^ Jurkat cells were added every 48–72 h, and medium was replenished, including any antibody previously present in the co-culture.

For Interferon-y blockade, TNFa-blockade or LFA-1-blockade a blocking antibody against IFNγ (Cat. 14-7318-81, ThermoFisher) at 1 µg/ml, against TNFa (clone: MAB2101, Cat: MAB2101-100, R&D Systems) at 10 µg/ml or against LFA-1 (LFA‑1α; BE0192, Bio X Cell) at 10 µg/ml were used with a control IgG1-antibody as control (MAB002, R&D Systems).

Generation of monocyte-derived dendritic cells and co-culture assays. CD14-positive monocytes were isolated from healthy-donor PBMCs using CD14 MicroBeads and LS columns (Miltenyi Biotec) according to the manufacturer’s instructions. Briefly, PBMCs were incubated with 20 µl MicroBeads per 1 × 10^7^ cells for 15 min at 4 °C, and CD14-positive cells were purified by magnetic separation. Monocytes were cultured at 1 × 10^6^ cells/ml in RPMI1640 in six-well plates. On day 0, human IL-4 (125 ng/ml; Miltenyi Biotec, Cat. No. 130-093-917) and human GM-CSF (400 ng/ml; Miltenyi Biotec, Cat. No. 130-095-372) were added. On day 2 or 3, maturation was induced by addition of human IL-6 (100 ng/ml, Miltenyi Biotec, 130-093-929), human IL-1β (40 ng/ml, Miltenyi Biotec, 130-095-374), human TNF-α (100 ng/ml), and PGE2 (2 µg/ml, Stemcell, Catalog # 72194) for at least 48 h. Mature moDCs were validated by flow cytometry, including CD80 and CD86 expression, and CCRF-CEM cells were subsequently co-cultured at a 1:1 ratio (5 × 10^3^ cells each per well) in medium containing 2% FCS. Leukemia-cell proliferation was monitored by Incucyte SX5 live-cell imaging in technical triplicates

For co-culture assay of PBMCs with autologous CAR T cells, 1 × 10^6^ CTV-labeled CAR T cells or untransduced T cells were co-cultured at 1:1 effector to target (E:T) ratio with corresponding PBMCs in a 6-well dish. After 24 and 48 h, cells were harvested and stained with a 25-color spectral panel (Supplementary Table 4) before all events were recorded at the ID7000 spectral analyzer (Sony Biotechnology).

### Cellular avidity analysis

Jurkat cells were subjected to CRISPR/Cas9 mediated knockout of CD28 as described above and then retrovirally transduced with either 28CAR_2 or 28CAR_14 (cf. Table 2; abbreviations of anti-CD28 CAR molecules will be employed from here onward). Transduced Jurkat cells were repeatedly FACS-sorted using a myc tag proximal to the scFv until a CAR surface expression of >97% was reached for both samples. Next, CD28^+^ Jurkat cells were used as target cells, and after preparation of a z-Movi chip using Poly-L-Lysine seeded at a concentration of 1.8 × 10^8^/ml^40^. Subsequently, Jurkat cells transduced with either 28CAR_2 or 28CAR_14 were stained with CellTrace Far Red (Thermo Fisher Scientific), flushed into the chip, and co-incubated with the target cells for 4 min until a gradually increasing force was applied by the piezo element of the z-Movi device. Continuous imaging was performed to determine remaining CellTrace Far Red positive effector cells as a measure of cellular avidity.

### *direct* Stochastic Optical Reconstruction Microscopy

For *d*STORM analysis, purified anti-c-Myc antibody was purchased from Biolegend (Cat. 626802) and labeled with Alexa Fluor 647 using the AF647-NHS ester kit. 8-well chambered cover glasses were treated with 1 M KOH and coated with PDL. CAR T cells were washed and resuspended in FACS buffer and labeled using 10 µg/ml anti-c-Myc-AF647 antibody for 30 min at 4 °C in the dark. Labeled cells were washed six times to remove unbound antibody. Cells were plated in coated cover glass chambers and allowed to adhere for 15 min on ice and fixed in PBS supplemented with 3% formaldehyde and 0.25% glutaraldehyde for 15 min at RT. For imaging, PBS supplemented with 100 mM cysteamine hydrochloride (pH 7.4) was used. Image acquisition was carried out on an ONI Nanoimager S in total internal reflection fluorescence mode with a pixel size of 117 nm. *d*STORM image reconstruction and linear drift correction was performed with rapidSTORM 3.3^41^. Cell regions of interest were selected using Napari. Cluster analysis was performed using LOCAN—a custom localization analysis tool—using a density-based spatial clustering of applications with noise (DBSCAN) algorithm^42^.

### T-ALL mouse model

All animal experiments were performed in accordance with applicable German animal welfare legislation and were approved by the responsible governmental authorities. Experiments evaluating CD28 CAR-T cells with different costimulatory domains (Fig. 4a–e, i, j) and the dose-finding experiment (Supplementary Fig. 10a–d) were performed at the Ludwig Maximilian University of Munich and approved by the Government of Upper Bavaria (Regierung von Oberbayern; protocol no. ROB-55.2-2532.Vet_02-21-58, including the approved amendment of 23 May 2023). All other in vivo experiments, including Fig. 4f–h, k and Supplementary Fig. 11a–e, were performed at the University Medical Center Freiburg and approved by the Regierungspräsidium Freiburg, Abteilung 3, Referat 35 (protocol no. G25-001). All animal experiments were performed using female and male NXG mice (*Mus musculus*; NOD- Prkdcscid-IL2rgTm1/Rj; NOD genetic background; Janvier Labs), aged 6–12 weeks at study initiation. Mice were maintained under facility-specific specific pathogen-free conditions, had free access to food and water, and were housed under a 12-h light/12-h dark cycle, with the dark phase from 18:00 to 06:00, at an ambient temperature of 20–24 °C and relative humidity of 45–65%. Mice were monitored at least three times weekly, including body-weight measurements, and were monitored daily when abnormalities were observed. Predefined humane endpoints included ≥20% body-weight loss within 72 h, apathy, abdominal breathing/dyspnea, paralysis or visible dragging of the hind limbs, complete loss of the escape response, or a 10–15% body-weight loss relative to the previously recorded maximum body weight persisting for 72 h in combination with a dull coat, reduced hind-limb mobility, or persistent diarrhea. Mice displaying early neurological abnormalities, including gait instability, reduced hind-limb extension, or reduced rearing, were monitored at least daily. Mice were euthanized upon reaching any predefined humane endpoint, when bioluminescence imaging exceeded 1 × 10^10 photons/s, or at the planned experimental endpoint. Mice had free access to food and water. The leukemia cell lines CCRF-CEM and Jurkat and the PDX model ALL-230 were genetically modified by lentiviral transduction to express enhanced firefly luciferase and enhanced GFP (eGFP) or mCherry as a selection marker (Addgene #104834 or #104833). A total of 7.5 × 10^4^ (CCRF-CEM, experiment illustrated in Fig. 4) to 1.0 × 10^6^ (experiments in Supplementary Fig. 11) luciferase-positive tumor cells in 100 µl of PBS were transplanted into NOD-Prkdcscid-IL2rgTm1/Rj (NXG, Janvier) by tail vein injection. Four days after injection, CAR T cells were thawed and counted, and 2.5 to 5.0 × 10^6^ CAR T cells in 100 µl of PBS were injected into mice by tail vein injection. Leukemia burden was monitored once per week by bioluminescence in vivo imaging (BLI) as described previously^43^. Peripheral blood was analyzed weekly to detect the presence of CAR T cells, and mice were monitored daily for weight loss, hair loss, altered posture, or reduced mobility according to scoring criteria. When BLI exceeded 1 × 10^10^ photons/s or when mice reached a predefined humane endpoint, they were euthanized, and bone marrow, spleen, and peripheral blood were harvested for subsequent analyses. In mice injected with Jurkat cells, BLI signals did not correspond well with mouse well-being, leading to a difference in survival analysis and BLI analysis. In order to justify the survival data, we included individual mouse weights for mice injected with Jurkat cells, illustrating that mice injected with anti-CD19 CAR T cells showed earlier weight loss than mice treated with either anti-CD7 or anti-CD28 CAR T cells. Initially, we tested different CCRF-CEM dosages and determined 7.5 × 10^4^ CCRF-CEM as a suitable dose that was injected into mice via the tail vein 4 days prior to injection of 2.5 × 10^6^ CAR T cells targeting either CD19, CD7, or CD28 (Supplementary Fig. 10A–D)^39^. Here, we observed significantly increased survival of mice treated with either anti-CD7 or anti-CD28 CAR T cells compared to anti-CD19 CAR T cells as controls (median survival 34 days for 7CAR, 38 days for 28CAR_2, and 22 days for 19CAR T cell treatment).

### Statistics

Statistical significance of surface marker expression was determined using Prism 10.6.1 and the Mann–Whitney test or the Kruskal-Wallis test or the Friedman test if more than two groups were compared, as our sample size was not sufficient to test for normal distribution (GraphPad Prism 10.6.1). For testing the statistical significance of treatment vs. control groups in vivo, a two-way ANOVA was performed as implemented in GraphPad Prism 10.6.1. Survival analysis was performed using the Log-rank (Mantel-Cox) test to determine statistical significance for survival curve comparison (GraphPad Prism 10.6.1). Significance levels are illustrated by asterisks: * ~ *p* ≤ 0.05, ** ~ *p* ≤ 0.01 and *** ~ *p* ≤ 0.001. Simple linear regression using GraphPad Prism 10 was performed to compare 2B4 and CD28 surface expression on T-ALL blasts.

Unless otherwise indicated, in vitro experiments were performed using cells from at least three biologically independent healthy donors. Technical replicates were averaged before statistical analysis and were not treated as independent biological replicates. Each dot represents one biologically independent donor, patient sample, or mouse, as specified in the figure legends. Data are shown as mean ± s.d., unless otherwise indicated. Time-course data from technical triplicates are presented as median ± s.e.m unless otherwise specified.

### Reporting summary

Further information on research design is available in the Nature Portfolio Reporting Summary linked to this article.

## Supplementary information

Supplementary Information

Reporting Summary

Transparent Peer Review file

## Source data

Source data

## Data Availability

Raw flow cytometry data (FCS files) generated from human donor and patient samples are not publicly available because the consent forms and biobank governance frameworks restrict unrestricted sharing of potentially re-identifiable data from small pediatric cohorts. De-identified aggregated data underlying the figures are provided in the main text, Supplementary Information, and Source data files. De-identified raw FCS files may be made available to qualified researchers for non-commercial research upon reasonable request to the corresponding author, subject to review by the responsible institutional biobank and execution of an appropriate data use agreement. Requests will be considered within 4 weeks of receipt. The microarray dataset GSE13159 from the MILE study is publicly available through the Gene Expression Omnibus under accession code GSE13159 (https://www.ncbi.nlm.nih.gov/geo/query/acc.cgi?acc=GSE13159; https://www.fobinf.com/)^22^. The remaining data are available within the article, Supplementary Information, or Source data file. Source data are provided with this paper.
