# Supplementary material for: Targeting CD28 on T lineage malignancies with chimeric antigen receptor T cells
Source: Nat Commun. 2026 Oct 8;17:10608. doi: 10.1038/s41467-026-78312-3 (PMC13646262; doi:10.1038/s41467-026-78312-3)
Supplement: Supplementary file 1 [file 41467_2026_78312_MOESM1_ESM.pdf]

Supplementary Information

Supplementary Tables

Supplementary Table 1: Antibodies used in this study

Antigen	Fluorochrome	Clone	Catalogue number	Manufacturer	Dilution
Viability dye	7AAD	-	420404	Biolegend	1:100
CD8	APC	SK1	556414	Becton, Dickinson and Company (BD)	1:200
CD28	APC	CD28.2	559770	Becton, Dickinson and Company (BD)	1:20
CD56	APC	NCAM16.2	341027	Becton, Dickinson and Company (BD)	1:100
CD86	APC	IT2.2	305411	Biolegend	1:50
CD95	APC	DX2	130-092-417	Miltenyi Biotec	1:50
CD137	APC	REA765	130-110-764	Miltenyi Biotec	1:50
CD3	APC-Vio770	REA613	130-113-136	Miltenyi Biotec	1:400
CD8	APC-Vio770	REA734	130-110-681	Miltenyi Biotec	1:200
CD4	APC-Vio770	VIT4	130-113-773	Miltenyi Biotec	1:200
CD56	BV421	HCD56	318328	Biolegend	1:100
CD80	BV421	2D10	305222	Biolegend	1:100
CD137	BV421	4B4-1	309820	Biolegend	1:20
PD-1	BV421	EH12.2H7	329920	Biolegend	1:50
TIM-3	BV421	F38-2E2	345007	Biolegend	1:100
c-myc	FITC	14D3	130-092-472	Miltenyi Biotec	1:200
Anti-His	FITC	GG11-8F3.5.1	130-123-633	Miltenyi Biotec	1:200

CD3	FITC	REA613	130-113-138	Miltenyi Biotec	1:400
CD28	FITC	CD28.2	302906	Biolegend	1:100
TNFα	Pacific Blue	MAb11	502920	Biolegend	1:100
CD19	PE	LT19	130-113-169	Miltenyi Biotec	1:50
CD25	PE	M-A251	356104	Biolegend	1:40
CD56	PE	REA196	170-078-057	Miltenyi Biotec	1:200
IFNγ	PE	25723.11	340452	Becton, Dickinson and Company	1:5
LAG-3	PE	11C3C65	369305	Biolegend	1:100
OX40	PE	Ber-ACT35	350003	Biolegend	1:20
TIGIT	PE	MBSA44	12-9500-41	Thermo Fisher Scientific	1:20
CD3	PE-Vio770	REA613	130-113-140	Miltenyi Biotec	1:50
CD19	PE-Vio770	REA675	130-113-647	Miltenyi Biotec	1:200
CD69	PE-Vio770	REA824	130-112-615	Miltenyi Biotec	1:100
Viability dye	Propidium iodide	-	130-093-233	Miltenyi Biotec	1:100
CD4	VioGreen	REA623	130-113-230	Miltenyi Biotec	1:100
CD160	AF488	BY55	53-1609-41	Thermo Fisher Scientific	1:20
2B4	APC	C1.7	329511	Biolegend	1:50
HVEM	APC	122	318808	Biolegend	1:20
CD27	APC	M-T271	561400	Becton, Dickinson and Company	1:100
CTLA-4	APC	BNI3	555855	Becton, Dickinson and Company	1:5
CD7	APC-eFluor780	eBio124-1D1	47-0079-42	Thermo Fisher Scientific	1:100
CD40	BB515	5C3	565258	Becton, Dickinson and Company	1:50
TIM-3	BB515	7D3	565568	Becton, Dickinson and Company	1:100

TIM3	BV421	F38-2E2	345007	Biolegend	1:100
GITR	BV421	108-17	371208	Biolegend	1:20
TIGIT	BV421	A15153G	372709	Biolegend	1:50
OX40	BV421	Ber-ACT35	350013	Biolegend	1:20
Granzyme B	BV421	A18A28	396414	Biolegend	1:100
CD45	BV786	HI30 (RUO)	563716	Becton, Dickinson and Company	1:100
PD-L1	PE	29E.2A3	329705	Biolegend	1:100
CD112	PE	TX31	337410	Biolegend	1:100
CD127	PE	HIL-7R-M21	557938	Becton, Dickinson and Company	1:20
Granzyme B	PE	QA16A02	372208	Biolegend	1:100
myc	AF647	9E10	626810	Biolegend	1:100

Supplementary Table 2: Functional T cell surface molecules measured on T-ALL samples

CD70
ICOS
PD-1
CD28
CD112
CD127
OX40
intracellular PD-L1
surface PD-L1
CD73
CD27
CD25
LAG-3
intracellular CTLA-4
HVEM
CD86
2B4
BTLA
CD40
CD39
GITR
CD57
TIM-3
TIGIT
CD160

Supplementary Table 3: Expression of CD28 on T-ALL blasts and physiologic lymphoid progenitors. Purple samples are derived from patients with T-ALL (n=48), blue samples from healthy controls (n=15, left column), CD28 geometric mean fluorescence intensity (gMFI) is represented in the second column and CD28 positivity as percentage of all $CD7^{+}$ $CD45^{dim}$ $sCD3^{-}$ viable single cells depicted in the third column. Samples were ordered by the percentage of CD28-positive cells and color-coded by sample origin.

sample ID	CD28 MFI	CD28 percent
B615	10371	99,5
B301	1361	99,2
B004	1831	99,1
B006	914	98,9
B623	12490	98,7
B605	10414	98,5
B303	1097	98,3
B002	1506	98,2
B618	5131	98,2
B608	15502	97,8
B616	4155	97,7
B631	9812	97,6
B602	8230	97,5
B627	7193	97,4
B005	953	96,8
B628	4593	96,7
B009	856	95,3
B401	1152	95,3
B606	4238	95,2
B405	1165	93,5
B633	4402	93,5
B010	840	93,1
B626	2797	93
B634	3930	93
B614	3077	91
B613	4828	90,2
B007	795	89,2
B601	4746	89
B603	7509	88,3
B632	2717	80,4
B302	849	78,7
B604	4966	76,1
B622	2261	70,9
B402	366	45,6
B008	326	43,1
B630	569	22,9
B620	555	20,8
B617	418	11,3
B108	99,3	6,81
B502	84,9	5,61
B115	78,7	4,53
B110	73,9	3,69
B113	94,9	3,64
B111	75,7	3,48
B102	73,8	3,19
B114	73,6	2,96
B116	73,4	2,84
B107	59,9	2,8
B103	75,1	2,67
B112	81,2	2,33
B629	192	2,22
B003	63,5	2,08
B109	68,4	2
B101	87,7	1,95
B011	67,1	1,79
B304	69,8	1,53
B016	61	1,36
B117	65,7	1,23
B013	60,2	0,98
B305	76	0,36
B015	36,1	0,22
B014	31,3	0,17
B012	72,2	0,1

Supplementary Table 4: Spectral flow cytometry panel designed according to OMIP71.[1]

Antigen	Dye	Order number	Manufacturer	Dilution
CD3	BUV805	368-0037-41	Thermo Fisher Scientific	1:100
CD4	PerCP-Vio 700	130-113-790	Miltenyi	1:100
CD7	BUV563	753772	BD Biosciences	1:100
CD8a	BUV737	612755	BD Biosciences	1:100
CD25	BV480	566160/566102	BD Biosciences	1:20
CD28	BV711	302948	BioLegend	1:100
CD45	APC-Vio770	130-110-773	Miltenyi	1:50
CD45RO	SB645	64-0457-41, 64-0457-42	Thermo Fisher Scientific	1:100
CD56	BUV661	376-0566-41/ 376-0566-42	Thermo Fisher Scientific	1:100
CD57	BV421	393325	BioLegend	1:100
CD95	APC-Fire810	305663	BioLegend	1:100
CD103	PE-Fire810	350251	BioLegend	1:100
CD127	NovaFluor Blue 660 / 120S	H017T02B08-N	Thermo Fisher Scientific	1:100
CD183 (CXCR3)	PE	130-120-592	Miltenyi	1:50
CD184 (CXCR4)	BUV395	563924	BD Biosciences	1:100
CD185 (CXCR5)	RB613	758534	BD Biosciences	1:100
CD194	PE-Fire700	359435	BioLegend	1:100
CCR6 CD196	RY586	753568	BD Biosciences	1:100
CD197 (CCR7)	BV785	353229	BioLegend	1:100
CD279 (PD-1)	AF647	367419	BioLegend	1:100
HLA DR	cFluorB515	R7-20102	Cytek Biosciences	1:100
TCR gamma/delta	R718	752023	BD Biosciences	1:100
TCR V alpha 24 J alpha 18	RB744	757400	BD Biosciences	1:100
Superbright buffer		SB-4401-42	Thermo Fisher Scientific	1:100

Supplementary Figure 1

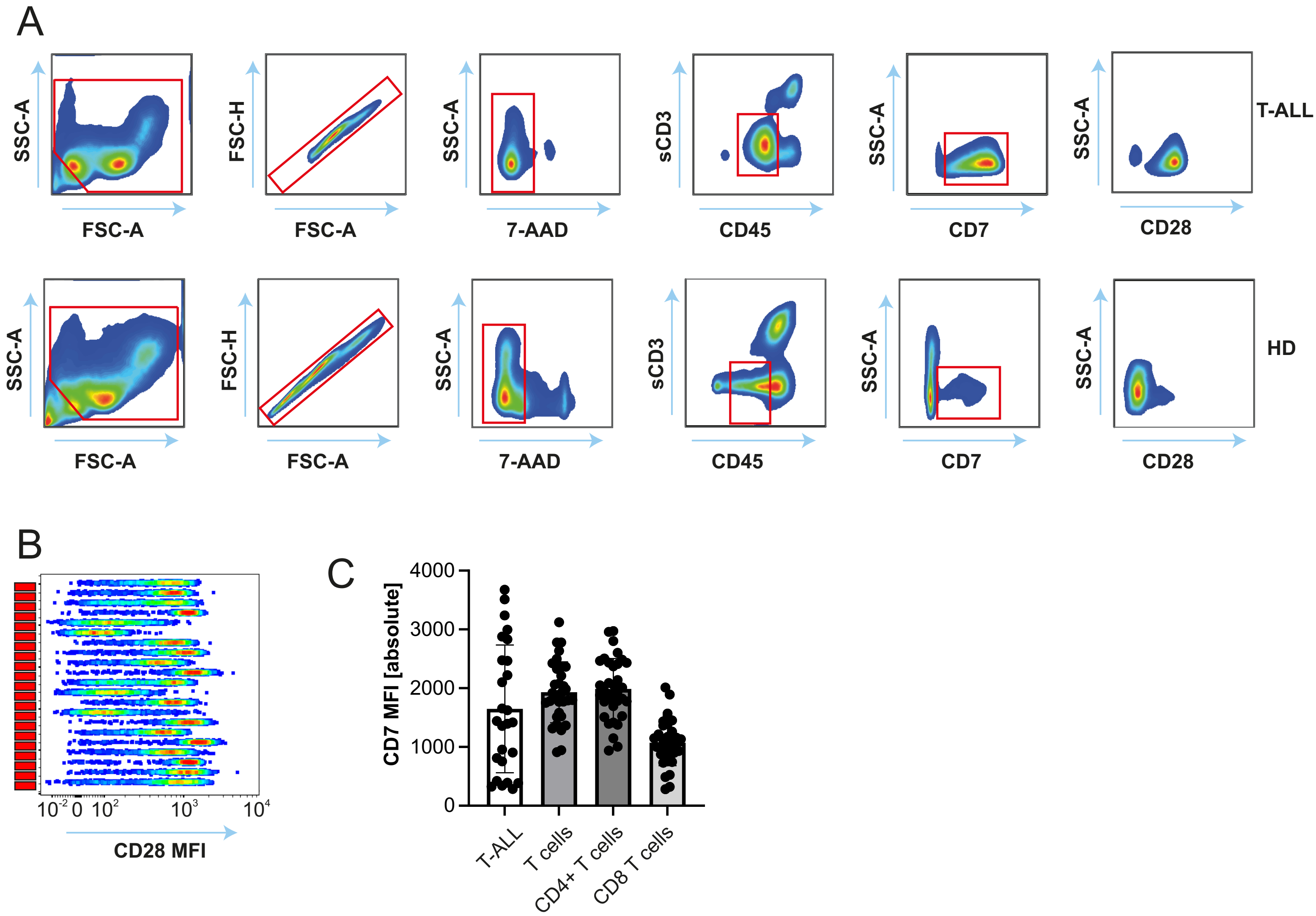

Supplementary Figure 1, CD28 and CD7 surface expression in T-ALL blasts and physiologic lymphoid populations. A: Gating strategy employed for identification of T-ALL blasts in patients with T-ALL (upper panels) and lymphoid progenitors from healthy bone marrow donors (lower panels). **B:** A second set of T-ALL samples analyzed using an alternative anti-CD28 antibody (BV650) and are therefore not compared to healthy lymphoid progenitors in terms of MFI (n=21). **C:** CD7 expression by MFI in T-ALL blasts (n=26), physiological total T cells and $CD4^+$ and $CD8^+$ T-cell subsets (n=34) illustrates the highest mean CD7 MFI in CD4 T cells with T-ALL blasts showing a greater inter-sample variability in surface CD7 expression. Source data are provided as a Source Data file.

Supplementary Figure 2

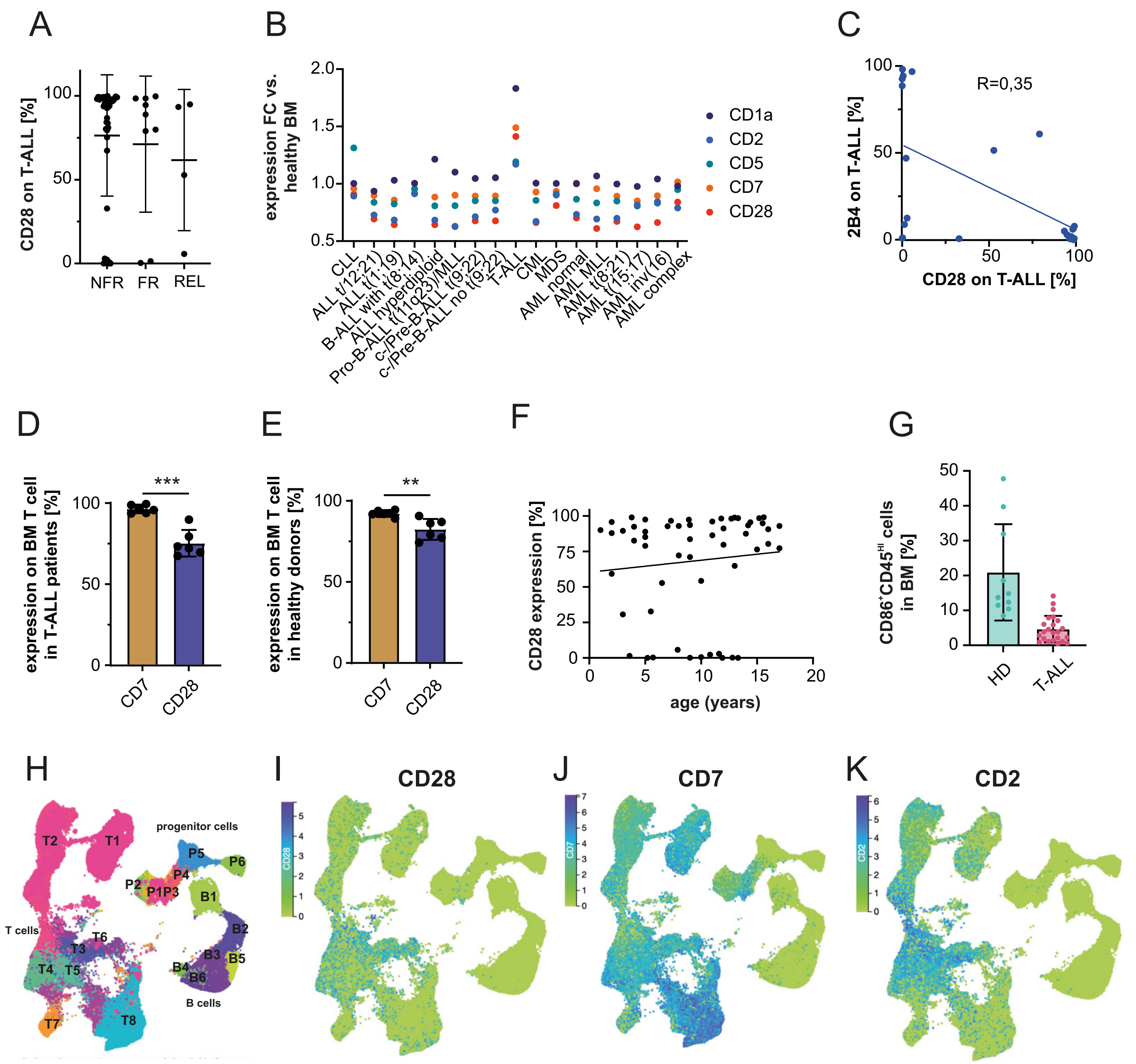

Supplementary Figure 2, CD28 expression across T-ALL patient samples and physiologic haematopoietic and T-cell development. A: No difference in CD28 surface expression on T-ALL patient samples was observed on patients with primary disease who did not subsequently relapse (NFR, n=42), who subsequently relapsed (FR, n=9) or in samples of matched relapsed patients (REL, n=4). **B:** RNA expression of currently known T-ALL target antigens in hematologic diseases relative to healthy bone marrow by microarray (T-ALL: n=174, total samples: n=2113).[2] **C:** Inverse correlation of 2B4 expression and CD28 expression on T-ALL blasts from patient samples (n=52, linear regression: R^2=0.35, p=0.0023). **D&E**: CD7 and CD28 surface expression in both healthy bone marrow (D, n=6) and T-ALL leukemic bone marrow (E, n=6) physiologic T cell populations. CD7 expression was higher than CD28 expression in both populations (92% vs. 82% in healthy donors, 96% vs. 75% in patients with T-ALL, unpaired two-tailed t tests, p=0.0001 and p=0.048). **F:** CD28 expression on the surface of T-ALL (n=56) was independent of patient age. **G:** Frequency of CD86+ events within the bone marrow of patients with T-ALL (n=23) was reduced when compared with healthy donors (n=10, 5% vs. 21%, unpaired two-tailed t test, p=0.0046). **H:** Re-analysis of single-cell sequencing data.[3] Annotation of populations depicted: P1: Hematopoietic stem cell, P2: megakaryocyte progenitor, P3: early lymphoid progenitor, P4: pre-pro B-cell, P5: pro-B cell, P6:late pro-B cell; B1: large pre-B-II cell, B2: small pre-B-II cell, B3: B-2-B cell, B4: mature B cell, B5: immature B cell, B6: B-1B cell; T1: DN thymocyte, T2: DP thymocyte, T3: regulatory T cell, T4: naive CD4 SP T cell, T5: naive CD8 SP T cell, T6: CD8 intraepithelial T cell, T7: innate lymphoid cell T8: NK cell. Data were derived from 25 embryos/fetuses, comprising a total of 908,178 analyzed cells. **I-K:** illustration of CD28 (I), CD7 (J) and CD2 (K) expression during T-cell development. Source data are provided as a Source Data file.

Supplementary Figure 3

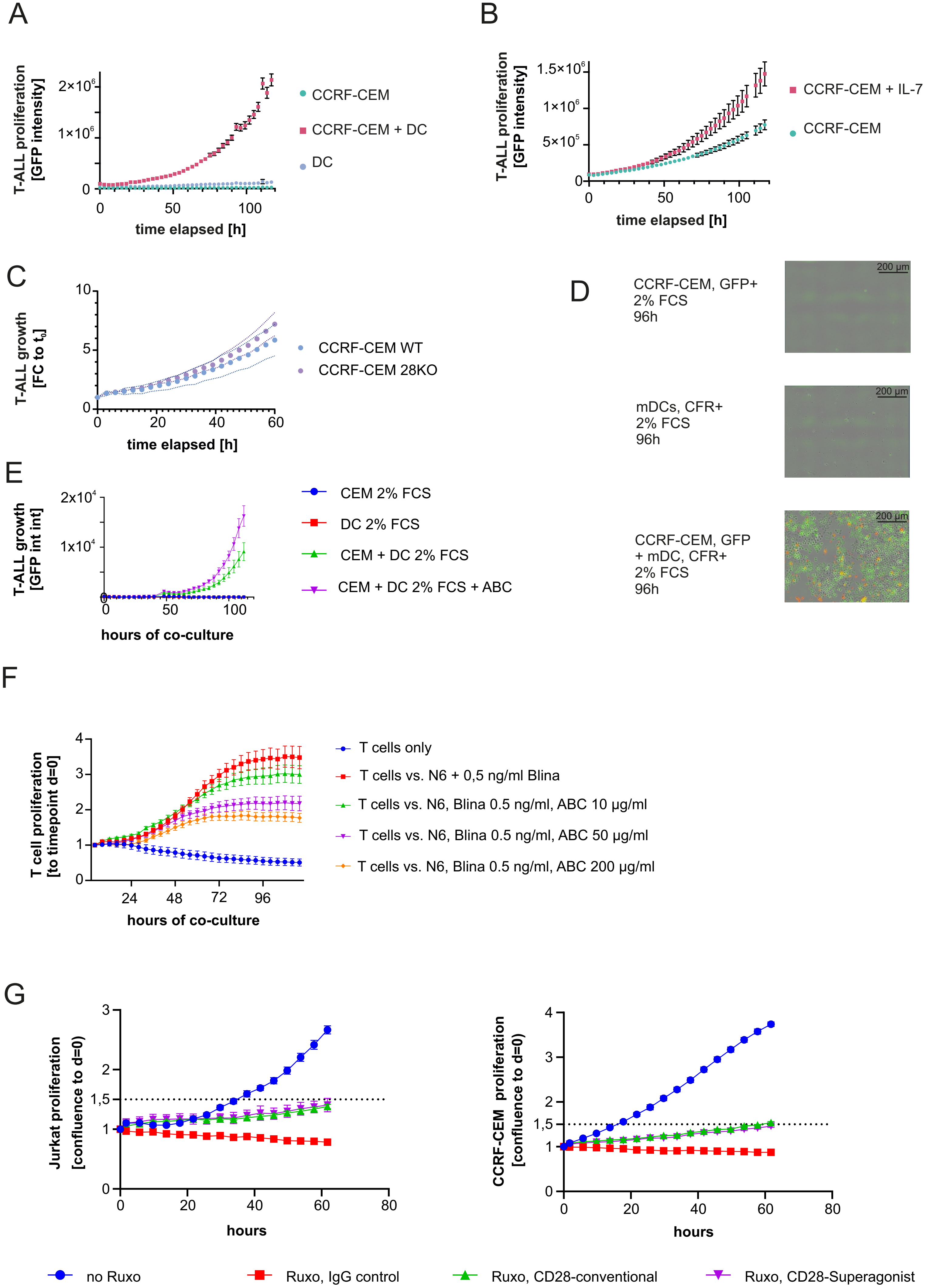

Supplementary Figure 3, Modulation of CCRF-CEM proliferation by dendritic cells, cytokine signalling and CD28 pathway perturbation. A: CCRF-CEM cells were cultured alone or with monocyte-derived dendritic cells (moDCs) in medium containing 2% FCS. CCRF-CEM growth increased significantly in the presence of moDCs (96h: Mann Whitney test, p=0.0286, four technical replicates). **B:** CCRF-CEM cells were cultured in 5µM dexamethasone either in the absence or in presence of IL-7 (125 ng/ml). IL-7 significantly increased T-ALL proliferation (120h: Mann Whitney test, p=0.0286, four technical replicates). **C:** Growth of WT CCRF-CEM and CD28 KO CCRF-CEM cells over 60 hours with no difference in cell growth. Three technical replicates. **D:** Representative images acquired by Incucyte SX5 illustrate CCRF-CEM proliferation induced by moDCs. Images representative of three technical replicates. **E:** Addition of abatacept (Bristol-Myers Squibb, 200 ug/ml) did not abolish CCRF-CEM proliferation induced by moDC co-culture. GFP integrated intensity, three technical replicates. **F:** Calibration of abatacept-sensitive assay: T cell and GFP^+ $CD80^+CD19^+$ BCP-ALL cell line Nalm6 were co-cultured and T-cell proliferation was monitored using the Cell-by-Cell analysis module of the Incucyte SX5. Co-Culture of T cells and Nalm6 in the presence of Blinatumomab (500 ng/ml) induced T-cell proliferation (left panel). Increasing abatacept doses led to a dose-dependent decrease of T-cell proliferation indicating reduced T cell activation by CD28 ligation. Three technical replicates. **G:** Inhibition of Jurkat and CCRF-CEM proliferation by addition of 25 µM Ruxolitinib (MedChemExpres, HY-50856) was in part reverted by addition of either conventional CD28.2 antibody (Cat-Nr. 302934, Biolegend) or superagonistic S20013B CD28 antibody (Cat-Nr. 377804, Biolegend). Three technical replicates. Source data are provided as a Source Data file.

Supplementary Figure 4

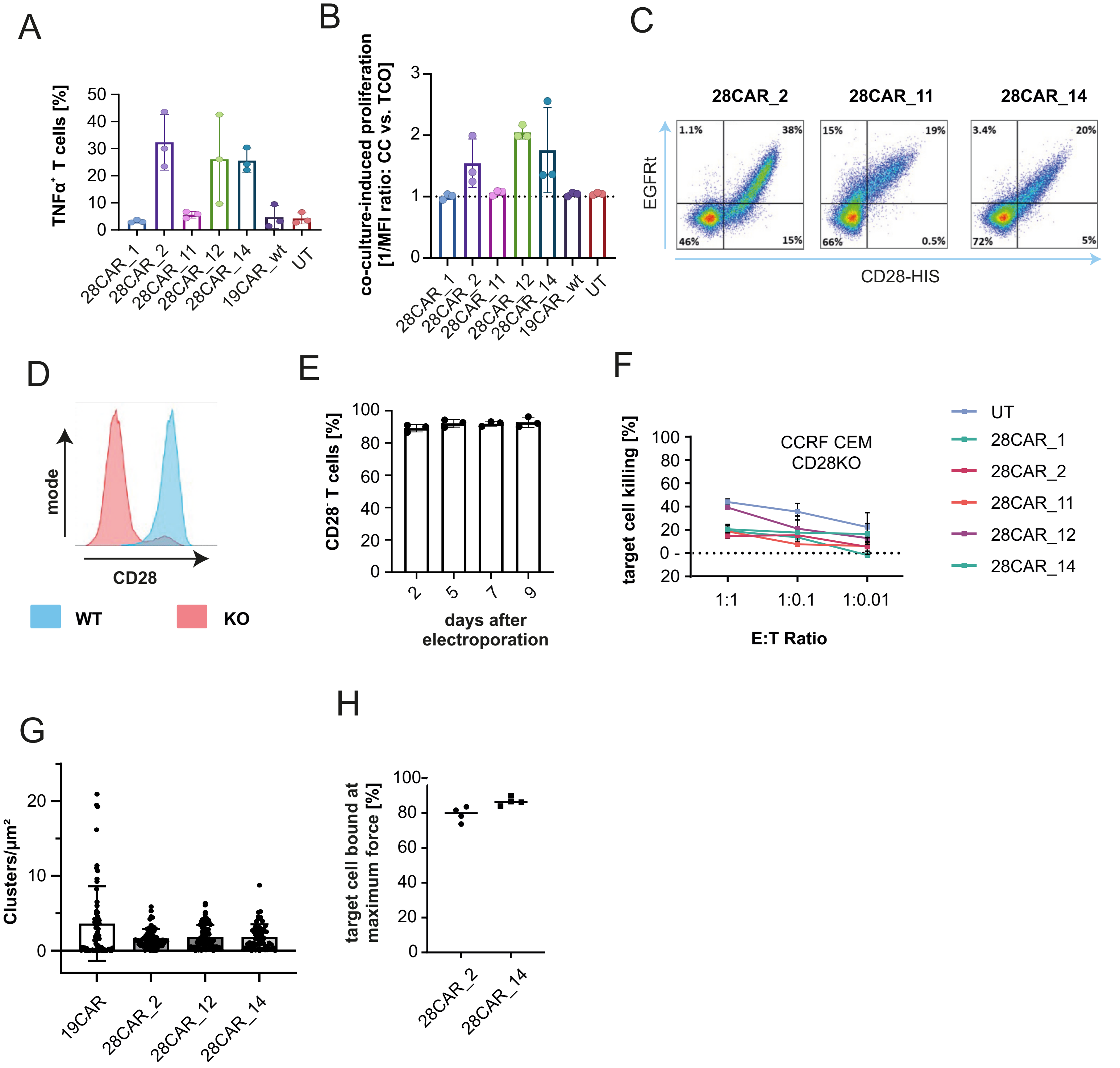

Supplementary Figure 4, Functional validation, surface expression and avidity of anti-CD28 CAR constructs. A: Tumor necrosis factor alpha (TNFα) release measured by intracellular cytokine staining 4h after addition of Brefeldin-A to a co-culture of 100,000 CAR T cells and 100.000 GFP^+ CCRF-CEM cells. Mean values: 28CAR_1: 3%, 28CAR_2: 32%, 28CAR_11: 6%, 28CAR_12: 26%, 28CAR_14: 26%, 19CAR: 5%, UT: 4% (Friedman test, p=0.0241, three independent donors). **B:** Co-Culture induced proliferation defined as inverse MFI ratio (co-culture (CC) / T cell only (TCO) shows reduction of Cell Trace Violet (CTV) MFI in 28CAR_2, 28CAR_12 and 28CAR_14 T cells (Friedman test, p=0.0252, three independent donors). **C**: Use of EGFRt and sandwich protein staining using recombinant CD28 with HIS tag and subsequent anti-HIS staining prior to flow cytometry analysis. The three anti-CD28 CAR molecules illustrate different biological features with CAR surface expression exceeding EGFRt expression (28CAR_2, left panel), equal amounts of CAR and EGFRt surface expression (28CAR_14, right panel) and EGFRt expression exceeding CAR surface expression (28CAR_11). Data are representative of two donors. **D:** Histograms illustrating representative flow cytometry data on CD28 KO by CRISPR/Cas9. Data representative for three donors. **E:** Frequency of $CD28^-$ T cells after CD28 KO by CRISPR/Cas9 remains stable over time. Three independent donors. **F:** Co-culture of anti-CD28 CAR T cells with CD28 KO Jurkat T cells. No specific killing above the background of non-transduced T cells (UT) was observed. Three individual donors. **G:** CAR cluster detected by FITC-CAR staining and subsequent analysis by dSTORM microscopy illustrates cluster density in 28CAR_2, _12 and _14 (~2 / square μm^2) compared to CD19 CAR T cells (n=297 cells analyzed). Total of 312 cells analyzed. **H**: At maximum force (1000 pN), more Jurkat cells were bound to $CD28^+$ target cells when transduced with 28CAR_14 compared to 28CAR_2 (median 86.6% vs. 82.2%, Mann Whitney test, p=0.0286). Four biological replicates. Source data are provided as a Source Data file.

Supplementary Figure 5

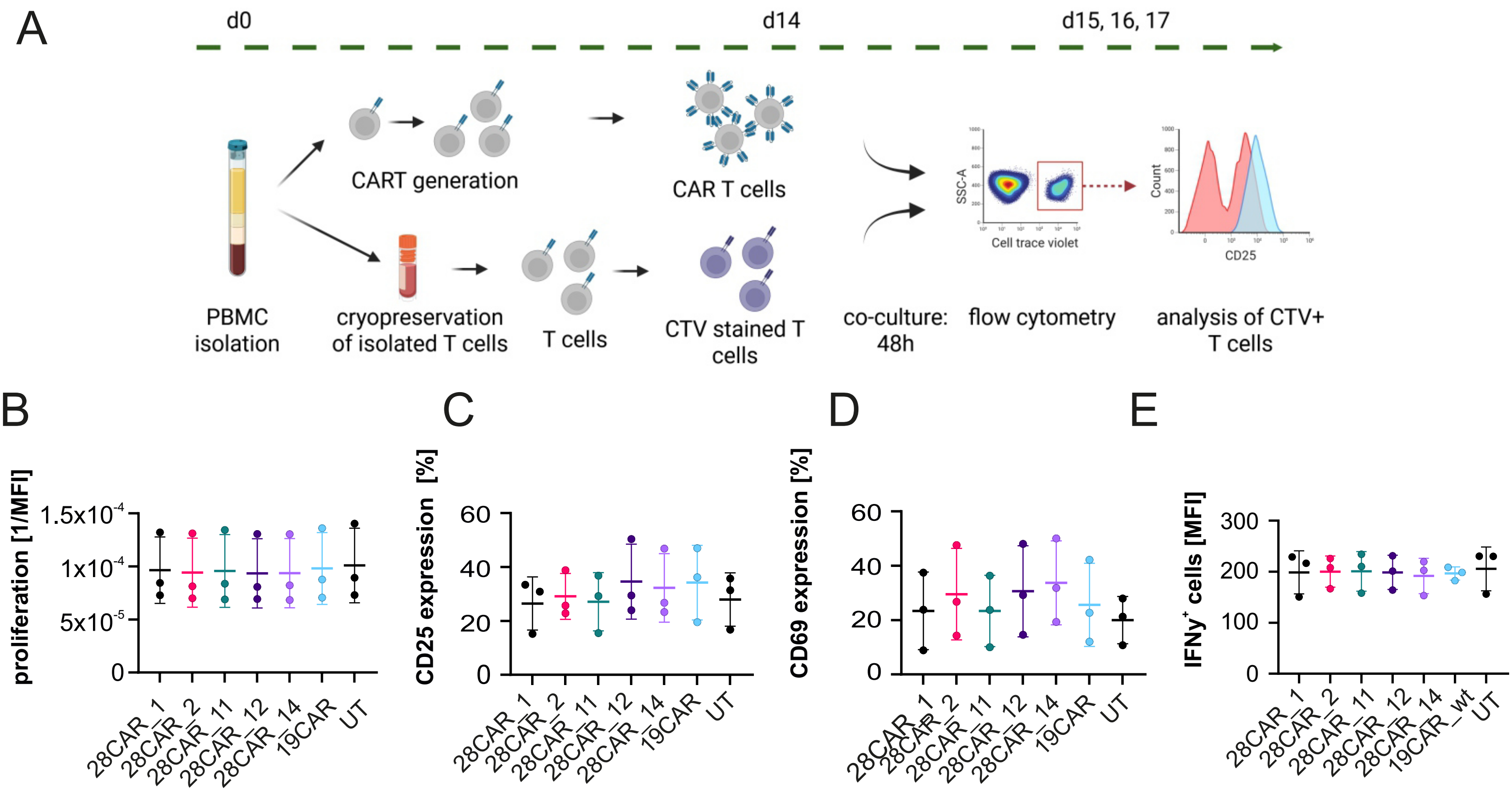

Supplementary Figure 5, Anti-CD28 CAR T cells do not induce activation or proliferation of autologous wild-type T cells. A: Non-transduced T cells were labelled with CTV and co-cultured with anti-CD28 CAR T cells to assess activation of wild-type T cells by ligation of CD28 by CAR T cells. Created in BioRender. Burk, U. (2026) https://BioRender.com/wqxsdqv. **B:** Proliferation as measured as 1/MFI of CTV, no alteration of CTV-labelled, non-transduced T-cell proliferation was observed after co-culture with five functional anti-CD28 CAR T cells when compared to anti-CD19 CAR T cells or non-transduced T cells (UT). **C:** In the same experimental setup, CD25 and CD69 surface expression was assessed as correlates of T cell activation 48 hours after co-culture. Again, no difference in T cell activation could be observed. **D&E:** Surface expression of CD69 (D) and intracellular IFNγ (E) was determined after 48 h of co-culture of CAR T cells with autologous T cells and, once more, no signs of increased activation of T cells by binding of anti-CD28 CAR T cells could be observed. Three individual donors with technical duplicates (B-E). Source data are provided as a Source Data file.

Supplementary Figure 6

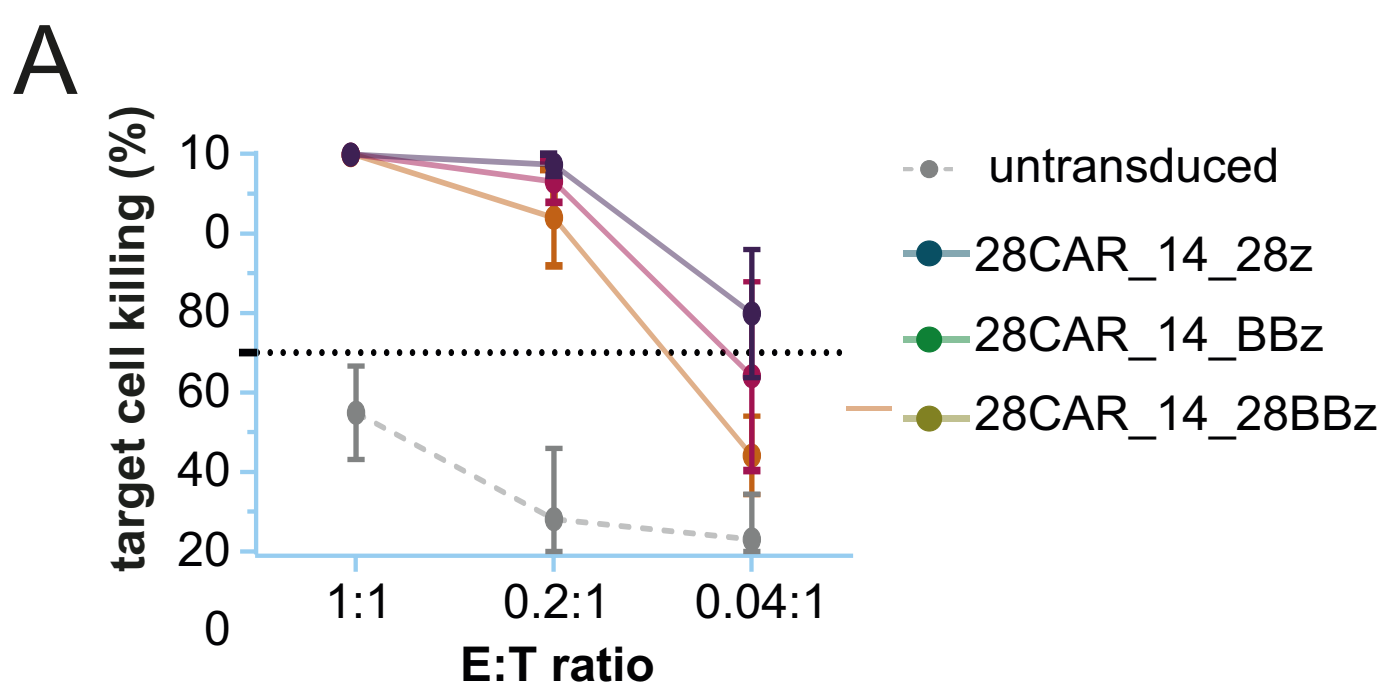

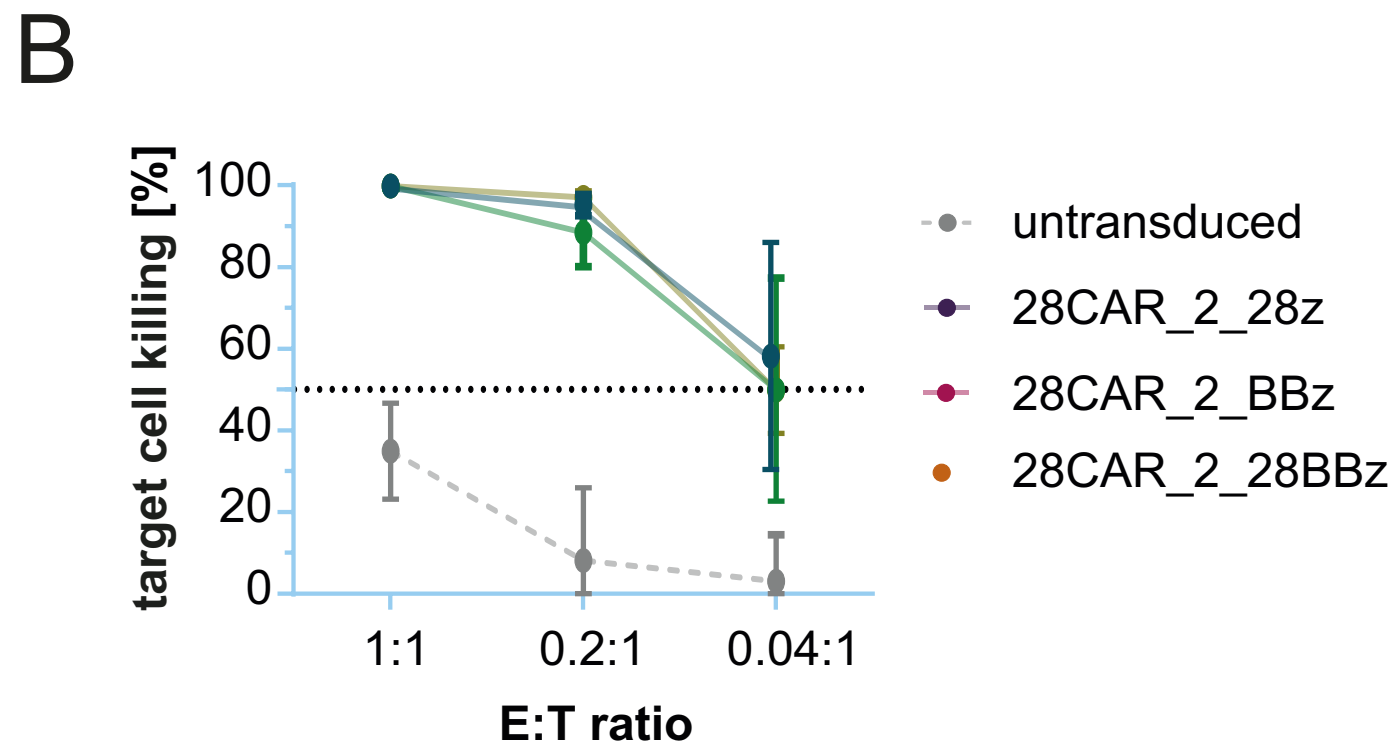

Supplementary Figure 6, Costimulatory-domain-dependent cytotoxicity of 28CAR_14, but not 28CAR_2, against Jurkat T-ALL cells. A: Cytotoxicity of 28CAR_14 T cells harboring different costimulatory domains against Jurkat cells at three given E:T ratios. Only at an E:T ratio of 0.04:1, cytotoxicity of 28CAR_14 T cells harboring the CD28 costimulatory domain was significantly higher than cytotoxicity of 28CAR_14 T cells harboring 41BB or 28BB costimulatory domains (60% vs. 44% and 24%, Friedman test, p=0.002). **B:** 28CAR_2 T cells did not show altered cytotoxicity after substitution of the CD28 costimulatory domain by 41BB or 28BB costimulatory domain at any E:T ratio. Three individual donors with technical duplicates (A&B). Source data are provided as a Source Data file.

Supplementary Figure 7

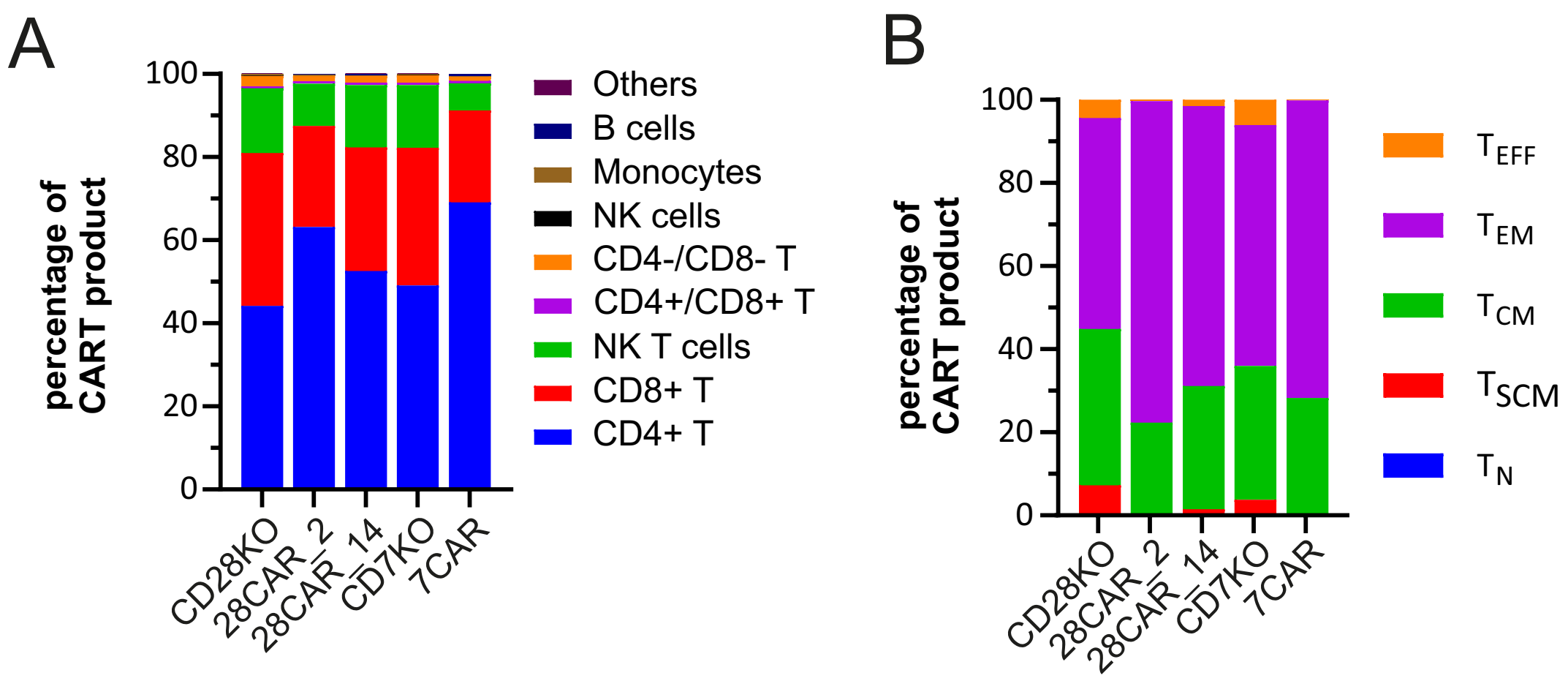

Supplementary Figure 7, Cellular composition and maturation of CD7- and CD28-knockout CAR T cell products after in vitro expansion. A: Cellular composition of CAR T cell products or CD7 KO and CD28 KO after 14 days of in vitro expansion in the presence of 12.5 ng/ml IL-7 and IL-15. **B**: Maturation of CAR T cell products after 14 days of in vitro expansion. Four individual donors (A&B). Source data are provided as a Source Data file.

Supplementary Figure 8

A

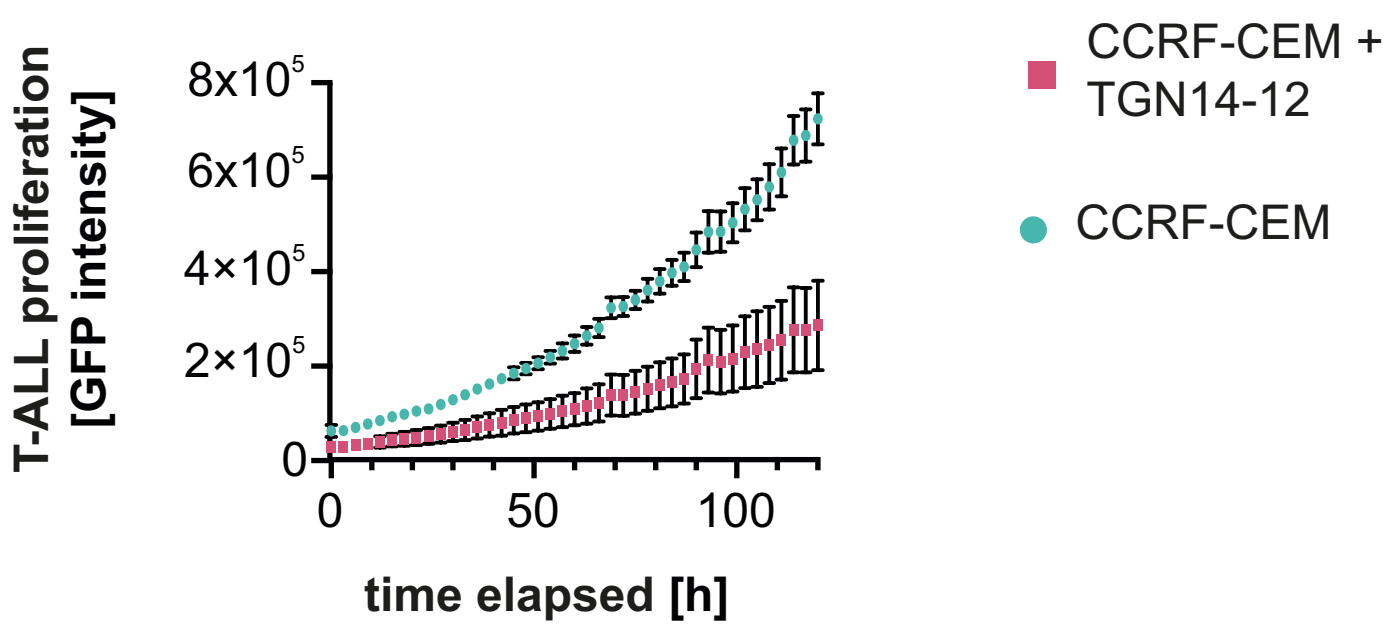

B

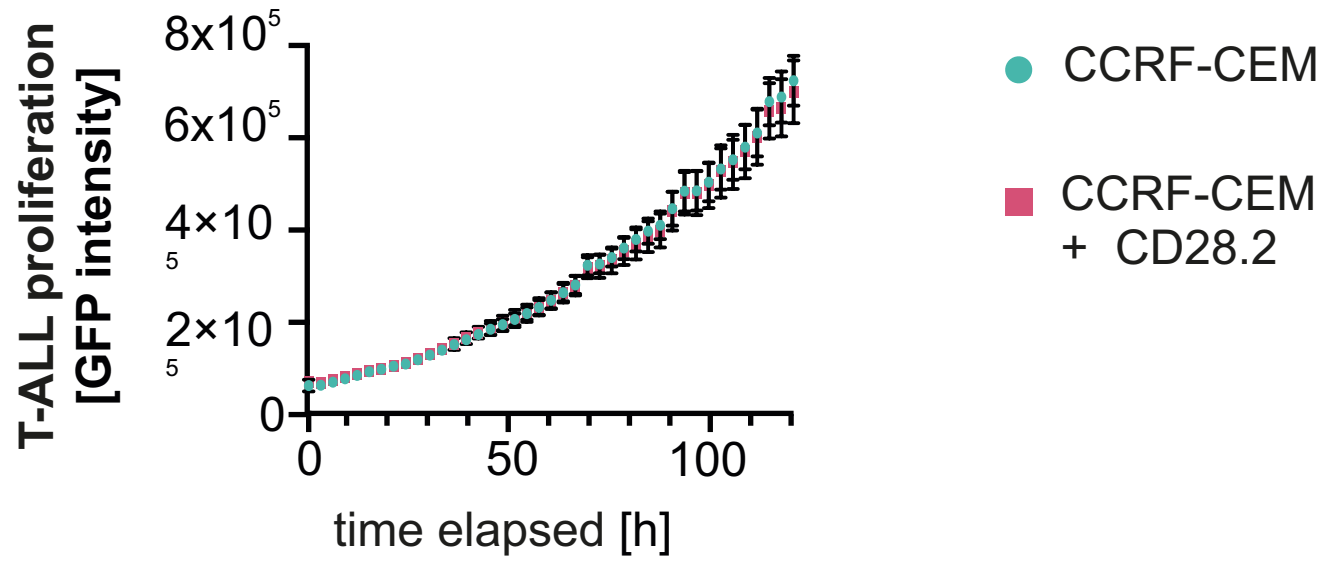

Supplementary Figure 8, TGN1412, but not conventional CD28 ligation, inhibits CCRF-CEM proliferation under cytarabine stress conditions. A: CCRF-CEM cells were cultured under stress condition (10 nM Cytarabin) in the presence of TGN1412 (5 µg/ml), which led to decreased proliferation over 120h. **B:** CCRF-CEM cells were cultured under stress condition (10 nM Cytarabin) in the presence of CD28.2 antibody (5 µg/ml) which did not affect the proliferation of T-ALL cells. Four technical replicates (A&B). Source data are provided as a Source Data file.

Supplementary Figure 9

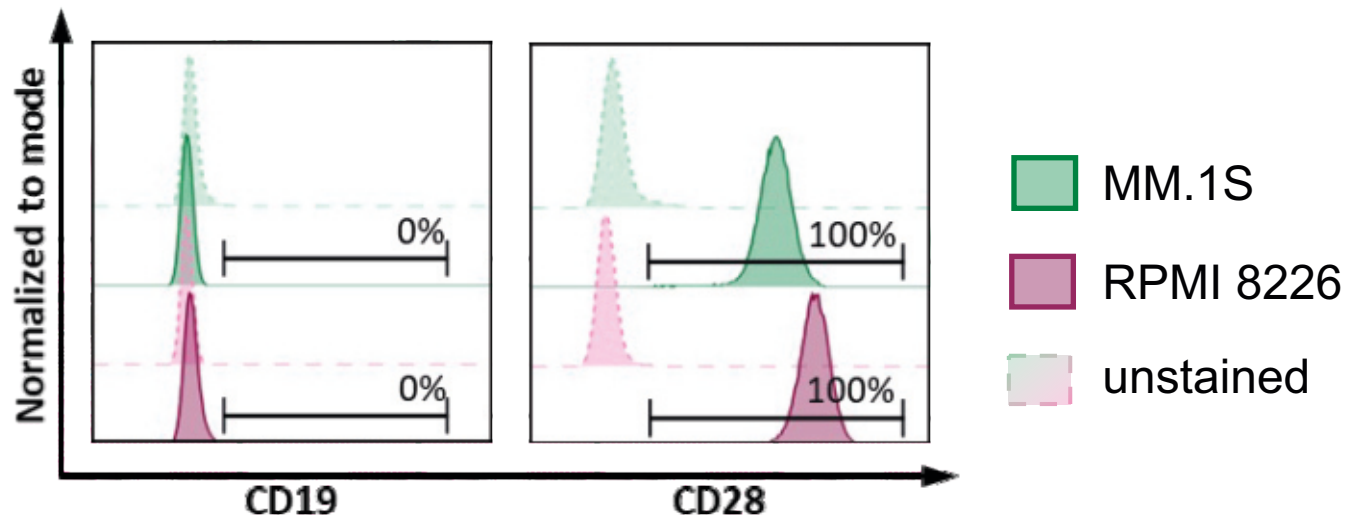

Supplementary Figure 9: Expression of CD19 and CD28 on both MM cell lines used, MM.1S and RPMI 8226. Data representative of two measurements.

Supplementary Figure 10

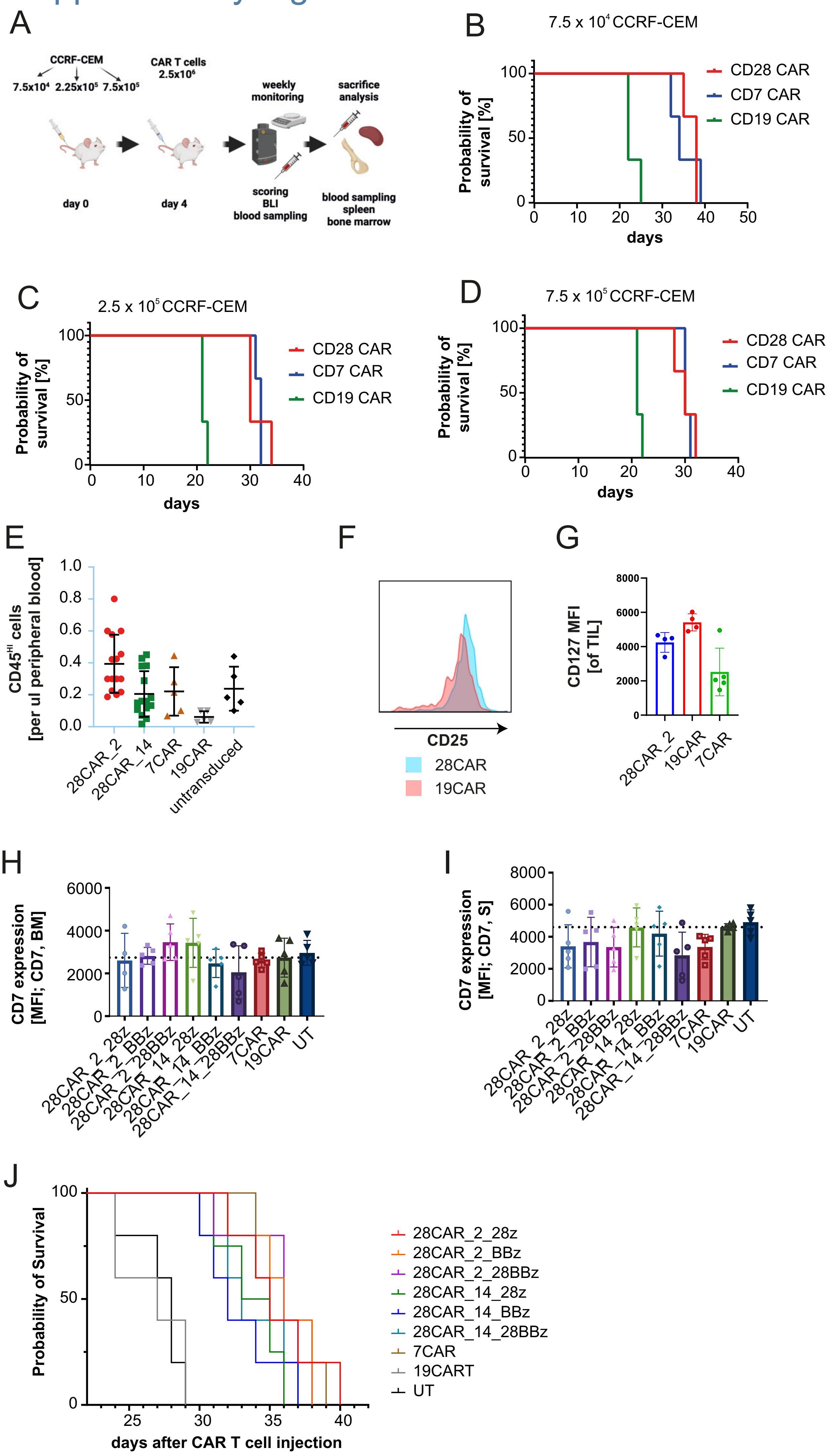

Supplementary Figure 10: T-ALL xenograft dose-finding and in vivo characterization of anti-CD28 and anti-CD7 CAR T cells. A: Schematic of a dose-finding T-ALL xenograft experiment to determine the CCRF-CEM dose. Created in BioRender. Burk, U. (2026) https://BioRender.com/2nrwtnr. **B-D:** Either 7.5 x 10^4 (B), 2.25 x 10^5 (C) or 7.5 x 10^5 (D) CCRF-CEM cells transduced with GFP/fLUC were injected into mice four days prior to CAR T cell injection. Nine mice per CCRF-CEM dose, three mice per treatment group. **E:** Persistence of $CD45^{hi}$ cells in peripheral blood was higher for 28CAR_2 treated mice with a mean of 0.4 cells/µl compared to 0.2 cells/µl in 28CAR_14 treated mice 11 days after injection (Kruskal-Wallis test, p=0.0008, two-tailed Mann Whitney test, p=0.0019; 15 mice with 28CAR_2 and 28CAR_14 CAR treatment each, 5 mice for remaining treatment groups). **F:** Histogram illustrating increased CD25 MFI in spleen T cells harvested from mice treated with 28CAR_2 T cells when compared to 19CAR-treated mice after mouse sacrifice at day 3 after CAR T cell injection. Representative data for four biological replicates. **G:** Illustration of reduced CD127 expression in the same spleen T cells isolated from CAR T cell treated mice three days after injection. Four mice each were treated with 28CAR_2 or 19CAR T cells, and five mice were treated with 7CAR T cells. **H&I**: CD7 surface expression in CCRF-CEM cells harvested from the bone marrow (H) or spleen (I) of mice. Five mice per group, four mice for BM of 28CAR_2-treated mice. The same experiment is described in Figure 4A-E. **J:** Survival of mice injected with CCRF-CEM cells and subsequently with CAR T cells targeting CD28, CD7 or CD19 or untransduced T cells with CAR constructs harboring different costimulatory domains (28z: CD28 costimulatory domain, BBz: 41BB costimulatory domain, 28BBz: both CD28 and 41BB costimulatory domain). This panel presents the data shown in Figure 4E stratified by costimulatory domain. Five mice per group. Source data are provided as a Source Data file.

Supplementary Figure 11

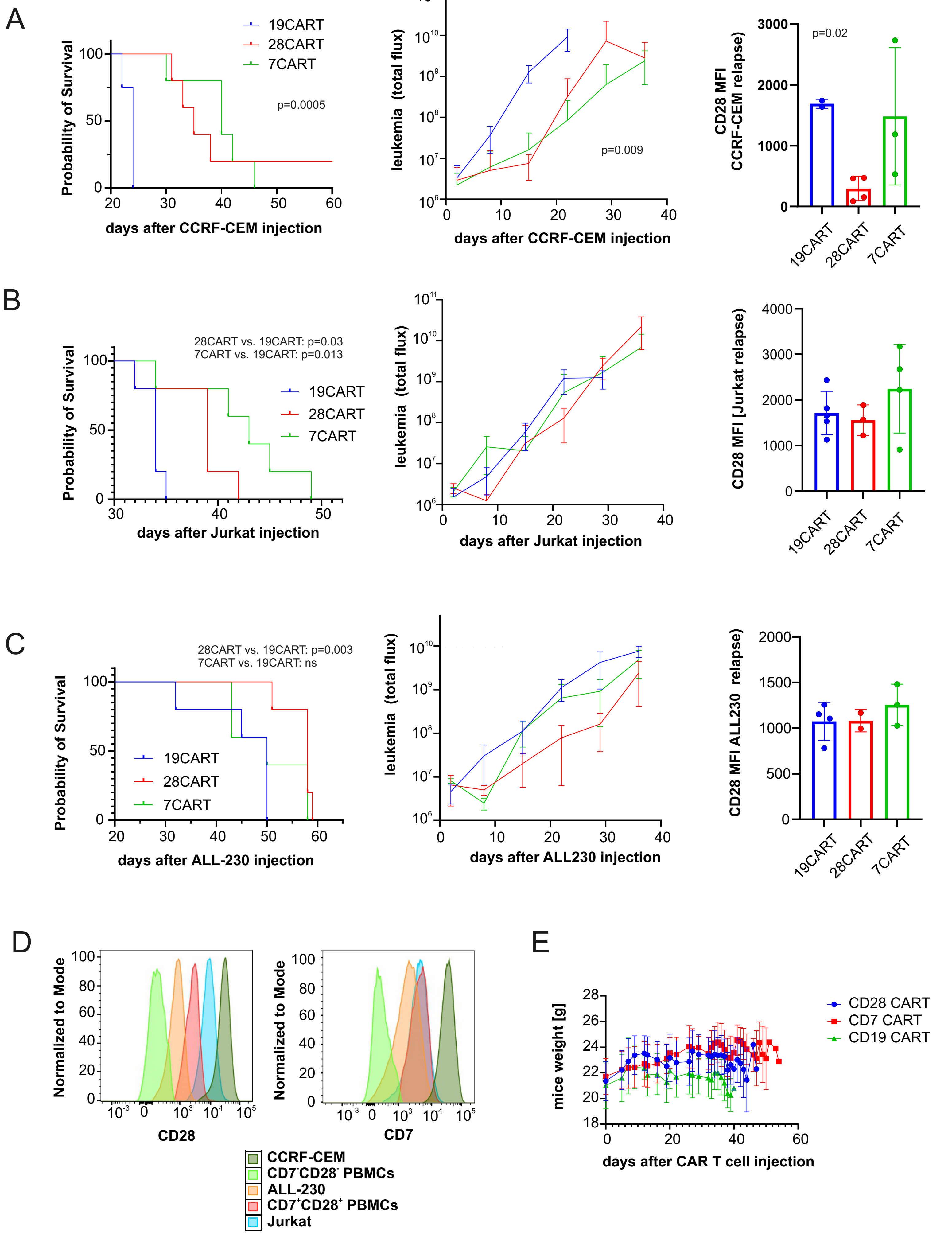

Supplementary Figure 11, A: Mice injected with 1 x 10^6 CCRF-CEM and 4 days later treated with 5 x 10^6 control anti-CD19 CAR T cells or anti-CD7 CAR T cells or anti-CD28 CAR T cells prolonged survival in mice treated with anti-CD28 or anti-CD7 CAR T cells to a comparable extent (left panel, Logrank test, p=0.0005). Leukemia growth was slowed significantly (middle panel, Kruskal-Wallis test at d+14, p=0.009) and CD28 surface expression was significantly reduced on relapsed CCRF-CEM at sacrifice (right panel, Kruskal-Wallis test, p=0.02). The same experiment was performed using Jurkat cells (**B**) or ALL-230 patient-derived-xenograft cells (**C**). In the Jurkat model, anti-CD7 CAR T cells conferred a greater survival benefit than anti-CD28 CAR T cells (B, left panel, anti-CD7 CAR T vs. anti-CD19 CAR T: Logrank test, p=0.013, anti-CD28-CAR T vs. anti-CD19 CAR T: p=0.03) and a stronger effect of anti-CD28 CAR T cells against ALL-230 PDX line (anti-CD7 CAR T vs. anti-CD19 CAR T: ns, anti-CD28 CAR T cell vs. anti-CD19 CAR T cell: p=0.003). CD28 surface expression remained unchanged in both T-ALL mouse models after T-ALL recurrence and sacrifice of mice, when T-ALL cells were harvested from mouse bone marrow. Owing to technical limitations, CD28 surface-expression analysis was available for two mice in the ALL-230/28CAR treatment group and for at least three mice in all other groups. **D**: CD28 (left plot) and CD7 expression by MFI (right plot); CD28 and CD7 expression was highest in CCRF-CEM cells, with CD7 expression being similar on Jurkat, $CD7^+$ human PBMCs and slightly lower on ALL-230 while CD28 expression showed a clear pattern with Jurkat > $CD28^+$ human PBMC > ALL-230. $CD28^-CD7^-$ human PBMCs serve as reference. **E**: Weight of individual mice injected with Jurkat cells is illustrated to contextualize the observed survival difference in mice treated with anti-CD19 CAR T cells when compared to mice treated with either anti-CD7 or anti-CD28 CAR T cells. Mice treated with anti-CD19 CAR T cells after injection of Jurkat cells show earlier weight loss. Three mice per treatment group (A-E). Source data are provided as a Source Data file.

Supplementary Figure 12

A

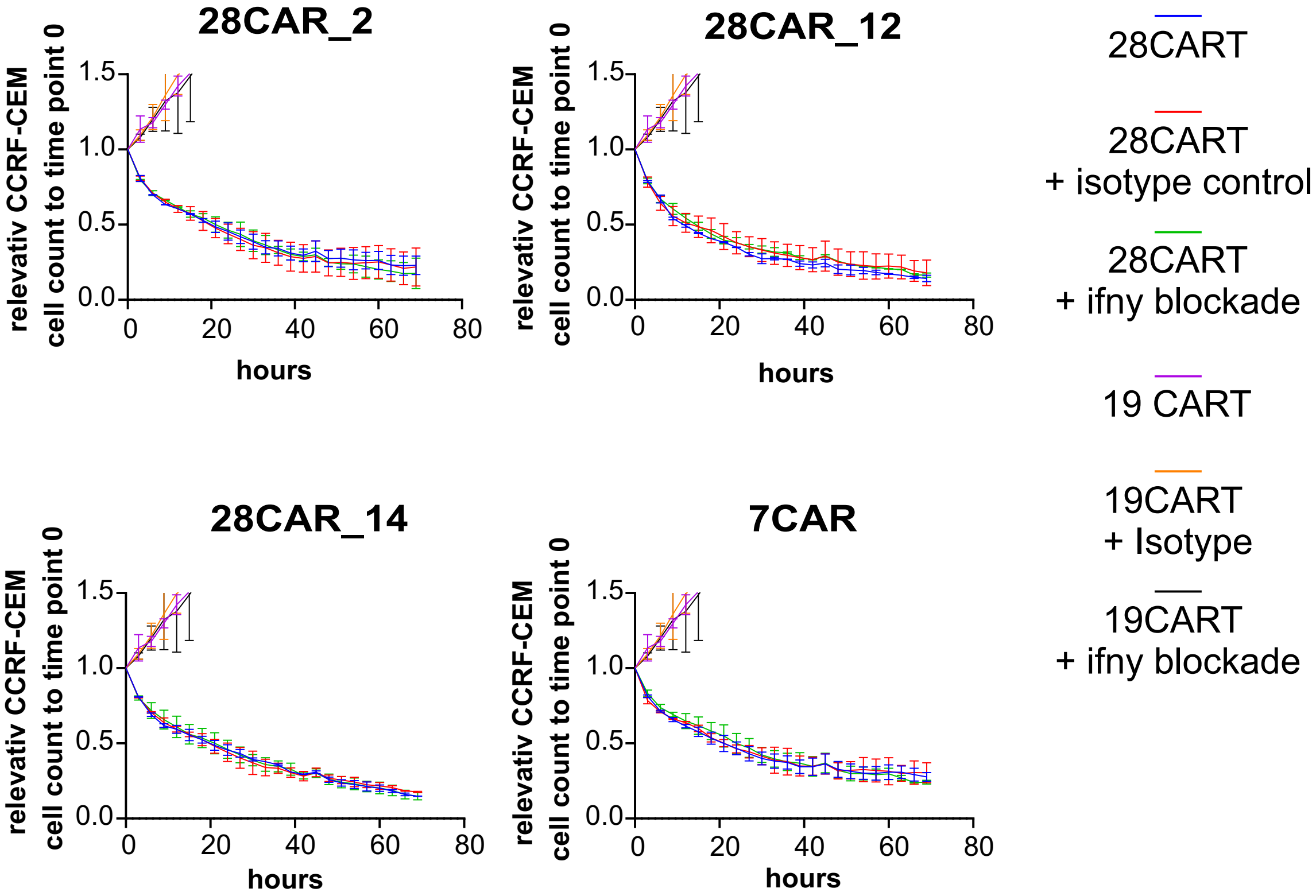

B

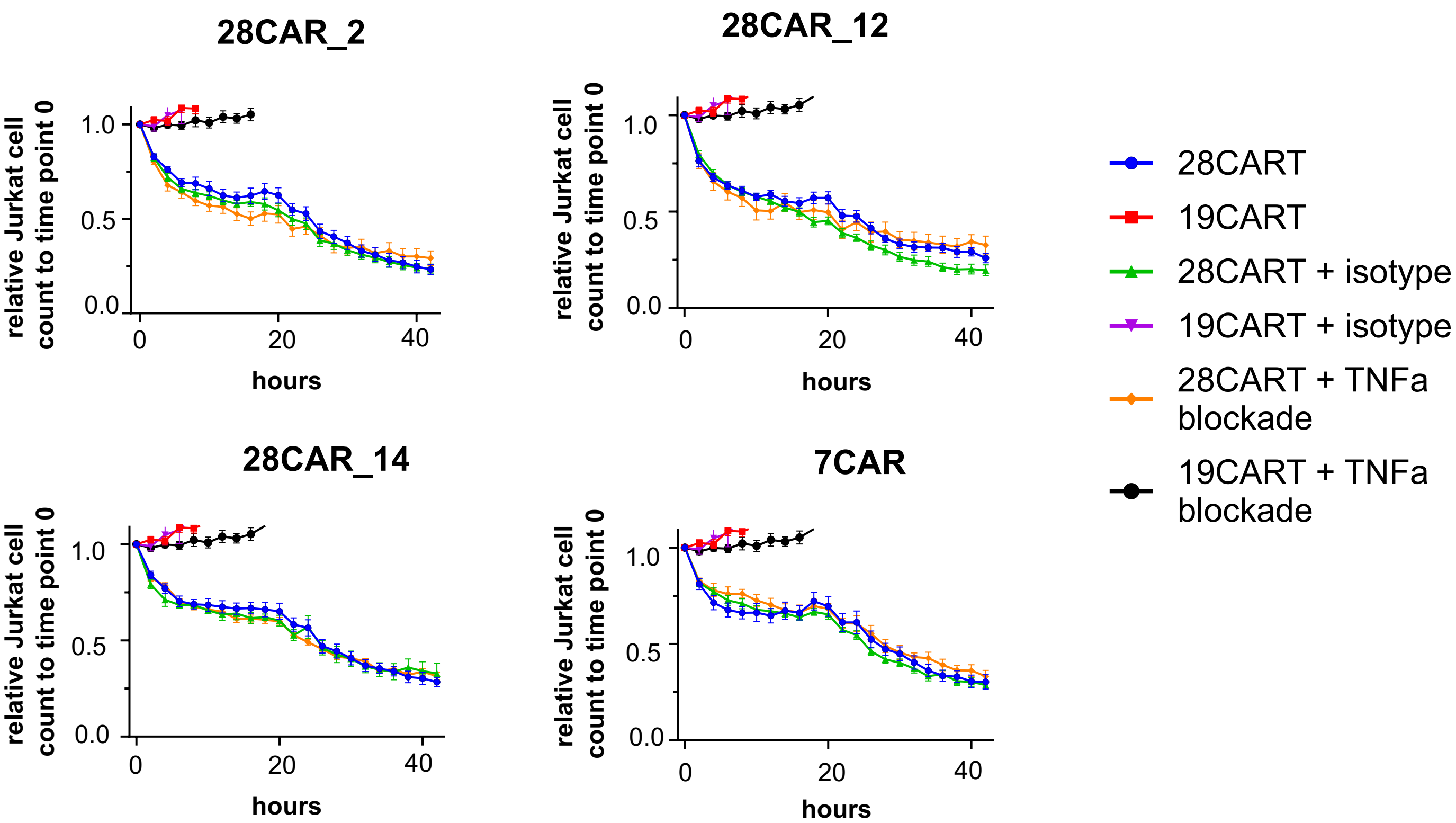

Supplementary Figure 12, A: Four specific anti-CD28 CAR T cells were co-cultured at a 1:1 effector to target ratio with 1 x 10^5 CCRF-CEM cells. In order to test dependency on IFNγ for cell killing, IFNγ-blocking antibody (clone: NIB42, Cat: 14-7318-81, Thermo Fisher Scientific) was added at 1 µg/ml, using an isotype control (clone: MOPC-21, Cat: 400102, Biolegend) at the same concentration. No difference in T-ALL growth was observed when using the Incucyte SX5 for live cell imaging and quantification. **B:** Using a similar experimental design, 3.5 x 10^5 Jurkat T-ALL cells were co-cultured with CAR T cells in a 1:1 effector to target ratio either in the presence of TNFα blocking antibody (clone: MAB2101, Cat: MAB2101-100, R&D Systems) or the above-mentioned isotype control at 10 µg/ml. No difference in T-ALL growth was observed when using the Incucyte SX5 for live cell imaging and quantification. Experiments were performed in technical triplicates, Data are presented as mean +/- s.e.m. from technical triplicates. Source data are provided as a Source Data file.

Supplementary Figure 13

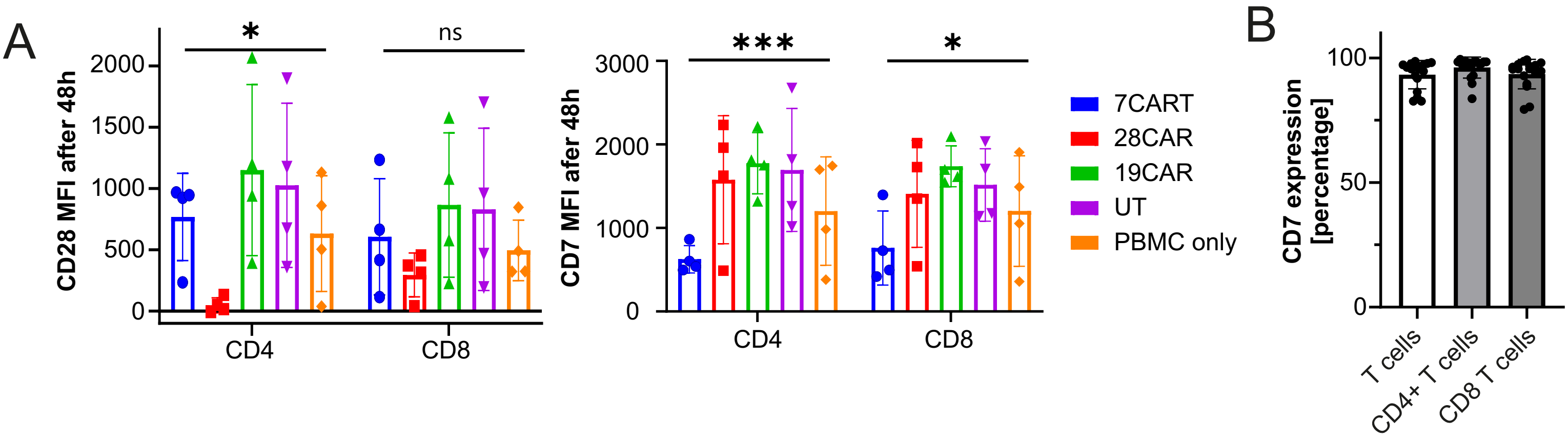

Supplementary Figure 13, A: After co-culture of autologous PBMCs with CAR T cells targeting CD19, CD7 or CD28 or untransduced T cells for 48 hours, cells were subjected to 25-color flow cytometry for analysis of CAR T-cell-induced lymphodepletion. In the left panel, CD28 MFI is depicted after CAR T cell co-culture. CD28 surface expression is almost completely abolished in CD4 T cells (Friedman test, p=0.019), while CD8 T cells retain residual CD28 expression after co-culture with autologous anti-CD28 CAR T cells (Friedman test, p=0.246). The right panel illustrates reduction of CD7 surface expression after co-culture with anti-CD7 CAR T cells (Friedman test, CD4: 0.0009; CD8: p=0.0246). **B:** CD7 expression on physiologic T cells and CD4 and CD8 T cells illustrates close to 100% surface expression of CD7 on T cells. Four individual donors (A&B). Source data are provided as a Source Data file.

Supplementary Figure 14

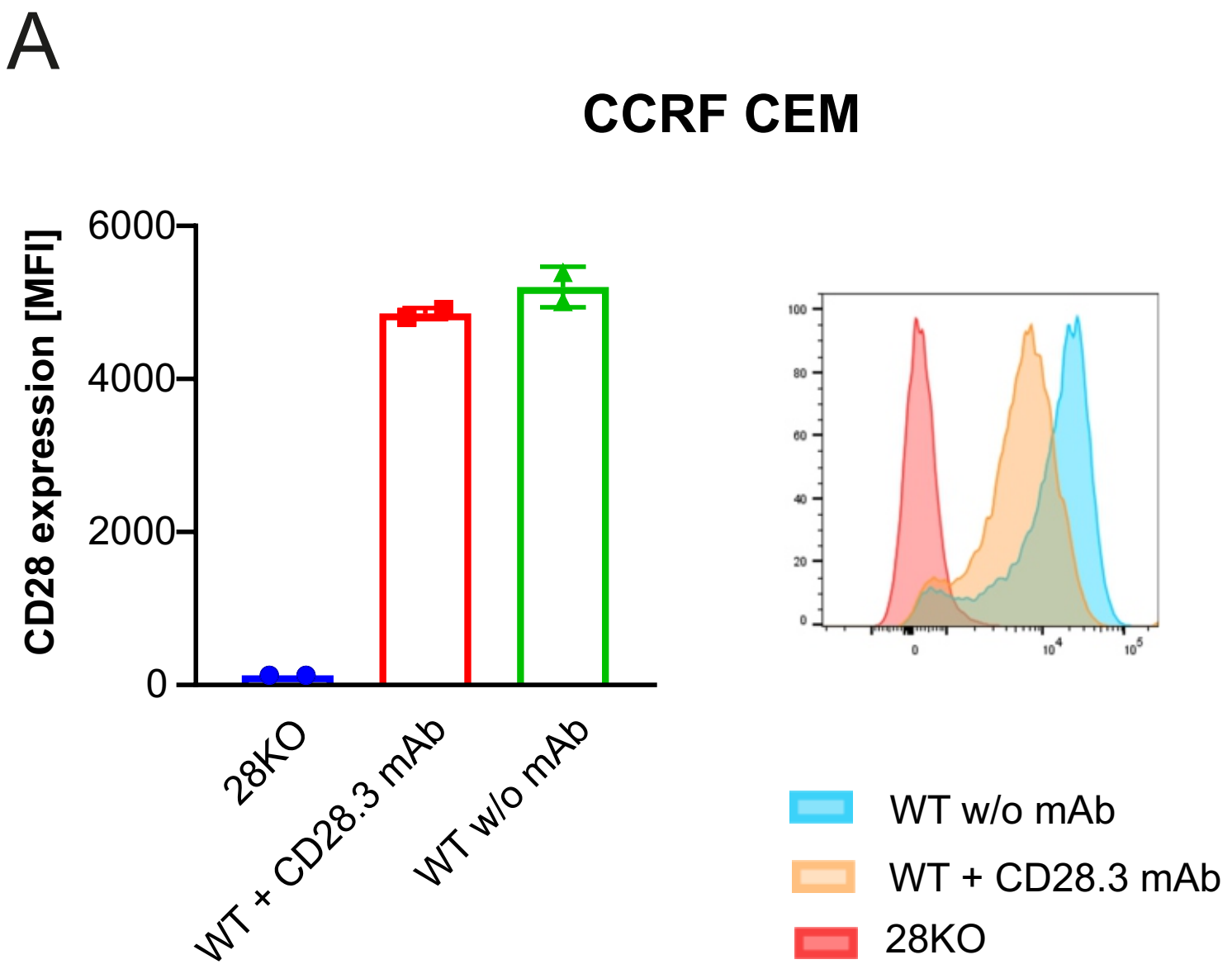

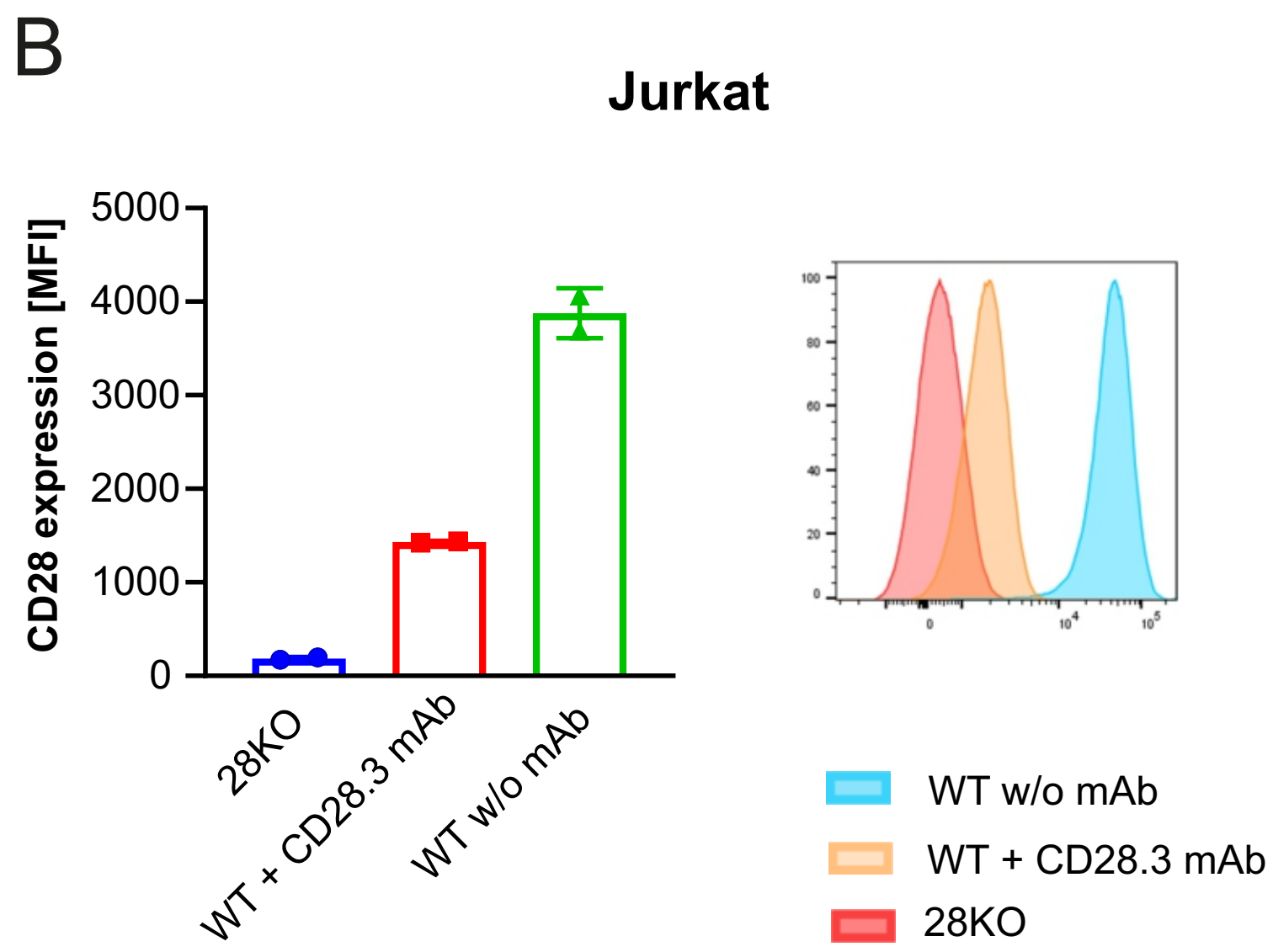

Supplementary Figure 14, A: 48 hours of incubation with CD28.3 soluble antibody led to a slight reduction of CD28 expression on CCRF-CEM. **B:** By contrast, incubation of Jurkat cells for 48 h with the same antibody reduced CD28 surface expression. Experiments were performed in technical duplicates, therefore no statistical testing was performed. Source data are provided as a Source Data file.

Supplementary Figure 15

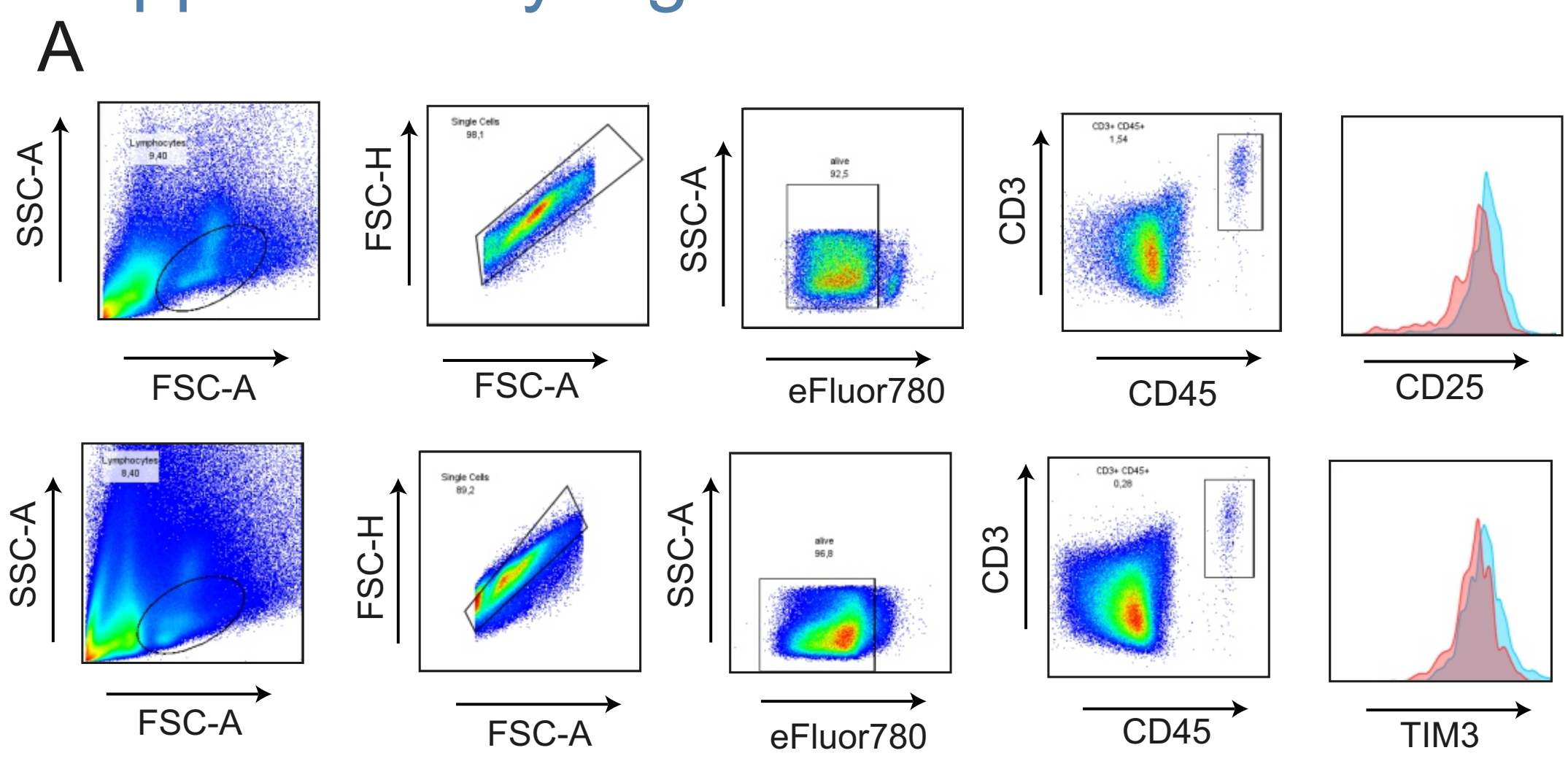

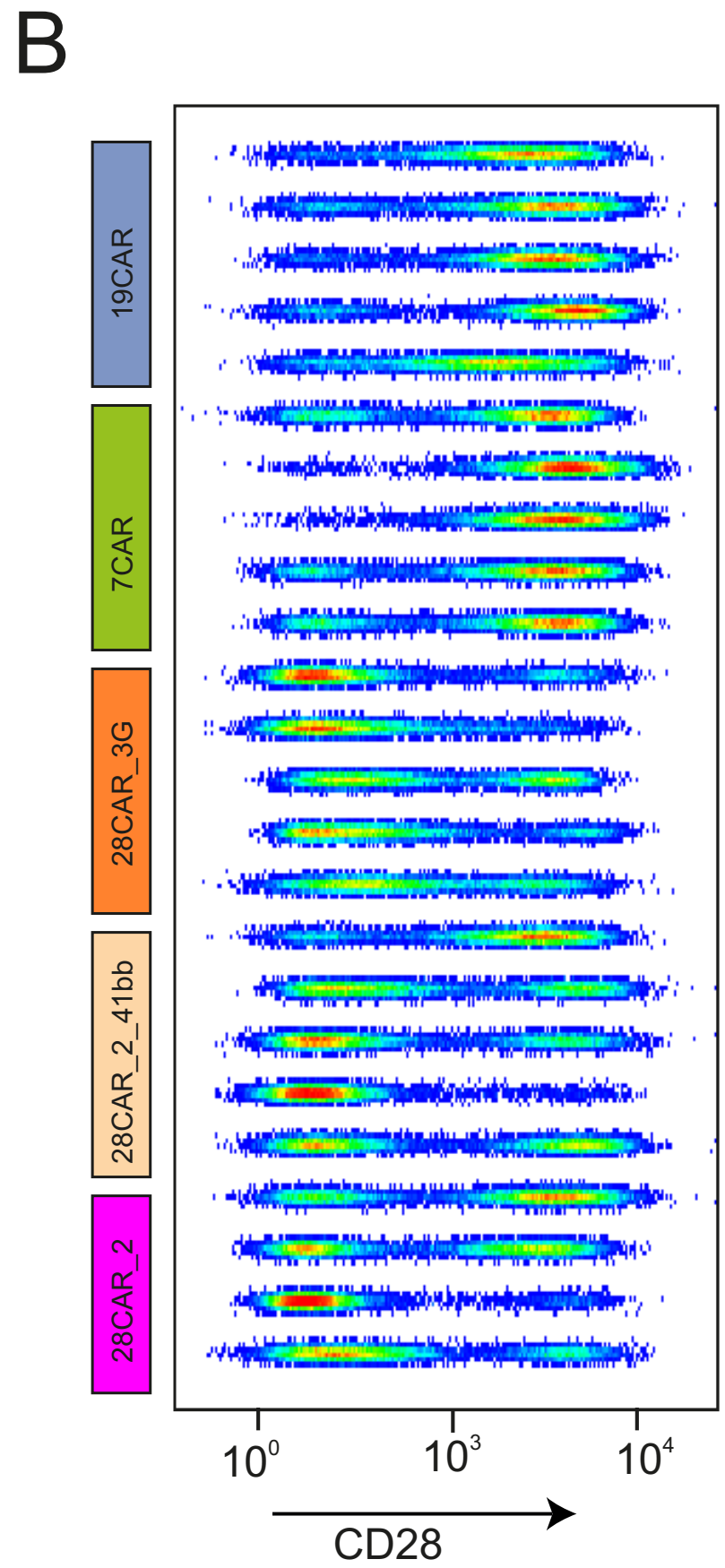

Supplementary Figure 15, A: Gating strategy for persisting CAR T cells in mouse spleen at sacrifice for TIL phenotyping (top: CD28 CAR treated mouse, lower: CD19 CAR treated mouse, bearing CCRF-CEM T-ALL). **B:** Illustration of CD28 expression by MFI in recurrent CCRF-CEM T-ALL cells from bone marrow of NXG mice injected with CCRF-CEM and subsequently treated with the indicated CAR T cell. These samples are depicted as summary data in Figure 4I. Five mice per group, four mice for 28CAR_2 treated mice.

Supplementary Figure 16

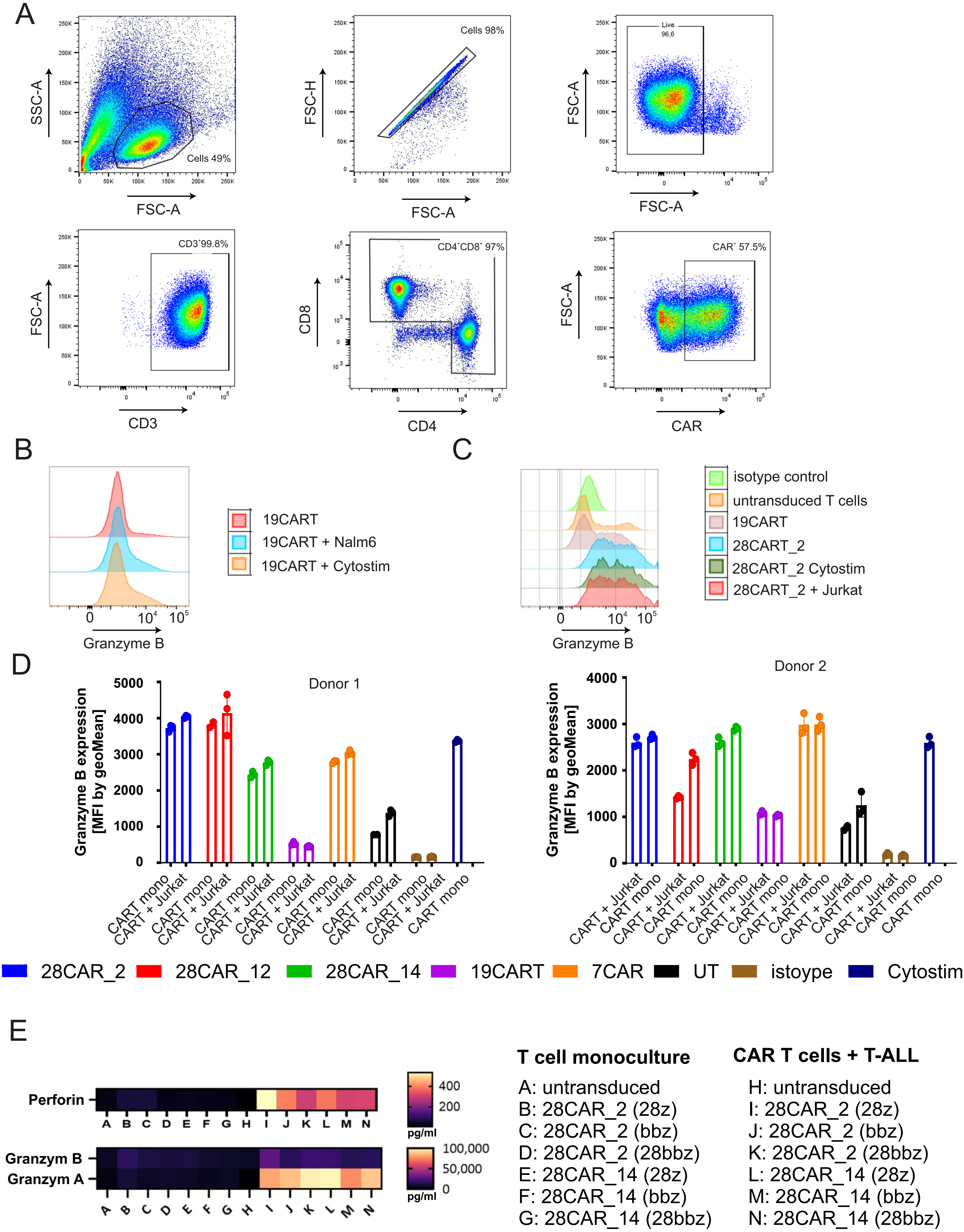

Supplementary Figure 16: Co-cultures of primary human CAR T cells and GFP-stained Jurkat cells were performed and expression of granzyme B was measured after 24 h of co-culture by intracellular cytokine staining with 4h of incubation with Brefeldin A. **A:** Gating strategy for identification of CD4 and CD8 single positive CAR$^+$ T cells by gating on lymphocytes, singlets, viable cells, CD3$^+$ cells, CD4$^+$ and CD8$^+$ single-positive cells (for exclusion of contaminating Jurkat cells) and CAR$^+$ cells (from top left to lower right). **B:** Using anti-CD19 CAR T cells (19CART), an increase of Granzyme B expressing cells was observed after co-culture with CD19$^+$ Nalm6 cells when compared to 19CAR T cells cultured alone; 19CAR T cells stimulated with Cytostim (human, Cat: 130-092-173, Miltenyi Biotec) served as positive control. **C:** Independent of co-culture with target cells, 28CAR_2 T cells show uniform, high expression of Granzyme B. Anti-CD19 CAR T cells and untransduced T cells served as controls and show significantly lower Granzyme B expression while isotype control stained 28CAR_2 T cells show no Granzyme B signal. **D:** Co-cultures of 28CAR_2, 28CAR_12, 28CAR_14, 19CAR T and 7CAR T cells were performed with Jurkat T-ALL cells, Granzyme B expression was measured as described above. Experiments were performed in technical triplicates. Granzyme B expression was high in anti-CD28 and anti-CD7 CAR T cells under both target-free and target-cell co-culture conditions. **E:** Using the LEGENDplex™ Human CD8/NK kit according to the manufacturer's instruction (Biolegend), we quantified Granzyme A and B 24 h after co-culture of anti-CD28 CAR T cells with CCRF-CEM T-ALL cell line. Co-culture with CCRF-CEM cells was associated with increased concentrations of granzyme B, granzyme A, and perforin in culture supernatants, with granzyme B reaching concentrations of up to 100 ng/ml.. Source data are provided as a Source Data file.

Supplementary Figure 17

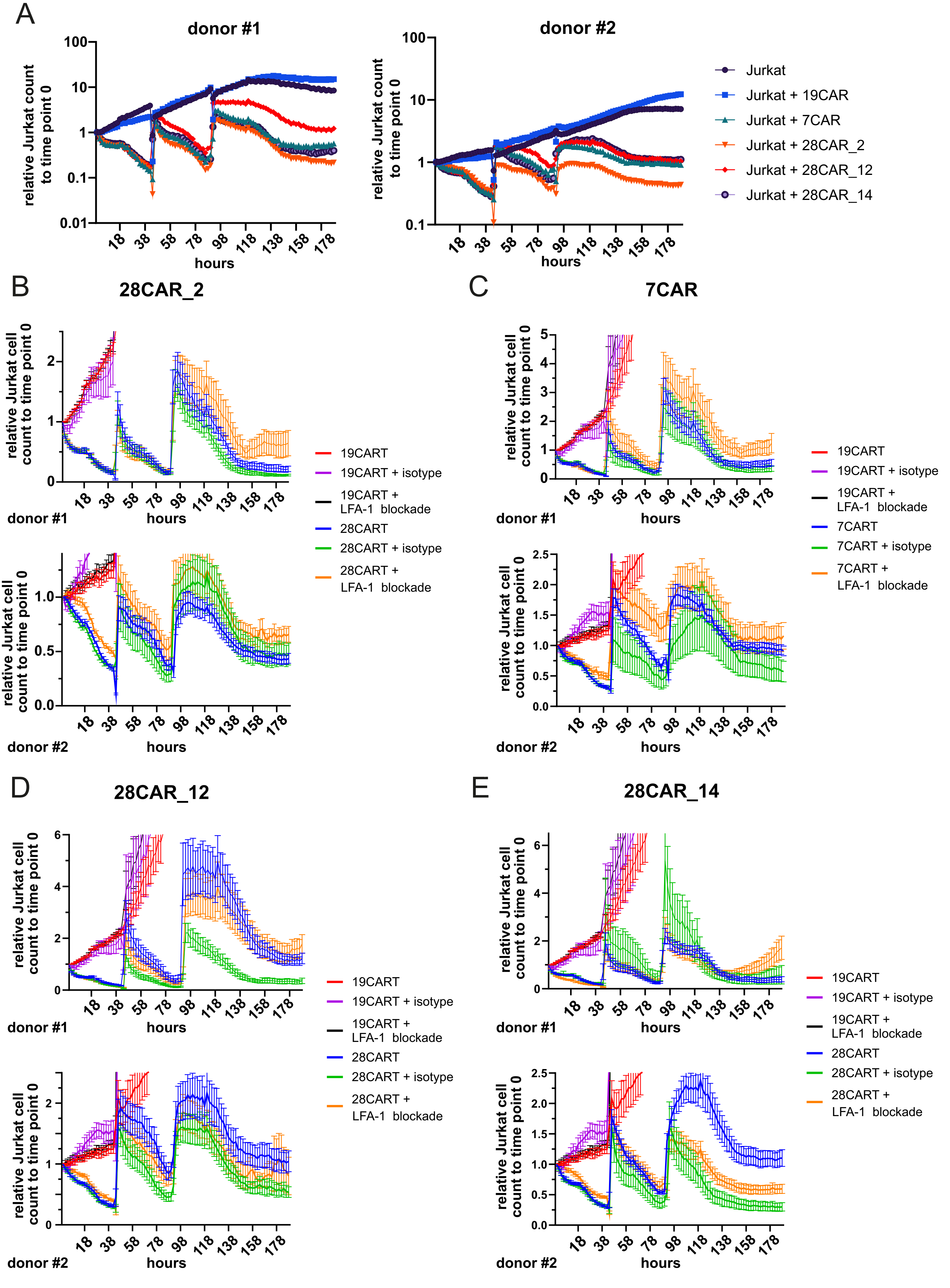

Supplementary Figure 17. Repetitive co-cultures of CAR T cells with Jurkat T-ALL in the presence or absence of LFA-1 blockade. Primary human CAR T cells (3.5×10^4 per well) from two independent donors were co-cultured with GFP-labelled Jurkat T-ALL cells (3.5×10^4 per well) either without antibody, with 10 µg/ml mouse IgG1 isotype control antibody (MAB002, R&D Systems), or with 10 µg/ml anti-human CD11a (LFA-1α; BE0192, Bio X Cell). After 48 h and 96 h, 3.5×10^4 GFP-labelled Jurkat cells were added and corresponding antibodies were replenished. **A:** Jurkat abundance in cultures without CAR T cells and in co-cultures with 19CAR, 28CAR_2, 28CAR_12, 28CAR_14 or 7CAR T cells. Two independent donors. **B:** Relative Jurkat cell count in co-cultures with 28CAR_2 T cells from two donors with either no antibody, IgG1 isotype control or anti-LFA-1. At 190 h in donor 1, mean fractions of Jurkat cells are 0.21 (CAR T cells only), 0.12 (isotype control), and 0.65 (anti-LFA-1). In donor 2, before the second stimulation, mean fractions are 0.34 (CAR T cells only), 0.34 (isotype control), and 0.48 (anti-LFA-1). **C:** Relative Jurkat abundance after co-culture with 7CAR T cells. In donor 1, at 190 h, mean fractions are 0.57, 0.35, and 1.25 for CAR T only, isotype, and anti-LFA-1, respectively. In donor 2, before second Jurkat challenge, mean fractions are 0.25, 0.27, and 0.39 for CAR T only, isotype, and anti-LFA-1, respectively. **D:** No detectable differences between antibody conditions are observed in Jurkat abundance after co-culture with 28CAR_12 T cells. **E:** Relative Jurkat cell count after co-culture with 28CAR_14 T cells. For donor 1, values after third stimulation with Jurkat T-ALL are 0.39, 0.59, and 1.70 for CAR T only, isotype, and anti-LFA-1, respectively. For donor 2, values after the first stimulation are 0.29, 0.31, and 0.46 for CAR T only, isotype, and anti-LFA-1, respectively. All time-course curves represent mean ± SEM of technical triplicates. Source data are provided as a Source Data file.

Supplementary References

1. Wang SR, Zhong N, Zhang XM, et al: OMIP 071: A 31-Parameter Flow Cytometry Panel for In-Depth Immunophenotyping of Human T-Cell Subsets Using Surface Markers. Cytometry A 99:273-277, 2021
2. Gislason MH, Demircan GS, Prachar M, et al: BloodSpot 3.0: a database of gene and protein expression data in normal and malignant haematopoiesis. Nucleic Acids Res 52:D1138-D1142, 2024
3. Suo CQ, Dann E, Goh I, et al: Mapping the developing human immune system across organs. Science 376:1069-+, 2022
