# Supplementary material for: Targeting CD28 on T lineage malignancies with chimeric antigen receptor T cells
Source: Nat Commun. 2026 Oct 8;17:10608. doi: 10.1038/s41467-026-78312-3 (PMC13646262; doi:10.1038/s41467-026-78312-3)
Supplement: Supplementary file 2 [file 41467_2026_78312_MOESM2_ESM.pdf]

nature portfolio

Corresponding author(s): Semjon Willier

Last updated by author(s): 18.08.2026

# Reporting Summary

Nature Portfolio wishes to improve the reproducibility of the work that we publish. This form provides structure for consistency and transparency in reporting. For further information on Nature Portfolio policies, see our Editorial Policies and the Editorial Policy Checklist.

## Statistics

For all statistical analyses, confirm that the following items are present in the figure legend, table legend, main text, or Methods section.

| n/a | Confirmed | |
|---|---|---|
| ☐ | ☒ | The exact sample size ($n$) for each experimental group/condition, given as a discrete number and unit of measurement |
| ☐ | ☒ | A statement on whether measurements were taken from distinct samples or whether the same sample was measured repeatedly |
| ☐ | ☒ | The statistical test(s) used AND whether they are one- or two-sided<br>*Only common tests should be described solely by name; describe more complex techniques in the Methods section.* |
| ☐ | ☒ | A description of all covariates tested |
| ☐ | ☒ | A description of any assumptions or corrections, such as tests of normality and adjustment for multiple comparisons |
| ☐ | ☒ | A full description of the statistical parameters including central tendency (e.g. means) or other basic estimates (e.g. regression coefficient) AND variation (e.g. standard deviation) or associated estimates of uncertainty (e.g. confidence intervals) |
| ☐ | ☒ | For null hypothesis testing, the test statistic (e.g. $F$, $t$, $r$) with confidence intervals, effect sizes, degrees of freedom and $P$ value noted<br>*Give P values as exact values whenever suitable.* |
| ☒ | ☐ | For Bayesian analysis, information on the choice of priors and Markov chain Monte Carlo settings |
| ☒ | ☐ | For hierarchical and complex designs, identification of the appropriate level for tests and full reporting of outcomes |
| ☐ | ☒ | Estimates of effect sizes (e.g. Cohen's $d$, Pearson's $r$), indicating how they were calculated |

*Our web collection on statistics for biologists contains articles on many of the points above.*

## Software and code

Policy information about availability of computer code

| | |
|---|---|
| Data collection | Flow cytometry data were acquired using BD FACSDiva software (BD LSRFortessa; v9.0.1), MACSQuantify software (MACSQuant Analyzer 10, Miltenyi Biotec; v2.13), and ID7000 software (Sony Biotechnology; v1.4). Luminescence data were acquired using Omega Control software (BMG LABTECH; v5.50). In vivo bioluminescence imaging data were acquired using Living Image software (Revvity; v4.7.4). Live-cell imaging data were acquired using Incucyte® software (Sartorius; versions 2023A–2025B). dSTORM images were acquired using NimOS software (ONI; v1.19). |
| Data analysis | Flow cytometry data were analyzed using FlowJo (BD Life Sciences; v10.9.0) and ID7000 software (Sony Biotechnology; v1.4). Luminescence data were quantified using MARS Data Analysis software (BMG LABTECH; v3.32), in vivo bioluminescence imaging data using Living Image software (Revvity; v4.7.4), and live-cell imaging data using Incucyte® software (Sartorius; versions 2023A–2025B). dSTORM image reconstruction and drift correction were performed using rapidSTORM (v3.3); regions of interest were selected with napari (v0.4.19), and localization and DBSCAN-based cluster analyses were performed using LOCAN (v0.16.0). Statistical analyses were performed using GraphPad Prism (v10.6.1). No custom code or custom algorithms were used. |

For manuscripts utilizing custom algorithms or software that are central to the research but not yet described in published literature, software must be made available to editors and reviewers. We strongly encourage code deposition in a community repository (e.g. GitHub). See the Nature Portfolio guidelines for submitting code & software for further information.

## Data

Policy information about availability of data

All manuscripts must include a data availability statement. This statement should provide the following information, where applicable:

- Accession codes, unique identifiers, or web links for publicly available datasets
- A description of any restrictions on data availability
- For clinical datasets or third party data, please ensure that the statement adheres to our policy

Source data are provided with this paper. Raw flow cytometry data (FCS files) generated from human donor and patient samples are not publicly available because the consent forms and biobank governance frameworks restrict unrestricted sharing of potentially re-identifiable data from small pediatric cohorts. De-identified aggregated data underlying the figures are provided in the main text, Supplementary Information and Source Data files. De-identified raw FCS files may be made available to qualified researchers for non-commercial research upon reasonable request to the corresponding author, subject to review by the responsible institutional biobank and execution of an appropriate data use agreement. Requests will be considered within  4 weeks] of receipt. The microarray dataset GSE13159 from the MILE study is publicly available through the Gene Expression Omnibus under accession code GSE13159 (https://www.ncbi.nlm.nih.gov/geo/query/acc.cgi?acc=GSE13159; https://www.fobinf.com/;).

## Research involving human participants, their data, or biological material

Policy information about studies with human participants or human data. See also policy information about sex, gender (identity/presentation), and sexual orientation and race, ethnicity and racism.

| | |
|---|---|
| Reporting on sex and gender | For animal experiments, both male and female NXG mice were used; sex was considered in the choice of model but not as an analytic variable. |
| Reporting on race, ethnicity, or other socially relevant groupings | Race, ethnicity and other socially constructed or socially relevant groupings (for example, migration background or socioeconomic status) were not systematically collected and were not used for stratification or adjustment in this study. Analyses focused on clinical and biological disease characteristics and treatment conditions. |
| Population characteristics | Population characteristics of paediatric T-ALL patients and healthy bone marrow donors are summarized in Table 1, including age, sex and disease status. Participants were children treated at LMU Munich hospital with newly diagnosed or relapsed T-ALL and age-matched paediatric bone marrow donors without evidence of haematological disease.<br>Patient samples were obtained from the Hauner Hematology Biobank and COALL; healthy bone-marrow samples were obtained from age-matched paediatric donors without haematological disease. Samples were included based on availability and sufficient post-thaw viability for the planned analyses. No prospective recruitment or participant self-selection occurred. Use of banked samples and variability in post-thaw viability may introduce selection bias and limit generalizability of CD28-expression frequencies to the broader paediatric T-ALL population. |
| Recruitment | Paediatric T-ALL patients and healthy bone marrow donors were recruited at LMU Munich through clinical care pathways, and samples were provided by the Hauner Hematology Biobank and the COALL study group. Consecutive eligible patients and age-matched donors with available surplus diagnostic bone marrow were included over the period 2000 - 2017. Recruitment via tertiary care centres and study biobanks may favour more complex cases, which could limit the generalizability to all children with T-ALL. |
| Ethics oversight | The study involving human participants and human biological material was approved by the Ethikkommission bei der LMU München (reference 17-163) and the ETHIKKOMMISSION of the Albert-Ludwigs-Universität Freiburg (reference 24-1047_3S1). All procedures were conducted in accordance with the Declaration of Helsinki, and written informed consent was obtained from participants or their legal guardians, or analyses were performed on anonymized surplus diagnostic samples as approved by the ethics committees. |

Note that full information on the approval of the study protocol must also be provided in the manuscript.

# Field-specific reporting

Please select the one below that is the best fit for your research. If you are not sure, read the appropriate sections before making your selection.

[x] Life sciences [ ] Behavioural & social sciences [ ] Ecological, evolutionary & environmental sciences

For a reference copy of the document with all sections, see nature.com/documents/nr-reporting-summary-flat.pdf

# Life sciences study design

All studies must disclose on these points even when the disclosure is negative.

| | |
|---|---|
| Sample size | No formal statistical sample-size calculation was performed. Sample sizes for patient and healthy donor cohorts, in vitro experiments and in vivo mouse studies were based on prior experience with similar assays and on the availability of clinical material, and are consistent with previous studies in the field. The exact sample size (n) for each experimental group and condition, and the unit of analysis (patients, independent samples, or animals), is reported in the figure legends and Methods. |
| Data exclusions | No data or samples were excluded from the analyses. |

| | |
|---|---|
| Replication | Key experiments were repeated as follows, with consistent results across replicates. In vitro CAR functional assays (cytotoxicity, expansion, transduction, cytokine release, phenotyping) were performed using 3–4 independent healthy T-cell donors per experiment (Fig. 2B–H, J–M: 3 donors; Fig. 3C–E, I: 4 donors; Fig. 3F, J–L: 3 donors; Fig. 5A–H: 3–4 donors). Cellular avidity measurements (z-Movi, Fig. 3H) were performed independently in four paired replicates on the same chip. Patient and healthy-donor cohort comparisons were based on independent biological samples rather than technical repeats (Fig. 1D: n=52 T-ALL vs. n=14 healthy; Fig. 1E–F, Fig 5I: n=17–50 depending on data availability). In vivo experiments were performed with 5 mice per group (Fig. 4C, F–H, I–J) or 15 mice per CAR construct pooled across costimulatory domains (Fig. 4E), and key findings (superior survival of anti-CD28 and anti-CD7 CAR T cells over anti-CD19 controls) were independently reproduced across two institutions (LMU Munich and University Medical Center Freiburg) and across three tumor models (CCRF-CEM, Jurkat, ALL-230 PDX; Fig. 4K). The exact number of biological replicates for each experiment is reported in the corresponding figure legend. Results were consistent across the independent biological replicates reported. |
| Randomization | Human patient and healthy donor samples were not allocated into experimental groups; all available eligible samples were analyzed as received, since group membership (T-ALL vs. healthy) reflects a fixed biological characteristic rather than an assignable variable. For in vitro experiments (co-culture assays, flow cytometry, cytokine secretion, CRISPR/Cas9 knockout, CAR transduction comparisons, cellular avidity and dSTORM imaging), cells were derived from a single donor or cell-line preparation and split evenly across experimental conditions (e.g., gRNA vs. non-binding control, or CAR construct A vs. B), representing a matched/paired design rather than independent randomized allocation; this approach controls for donor-to-donor and batch variability more effectively than randomization across separate donors would. For in vivo experiments, mice were allocated to treatment groups before CAR T-cell injection; formal randomization was not performed. Mice were age-matched, obtained from a single supplier, and both sexes were included. No additional covariate stratification was applied, as mice were of matched age and sex distribution across cohorts and obtained from a single commercial supplier (Janvier Labs) |
| Blinding | No blinding was performed. Investigators were aware of group allocation during data collection and analysis, and blinding was not implemented for these mechanistic in vitro assays and in vivo T-ALL experiments. |

# Reporting for specific materials, systems and methods

We require information from authors about some types of materials, experimental systems and methods used in many studies. Here, indicate whether each material, system or method listed is relevant to your study. If you are not sure if a list item applies to your research, read the appropriate section before selecting a response.

## Materials & experimental systems

| n/a | Involved in the study | |
|---|---|---|
| ☐ | ☒ | Antibodies |
| ☐ | ☒ | Eukaryotic cell lines |
| ☒ | ☐ | Palaeontology and archaeology |
| ☐ | ☒ | Animals and other organisms |
| ☐ | ☒ | Clinical data |
| ☒ | ☐ | Dual use research of concern |
| ☒ | ☐ | Plants |

## Methods

| n/a | Involved in the study | |
|---|---|---|
| ☒ | ☐ | ChIP-seq |
| ☐ | ☒ | Flow cytometry |
| ☒ | ☐ | MRI-based neuroimaging |

## Antibodies

| | |
|---|---|
| Antibodies used | All primary and secondary antibodies and viability dyes used are listed in Supplementary Table 1, including target, clone, fluorochrome or conjugate, supplier, catalogue number, and final dilution or concentration. Lot numbers were not systematically recorded and are therefore not available. Commercial antibodies were selected based on manufacturer validation for the stated application where available. All antibodies used for flow cytometry were titrated to determine the optimal working concentration before use. Fluorescence-minus-one controls were routinely used during multicolour panel establishment and for critical gating decisions to define positivity thresholds. Antibody performance was additionally assessed using biologically appropriate antigen-positive and antigen-negative populations; CRISPR/Cas9 CD7- or CD28-knockout cells were used where applicable. Validation details are provided in Supplementary Table 1 and the Methods. |
| Validation | All antibodies used in this study were commercially available and were selected based on manufacturer validation for the respective application and human samples, where applicable. Detailed validation information, including supplier, catalogue number, clone, application, and final working dilution or concentration, is provided in Supplementary Table 1. All flow-cytometry antibodies were titrated before use to determine the optimal working concentration. Fluorescence-minus-one controls were routinely used during multicolour panel establishment and for critical gating decisions. For CD7 and CD28 flow-cytometry staining, specificity was additionally supported by CRISPR/Cas9-mediated knockout of the respective target, together with antigen-positive and antigen-negative cell populations. No newly generated antibodies were used in this study. |

## Eukaryotic cell lines

Policy information about cell lines and Sex and Gender in Research

| | |
|---|---|
| Cell line source(s) | CCRF-CEM (DSMZ ACC 240; female donor), Jurkat (DSMZ ACC 282; male donor), RPMI-8226 (DSMZ ACC 402; male donor) and MM.1S (ATCC CRL-2974; female donor) were used. The retroviral packaging cell lines 293Vec-Galv and 293Vec-RD114 were provided by BioVec Pharma Inc. (Canada). The previously established patient-derived T-ALL xenograft model ALL-230 was provided by Irmela Jeremias; sex of the original donor was not available to the authors. No cell lines were newly established from primary human or vertebrate cells in this study. |

| | |
|---|---|
| Authentication | All human cell lines were routinely authenticated by short tandem repeat (STR) profiling at DSMZ. |
| Mycoplasma contamination | Cell lines were regularly tested mycoplasma negative using the Venor GEM Mycoplasma Detection PCR kit. |
| Commonly misidentified lines (See ICLAC register) | No commonly misidentified cell lines, as defined by the ICLAC register, were used in this study. |

## Animals and other research organisms

Policy information about studies involving animals; ARRIVE guidelines recommended for reporting animal research, and Sex and Gender in Research

| | |
|---|---|
| Laboratory animals | Female and male NXG mice (Mus musculus; NOD- Prkdcscid-IL2rgTm1/Rj; NOD genetic background; Janvier Labs) were used in all in vivo experiments. Mice were 6–12 weeks old at study initiation. Mice were maintained under SPF conditions with free access to food and water, a 12-h light/12-h dark cycle (dark phase 18:00–06:00), ambient temperature of 20–24 °C, and relative humidity of 45–65%. |
| Wild animals | No wild animals were used in this study. |
| Reporting on sex | In the T-ALL mouse experiments, bot male/female NXG mice were used; sex was not included as an analytical variable. |
| Field-collected samples | This study did not involve samples collected from the field. |
| Ethics oversight | All animal experiments were approved by the Regierung von Oberbayern (ROB-55.2-2532.Vet_02_21-58) and the Regierungspräsidium Freiburg (G25-001) and carried out in accordance with institutional and national guidelines for animal care and use. |

Note that full information on the approval of the study protocol must also be provided in the manuscript.

## Clinical data

Policy information about clinical studies

All manuscripts should comply with the ICMJE guidelines for publication of clinical research and a completed CONSORT checklist must be included with all submissions.

| | |
|---|---|
| Clinical trial registration | Clinical trial registration is not applicable, as this study is not an interventional clinical trial but uses patient samples and retrospective clinical data. |
| Study protocol | A detailed study protocol was not separately preregistered; all experimental procedures and analysis plans are described in the Methods section of the manuscript and were approved by the relevant ethics committees. |
| Data collection | Patient samples were obtained from the Hauner Hematology Biobank and COALL; healthy bone-marrow samples were obtained from age-matched paediatric donors without haematological disease. Samples were included based on availability and sufficient post-thaw viability for the planned analyses. No prospective recruitment or participant self-selection occurred. Use of banked samples and variability in post-thaw viability may introduce selection bias and limit generalizability of CD28-expression frequencies to the broader paediatric T-ALL population.” |
| Outcomes | N/A |

## Plants

| | |
|---|---|
| Seed stocks | N/A |
| Novel plant genotypes | N/A |
| Authentication | N/A |

## Flow Cytometry

### Plots

Confirm that:

- [x] The axis labels state the marker and fluorochrome used (e.g. CD4-FITC).
- [x] The axis scales are clearly visible. Include numbers along axes only for bottom left plot of group (a 'group' is an analysis of identical markers).
- [x] All plots are contour plots with outliers or pseudocolor plots.
- [x] A numerical value for number of cells or percentage (with statistics) is provided.

### Methodology

| | |
|---|---|
| Sample preparation | Mononuclear cells from patient and healthy donor bone marrow were isolated by density gradient centrifugation, cryopreserved and stored in liquid nitrogen; before staining, cells were thawed, washed in PBS and stained with the antibody panels listed in Supplementary Tables and Methods. For CAR T cell generation, freshly isolated PBMCs were used. For co-culture assays, target cells and CAR T cells or untransduced T cells were harvested from culture, washed and stained after the specified incubation period; for PBMC co-cultures, CTV-labelled CAR T cells were co-cultured with autologous PBMCs and then stained with a 25-colour spectral panel prior to acquisition on the ID7000. |
| Instrument | Flow cytometry data were acquired on a BD LSR Fortessa (BD Biosciences) and a MACSQuant Analyzer 10 (Miltenyi Biotec), and spectral flow cytometry data were acquired on an ID7000 Spectral Cell Analyzer (Sony Biotechnology). |
| Software | Flow cytometry data were acquired using the respective instrument software and analysed using FlowJo X (FlowJo LLC) and the ID7000 analysis software (Sony Biotechnology); statistical analyses and graphing of flow-cytometry-derived data were performed with GraphPad Prism 10. |
| Cell population abundance | For flow cytometry experiments, ≥10,000 events per sample (and up to 10^7 PBMCs for spectral panels) were acquired and analysed for each condition; the abundances (counts or percentages) of the relevant cell populations are quantified in the main and Supplementary Figures as indicated in the figure legends. |
| Gating strategy | Gating strategies (including exclusion of debris, doublets and dead cells, and sequential identification of lymphocyte and CAR T-cell subsets) are shown in Supplementary Fig. 15, and the such gates were applied across all comparable samples and conditions |

- [x] Tick this box to confirm that a figure exemplifying the gating strategy is provided in the Supplementary Information.
