# Supplementary material for: Targeting CD28 on T lineage malignancies with chimeric antigen receptor T cells
Source: Nat Commun. 2026 Oct 8;17:10608. doi: 10.1038/s41467-026-78312-3 (PMC13646262; doi:10.1038/s41467-026-78312-3)
Supplement: Supplementary file 3 [file 41467_2026_78312_MOESM3_ESM.pdf]

nature portfolio

# Peer Review File

# Targeting CD28 on T lineage malignancies with chimeric antigen receptor T cells

Corresponding Author: Dr Semjon Willier

**This file contains all reviewer reports in order by version, followed by all author rebuttals in order by version.**

Version 0:

Reviewer comments:

Reviewer #1

(Remarks to the Author)
This paper presents a case for CD28 as a novel target for CAR T-cell therapy in T-lineage malignancies, supported by extensive preclinical data and innovative approaches to overcome common challenges in CAR T-cell therapies. The paper successfully identifies CD28 as a novel and promising target for CAR T-cell therapy in treating T-cell malignancies such as T-cell acute lymphoblastic leukemia (T-ALL) and T-cell lymphomas. This is a significant advancement, as there are limited therapeutic options available for these cancers. For the most part, the data is presented clearly and systematically, with extensive use of flow cytometry, immunohistochemistry, and statistical analysis to substantiate the findings. The inclusion of supplementary data further supports the robustness of the experimental results. The study does not sufficiently address these long-term challenges, which is a critical oversight given the high relapse rates associated with existing CAR T-cell therapies. Several significant issues still need to be addressed.

Specific points

1) Figure 1: Panel B shows expression of CD28 at what looks like moderate levels; however the authors need to include a numbered axis to allow one to know. Further, there appears a CD28 negative subset with the T-ALL population suggesting that cells might simply escape the elimination of CD28-CARs? Is this not possible? Also, the levels of CD28 expression on healthy donors seems very low? It would be help to see both the MFI values and the % expression values outlined more clearly in different T-ALL samples taken from different patients, perhaps a Table. Also Fig 1D needs to show IFNgamma, TNFalpha, granzyme B and perforin expression levels. The study effectively compares anti-CD28 CAR T cells with anti-CD7 CAR T cells, which are currently used in the treatment of T-ALL. This comparison provides a clear context for evaluating the relative efficacy and safety of the new therapy.
3) Figure 2: panel J- interesting that there is such variation in IFNgamma production. Do the authors understand why? Is TNFalpha also variable as well as granzyme B and perforin expression? Panel J should also include data in endocytosis-do they vary amongst the CARs.
4) Figure 2: panel K,L the CAR 2 and 12 patterns of relative killing does not correlate with IFNgamma and therefore it is important to see whether other killing mechanisms are at work- also can one block with anti-IFNgamma?
5) Figure 3: Within the same context, this informative figure includes perforin, but to enhance the paper's credibility, it would greatly benefit from FACs analysis showing that the expression levels of perforin and granzyme B are indeed elevated in the more cytolytic CARs. Additionally, it is essential to include data on proliferation, which can be effectively showcased through the expression profiles of Ki67.
6) Figure 4: looks good but would benefit from a characterisation of the phenotype of the TILs associated with the CARs.
7) The paper lacks detailed comparative data on the toxicity and cytokine release syndrome (CRS) profiles of anti-CD28 CAR T cells relative to other CAR T-cell therapies.
8) Utilizing un-transduced T cells or T cells transduced with a mock CAR (lacking a specific targeting domain) would establish a baseline for understanding the effects of CAR transduction and expansion without the confounding influence of specific antigen targeting.
9) Although the study mentions the potential for lower on-target off-tumor toxicity compared to CD7 targeting, more discussion related to this issue is needed to fully understand and mitigate these risks in clinical settings, particularly concerning the preservation of immune system integrity.

Reviewer #2

(Remarks to the Author)
Dr. Willier and co-authors at LMU identified CD28 as a therapeutically actionable target in T-ALL. They showed CAR T cells targeting CD28 can expand following CD28 editing and produce cytotoxicity against T-ALL cell lines in vitro. In a CCRF-CEM xenograft model, intravenously administered CD28 CART prolong animal survival and delay systemic leukaemia, acting on par with a control CD7 CAR. Most clinically successful CAR-T therapies in T-ALL target CD7 and CD5; CD28 is a new target in this space, which aids in novelty.
While this preclinical report is mostly straightforward, several unaddressed issues limit our understanding of the translational relevance of this work.

1. As the authors mention repeatedly throughout the paper, most normal T cells express CD28. In lines 244-247, the authors state "CD28 as opposed to CD7 is not
expressed on all physiologic T cells, which is why T-cell depletion and thereby T-cell
immunodeficiency would supposedly be less pronounced after anti-CD28 CAR T-cell
therapy". First, this statement must be corrected as CD7 is also not expressed in all T cells, and multiple clinical studies with CD7 CART show systemic enrichment for CD7-negative T cells after infusion. Second, while I truly hope that is true, no data is provided to back the authors' claim about CD28.
(a) Comparing CD28 expression level, both per cent and MFI, in T-ALL and in normal CD45+ mature T cells in peripheral blood should be included to understand the degree of CD28 overexpression in T-ALL blasts. Supplementary Fig 1D/E shows relative frequency in bone marrow but it is difficult to understand whether this reflects global picture and whether the expression is brighter on the malignancy.
(b) I am confused why the authors compare CD28 levels in T-ALL vs normal CD7+ CD45dim cells in bone marrow? Committed T-cell progenitors reside in the thymus, not in the marrow. The CD7+ BM population could be comprised of mixed lineage (including NK and myeloid) cells and progenitors, only some of which could be heading down the T-cell lineage.
(c) Coculture assays of CD28 CART with freshly isolated blood T cells should be performed. The surviving CD28-negative T cell population (if exists) should be characterized for subset composition to estimate the extent of T-cell aplasia in patients and the quality/functional status of therapy-resistant lymphocytes.
(d) Potential additional ablation of plasma B cells is a concern. Do the authors also expect total reset of that population - or this therapy is designed mainly as a bridge to alloHSCT? Discussing these aspects would help the reader understand whether CD28 CART will be safer than CD7 targeting in T-ALL.

2. CD28 modulation on recurrent CCRF in vivo is interesting. Adding a reference control would help understand if the downregulation is partial or complete; flow plots would be also helpful. Does this reflect a selection of a rare CD28dim/negative CCRF clone, or a genuine CD28 downregulation on leukaemia? Testing a couple of additional T-ALL xenografts, ideally patient-derived, can help understand how common this mechanism is, and whether it constitutes a general hurdle in therapeutic CD28 targeting.
3. The Results section pertaining to Figure 2 is all over the place. It starts with 2B, then 2G-I, then 2A and so on. I could not find a description of the experiment in Figure 2F. Figure 3A is also not referenced. 4D comes before 4C. Can the authors describe the figure in the order the panels are shown?
4. The authors' results suggest CD28 CARs mask their own CD28 which could aid in fratricide evasion. Assuming the CD28 KO efficiency in cGMP is not 100%, this leaves the possibility of "contaminating" CD28wt CD28 CART cells in the final product. Would these unedited CD28 CART be constantly revved up upon continuous cis-engagement of the CD28 superagonist binder and possibly cause a cytokine storm? If so, discussing potential mitigation strategies will help the reader.
5. The pattern of CD28 vs 2B4 expression is intriguing but I would not call it a meaningful correlation as only 1/3 (35%) of all data are explained by this regression.
6. While the brief in vitro data on CD28 targeting in MM cell lines is of interest, stating in the abstract that CD28 is a novel immunotherapy target in MM warrants a deeper investigation.

Minor comment
1. To my knowledge, the 3A1f clone of CD7 mAb used for a control CD7 CAR in this study has not been validated clinically due to better-performing binders. I could be wrong.

Reviewer #3

(Remarks to the Author)
The authors detail the expression of CD28 on T-ALL in pediatrics and evaluate it as a novel target of CAR T cells. They do a thorough evaluation of CD28-CAR T cell utility with benchmark comparison to CD7. Novel aspects of this study include the generation of CD28ko cells which appear to retain functionality, although do have a loss of cytotoxicity in vitro and development and evaluation of CD28 specific CAR T cells which could potentially be taken to clinical trials.

Intro – there isn't any mention of previous work looking at CD28 expression in pre-T malignancies. This should be mentions. Consider PMCID 8748252

Line 94- Is there something missing after CD7? "than both CD7"

For the experiment of CD28 ligation by CD86 – is it possible to culture with monocytes that doesn't have CD86 co-expression to compare? As the CD28 ko cell did not observe any proliferation change, was this done as well with the monocytes expressing CD86? My read is that the beginning is looking to see that ligation of CD28 and CD86 confers a proliferation signal, and this seems to be accounted for, at least in part, though IL-7. Did you look in the supernatant of the DC+CCRF-CEM to determine IL-7 levels, and in comparison to the CCRF-CEM culture and DC culture alone? Meaning can you confirm higher levels of IL-7 and also that it is a direct result of engagement of the DC+CCRF-CEM. I don't believe that this evaluation is of utmost importance for the value of this paper, but I do think the statement of IL-7 accounting for the effect needs to be shown more thoroughly if it is going to be stated.

Figure 1B – from the gating – it is showing the expression of CD28 on the CD7+ gate or of the sCD3 gate - It seems like the later but the way it is presented is confusing and seems like it is only showing CD28 expression on the CD7 gate. If it is of just the CD7 gate, it would seem helpful to also show the CD28 expression on all T cells.

Figure 1C – what does it look like in comparison to CD7? In looking at supplemental figure, its presenting the expression on all bone marrow T cells and it looks like a substantial amount of both CD7 and CD28 is present. Why make the distinction of the expression of C28 on BMT cells v progenitor T cells?
Figure 1D – is it possible to include CD7 and CD5 in this analysis?

Is there a decrease in cytotoxicity when signaling through a CAR in T cells with CD28 k/o? This is critically important to show that the CAR T cells will have high functionality in vivo. Did you do CD28k/o in a CD7 CAR T cells and compare its functionality? I can't follow figure 3 CDEFGH to understand which cells in the fold expansion and target killing had D28 and CD7 k/o. Perhaps this is all there already and just needs to be made more explicit.

I do not understand the relevance of the TGN1412 experiment (lines 157-162). Perhaps there is a different way to stress the point made in this experiment. I presume this links to the importance of lines 184-186 but I am not following the T cell independent effect on ALL.

Discussion – if loss of CD28 leads to decreased cytotoxicity of T cells, what are the implications of CD28k/o T cells – this should at least be discussed. Potentially when discussing about CD7 CAR T cells and potential advantages of using CD28 CAR T cells.

Version 1:

Reviewer comments:

Reviewer #1

(Remarks to the Author)
That authors have addressed carefully most of my comments. It is a good paper,
1) however, it is a shame that the paper is missing clear data on mechanism. The showed the correlation between interferon gamma and killing but to their credit they found that the addition of blocking antibodies did not affect killing. Unfortunately, the paper does not appear to show a TNF-α blocking assay. Since TNF-α can directly induce apoptosis in some tumor cells--did they try this straightforward experiment?
If not, the clearly perforin-GZM is most likely--they show some perforin correlation data however this evidence in support of this pathway would be clearer with some data on granzyme A or B expression (if possible) or the blocking of cell-cell contact with anti-LFA-1 needed for perforin-GZM killing or some data showing in vitro killing of targets with the CARs...
This may not be obligatory for acceptance but it would greatly improve the paper and the importance attached to the paper--something that demonstrates how CAR CD28 is operating mechanistically--
2) the introduction on CD28 needs a major improvement with more information on CD28, how it signals via PI 3K, GRB2, Lck-PKCtheta, mRNA stability, issues related to previous clinical work with autoimmunity and its role in different contexts with the corresponding references (June, Thompson, Rudd, Altman etc.) --this is important--

Reviewer #2

(Remarks to the Author)
The authors have addressed the comments

Reviewer #3

(Remarks to the Author)
The authors have appropriately addressed my comments.

Version 2:

Reviewer comments:

Reviewer #1

(Remarks to the Author)
The revised manuscript is significantly improved, and the authors have addressed several key areas. However, the mechanistic data requires further clarification and data presentation before publication.

1) It provides good data related to the lack of involvement of the TNFalpha/interferon pathway in killing tumors, strongly suggesting the involvement of the GZMB/A-perforin pathway.

2) The assertion that anti-LFA-1 blockade has no effect on cytotoxicity is highly unexpected, given that the vast majority of cytotoxic T lymphocyte (CTL) killing relies—at least in part—on this classic adhesion pathway. While alternative mechanisms may be at play, the current manuscript offers no data to support this conclusion beyond a text statement: lines 183-186: "Interestingly, blockade of lymphocyte function-associated antigen 1 (LFA-1) by a blocking antibody did not significantly hamper anti-CD28 CAR T cell cytotoxicity against T-ALL in vitro (data not shown) indicating that this adhesion molecule is dispensable for anti-CD28 CAR T cell killing." The need to include the data, even as a supplementary figure and include proper positive controls demonstrating that the concentration of the anti-LFA-1 blocking antibody used are biologically active and sufficient to successfully inhibit LFA-1 binding/adhesion in their experimental setup. Without this control, it is not really possible to determine whether LFA-1 is truly dispensable or if the blockade was simply ineffective.

**Open Access** This Peer Review File is licensed under a Creative Commons Attribution 4.0 International License, which permits use, sharing, adaptation, distribution and reproduction in any medium or format, as long as you give appropriate credit to the original author(s) and the source, provide a link to the Creative Commons license, and indicate if changes were made.
In cases where reviewers are anonymous, credit should be given to 'Anonymous Referee' and the source.
The images or other third party material in this Peer Review File are included in the article's Creative Commons license, unless indicated otherwise in a credit line to the material. If material is not included in the article's Creative Commons license and your intended use is not permitted by statutory regulation or exceeds the permitted use, you will need to obtain permission directly from the copyright holder.
To view a copy of this license, visit https://creativecommons.org/licenses/by/4.0/

REVIEWER COMMENTS // AUTHOR RESPONSE

**Reviewer #1 (Remarks to the Author): with expertise in T-cell biology, immunotherapy**

This paper presents a case for CD28 as a novel target for CAR T-cell therapy in T-lineage malignancies, supported by extensive preclinical data and innovative approaches to overcome common challenges in CAR T-cell therapies. The paper successfully identifies CD28 as a novel and promising target for CAR T-cell therapy in treating T-cell malignancies such as T-cell acute lymphoblastic leukemia (T-ALL) and T-cell lymphomas. This is a significant advancement, as there are limited therapeutic options available for these cancers. For the most part, the data is presented clearly and systematically, with extensive use of flow cytometry, immunohistochemistry, and statistical analysis to substantiate the findings. The inclusion of supplementary data further supports the robustness of the experimental results. The study does not sufficiently address these long-term challenges, which is a critical oversight given the high relapse rates associated with existing CAR T-cell therapies. Several significant issues still need to be addressed.

Specific points

1) Figure 1: Panel B

shows expression of CD28 at what looks like moderate levels; however the authors need to include a numbered axis to allow one to know. Further, there appears a CD28 negative subset with the T-ALL population suggesting that cells might simply escape the elimination of CD28-CARs? Is this not possible? Also, the levels of CD28 expression on healthy donors seems very low? It would be help to see both the MFI values and the % expression values outlined more clearly in different T-ALL samples taken from different patients, perhaps a Table. Also Fig 1D needs to show IFNgamma, TNFalpha, granzyme B and perforin expression levels. The study effectively compares anti-CD28 CAR T cells with anti-CD7 CAR T cells, which are currently used in the treatment of T-ALL. This comparison provides a clear context for evaluating the relative efficacy and safety of the new therapy.

We thank the reviewer for the positive evaluation of our work and the recognition of CD28 as a promising therapeutic target. We appreciate the constructive feedback and have made the following changes to address the reviewer's concerns.

**Regarding the presentation of CD28 expression data.** We have revised Figure 1B (now Figure 1C) to improve clarity and transparency of our flow cytometry data. The panel now displays pseudocolor dot plots showing CD28 expression for both healthy donors (HD) and T-ALL patient samples. The original Figure 1B has been relocated to Supplementary Figure 1A. We acknowledge that some T-ALL patient samples were analyzed using antibodies conjugated with different fluorochromes (BV650 instead of FITC). To ensure accurate representation, these samples are now depicted in a separate plot (Supplementary Figure 1B) showing median fluorescence intensity (MFI) values for individual patients.

**Regarding long-term challenges and antigen escape at relapse.** We appreciate the reviewer's emphasis on addressing long-term therapeutic challenges and the risk of antigen-negative relapse, which are indeed critical considerations for CAR-T cell therapy development. While pre-existing CD28-negative subclones cannot be entirely excluded, several factors suggest this is unlikely to represent a major clinical challenge: First, T-ALL is a clonal disease arising from a single transformed progenitor cell, similar to B-ALL. Second, our gating strategy ($CD45^{dim}$, $CD7^{+}$, $sCD3^{-}$) captures both T-ALL blasts and physiological lymphoid progenitors, meaning that CD28-negative events likely represent residual normal progenitor cells rather than malignant subclones. We would like to highlight that our study includes direct experimental evidence addressing this concern. In Figure 4K, we demonstrate that CD28

expression is maintained at relapse in two independent in vivo T-ALL models. This finding suggests that, unlike other target antigens where antigen-negative escape variants frequently emerge, CD28 expression remains stable even under selective pressure from CAR-T cell therapy.

3) Figure 2: panel J

interesting that there is such variation in IFNgamma production. Do the authors understand why? Is TNFalpha also variable as well as granzyme B and perforin expression? Panel J should also include data in endocytosis-do they vary amongst the CARs.

We thank the reviewer for this important observation regarding the variability in IFNγ production among different CD28-CAR constructs.

**Regarding IFNγ production variability:** The observed variation in IFNγ levels among different CD28-CAR-T cell cells (now Figure 2H) can be attributed to several technical and functional parameters. Notably, constructs 28CAR_1 and 28CAR_11, both harboring the short hinge domain, showed no detectable surface CAR expression when assessed via the myc-tag epitope. Consequently, for these constructs, IFNγ expression was quantified on total T cells rather than specifically on CAR-T cells, as gating on $CAR^{+}$ cells was not feasible in 28CAR_1 and 28CAR_11 T cells. This technical limitation likely resulted in dilution of the signal, as the true CAR transduction rate may have been lower for constructs 1 and 11 compared to other constructs with detectable surface expression. Importantly, the reduced IFNγ production in these constructs correlated with diminished cytotoxic activity (as discussed in response to Comment #4), suggesting that IFNγ secretion served as a valid functional readout for CAR-T cell efficacy across all tested constructs. The constructs with higher IFNγ production (28CAR_2, 28CAR_12, and 28CAR_14) consistently demonstrated superior anti-tumor activity (Figure 2C&D).

**Regarding TNFα variability:** TNFα production showed an identical pattern to IFNγ secretion, with high expression levels observed in constructs 28CAR_2, 28CAR_12, and 28CAR_14, further corroborating that these constructs possess superior functional capacity (Supplementary Figure 4A). This concordance between IFNγ and TNFα production reinforces the validity of using cytokine secretion as a functional readout for CAR-T cell quality.

4) Figure 2: panel K,L: the CAR 2 and 12 patterns of relative killing does not correlate with IFNgamma and therefore it is important to see whether other killing mechanisms are at work-also can one block with anti-IFNgamma?

We respectfully disagree with the reviewer's interpretation regarding the correlation between IFNγ production and cytotoxicity.

**Regarding correlation between IFNγ secretion and cytotoxic activity:** Upon careful examination of the data, we observe a clear correlation between IFNγ production and cytotoxic capacity. Constructs 28CAR_2, 28CAR_12, and 28CAR_14 all demonstrated the highest IFNγ secretion in T cells after co-culture with target cells (approximately 20% IFNγ+ cells for all three constructs), and these same constructs exhibited the highest cytotoxicity against both tested cell lines. The slightly reduced killing efficiency of 28CAR_12 compared to 28CAR_2 and 28CAR_14 can be attributed to its diminished proliferative capacity rather than a difference in IFNγ production, as all three constructs show comparable cytokine secretion levels.

**Regarding the mechanism of cytotoxicity and IFNγ blockade experiments:** To directly address the reviewer's important question about whether IFNγ represents the primary cytotoxic mechanism, we performed blocking experiments using an IFNγ-neutralizing antibody and relevant controls. These data are now included in Supplementary Figure 12. Importantly, we observed no significant effect on target cell killing for any of the tested constructs (28CAR_2,

28CAR_12, 28CAR_14, or 7CAR) upon IFNγ blockade. This finding demonstrates that, while IFNγ secretion serves as a robust correlate of CAR-T cell functionality, the actual cytotoxic activity is mediated primarily through direct cytolytic mechanisms (perforin/granzyme-mediated killing) rather than through IFNγ-dependent pathways. This is consistent with previous reports demonstrating that CAR-T cell-mediated tumor cell lysis occurs predominantly via granule exocytosis (PMID: 38307835).

5) Figure 3: Within the same context: this informative figure includes perforin, but to enhance the paper's credibility, it would greatly benefit from FACs analysis showing that the expression levels of perforin and granzyme B are indeed elevated in the more cytolytic CARs. Additionally, it is essential to include data on proliferation, which can be effectively showcased through the expression profiles of Ki67.

We thank the reviewer for the positive feedback on this figure and for the constructive suggestions to strengthen the manuscript.

We fully agree that proliferative capacity is a critical functional parameter for CAR-T cell efficacy. Proliferation data were already included in Figure 2I, showing proliferating T cells as measured by CTV dilution. To further strengthen this analysis, we have now added quantification of co-culture-induced proliferation (Supplementary Figure 4B). This additional analysis clearly demonstrates antigen-specific proliferation selectively in constructs 28CAR_2, 28CAR_12, and 28CAR_14, further corroborating that these constructs exhibit robust antigen-driven expansion. This proliferative response correlates well with their superior cytotoxic activity and cytokine production, providing additional evidence for the functional superiority of these constructs.

6) Figure 4: looks good but would benefit from a characterization of the phenotype of the TILs associated with the CARs.

We thank the reviewer for this excellent suggestion. We agree that characterization of tumor-infiltrating CAR-T cells is essential to understand the mechanisms underlying therapeutic efficacy. To address this point, we performed an additional in vivo experiment specifically designed to assess CAR-T cell phenotype at an early timepoint following tumor encounter. CCRF-CEM leukemia cells ($1\times10^6$) were injected on day -4, followed by CAR-T cell injection on day 0, and mice were sacrificed on day 4 for comprehensive phenotypic analysis.

Our analysis revealed several important findings: CAR-T cell frequency and expansion: CD28-CAR-T cells demonstrated superior expansion compared to CD7-CAR-T cells and achieved frequencies comparable to CD19-CAR-T cells in peripheral blood (Supplementary Figure 10E, previously included) and spleen (Figure 4F, novel).

Analysis of activation-associated markers revealed distinct phenotypic profiles among the different CARs. TIM-3 expression was significantly increased in both CD28-CAR and CD7-CAR-transduced T cells compared to CD19-CAR-T cells (Figure 4H), indicating robust activation upon encounter with their cognate target antigens. Importantly, CD25 (IL-2 receptor alpha chain) mean fluorescence intensity was significantly elevated in CD28-CAR-T cells compared to CD19-CAR-T cells, whereas CD7-CAR-T cells showed no significant increase (Figure 4G). This enhanced CD25 expression suggests superior IL-2 responsiveness and activation capacity specifically in CD28-CAR-T cells. Together, these phenotypic data demonstrate that CD28-CAR-T cells combine robust in vivo expansion, maintenance of a less-differentiated memory phenotype, and strong activation capacity—all characteristics that correlate with their superior therapeutic efficacy.

7) The paper lacks detailed comparative data on the toxicity and cytokine release syndrome (CRS) profiles of anti-CD28 CAR T cells relative to other CAR T-cell therapies.

CD28 expression is largely restricted to hematopoietic cells, including physiologic T cells, so in principle anti-CD28 CAR T cells could mediate on-target off-tumor activation and cytokine release, analogous to CD7-targeted approaches. However, in the setting of T-ALL, CRS risk is primarily driven by leukemic antigen load and overall disease burden at the time of infusion, rather than by expression of T-cell antigens on residual normal lymphocytes. This is supported by clinical experience with CD7-directed CAR T cells in r/r T-ALL/LBL, where patients typically receive fludarabine/cyclophosphamide lymphodepletion that profoundly reduces normal T/NK compartments before CAR T infusion, and CRS is mostly low grade despite high target expression on physiologic T cells. Across several CD7 CAR-T programs, the vast majority of patients developed only grade 1–2 CRS, with grade ≥3 events being rare (PMID: 39227445). In our experimental system, we specifically addressed this concern by co-culturing anti-CD28 CAR T cells with autologous PBMCs and quantifying the impact on non-CAR T-cell and NK-cell subsets. Under these conditions, anti-CD28 CAR T cells induced substantially less bystander lymphodepletion than CD7 CAR T cells and did not measurably reduce Treg frequencies, suggesting a more limited and qualitatively distinct on-target off-tumor effect on the endogenous immune compartment (Figure 5, novel). Together with the published CD7 CAR-T safety data, these findings support the concept that anti-CD28 CAR T cells, when combined with standard lymphodepleting chemotherapy, are unlikely to cause excessive CRS purely due to CD28 expression on physiologic T cells. Nevertheless, given the hematopoietic distribution of CD28, careful clinical monitoring of cytokine profiles and immune reconstitution will be essential in early-phase trials to fully characterize and mitigate on-target off-tumor toxicities

8) Utilizing un-transduced T cells or T cells transduced with a mock CAR (lacking a specific targeting domain) would establish a baseline for understanding the effects of CAR transduction and expansion without the confounding influence of specific antigen targeting.

We agree that appropriate controls are essential to disentangle the effects of transduction/expansion from antigen-specific CAR activity. In all our experiments, we therefore included untransduced T cells (UT) as a reference population, which serve the function of “mock” controls lacking a CAR. Throughout the manuscript, UT are analyzed in parallel to CAR-transduced T cells, for example in Figure 2D, 2F, 2G, 3E and 4B, as well as in novel experiments in Figure 5. These UT controls allow us to establish a baseline for T-cell activation, phenotype and survival after expansion, without the confounding influence of specific antigen targeting, and thereby address the reviewer’s request for non-targeted comparator conditions.

9) Although the study mentions the potential for lower on-target off-tumor toxicity compared to CD7 targeting, more discussion related to this issue is needed to fully understand and mitigate these risks in clinical settings, particularly concerning the preservation of immune system integrity.

Our co-culture data support the notion that CD28-directed CAR T cells have a more limited and qualitatively distinct on-target off-tumor impact on the endogenous immune system than CD7-directed CAR T cells, while still requiring careful clinical monitoring. In autologous PBMC co-cultures where CAR T cells were tracked by CTV and only CTV-negative (non-CAR) cells were analyzed, CD7 CAR T cells caused a profound depletion of both CD4 and CD8 T cells as well as $CD56^+$ cells, consistent with broad CD7 expression on T and NK compartments and indicating substantial global lymphodepletion. In contrast, CD28 CAR T cells also reduced non-CAR T cells but to a markedly lower extent (approximately one order of magnitude less after 48 hours than CD7 CAR T cells) and with a clear bias toward CD4 T-cell loss, suggesting a more restrained and subset-selective fratricide pattern.

Phenotypic analysis of the residual CD4 compartment revealed that CD28 CAR T cells preferentially depleted $CD3^+CD4^+CCR6^+$ cells (including $CCR4^+CCR6^+$ cells) both within $CXCR3^-$ and $CXCR3^+$ subsets, whereas the overall frequency of $CXCR3^+$ CD4 T cells

remained largely preserved relative to non-CAR controls. This points to selective pruning of the Th17/Th17.1/Th2-like $CCR6^+$ helper axis, while Th1-like $CXCR3^+$ cells are comparatively spared, and is in line with the strong functional relevance of CD28 signaling in effector/memory CD4 T cells. Notably, Treg frequencies ($CD4^+$CD25^high^CD127^low^) did not show relevant changes under any of the conditions, arguing against a major loss of regulatory T-cell control as an additional safety concern in this short-term setting.

Both CAR constructs induced target-antigen modulation on bystander T cells, with reduced CD7 MFI on surviving CD4 and CD8 T cells after CD7 CAR exposure and a pronounced decrease of CD28 MFI—more marked in CD4 but also present in CD8 T cells—after CD28 CAR exposure. This likely reflects either preferential survival of antigen-low cells or active down-regulation of the targeted molecules under CAR pressure, and it suggests a dynamic adaptation of the endogenous T-cell pool that may further limit sustained off-tumor killing over time. Taken together, these findings indicate that, compared with CD7 targeting, CD28-directed CAR T cells cause less global lymphodepletion and appear to preserve key components of immune system integrity (including Tregs and a substantial fraction of Th1-like and CD8 T cells), while still reshaping the CD4 helper landscape in a subset-specific manner. Nevertheless, the observed depletion of $CCR6^+$ helper T cells and the induction of CD28 down-modulation underscore that CD28-directed CARs are not immunologically inert; careful clinical monitoring of CD4 counts, helper-subset composition, and infection risk, alongside longer-term in vivo studies, will be essential to fully characterize and mitigate on-target off-tumor effects in patients.

**Reviewer #2 (Remarks to the Author): with expertise in T lineage malignancies, CAR-T**

Dr. Willier and co-authors at LMU identified CD28 as a therapeutically actionable target in T-ALL. They showed CAR T cells targeting CD28 can expand following CD28 editing and produce cytotoxicity against T-ALL cell lines in vitro. In a CCRF-CEM xenograft model, intravenously administered CD28 CART prolong animal survival and delay systemic leukaemia, acting on par with a control CD7 CAR. Most clinically successful CAR-T therapies in T-ALL target CD7 and CD5; CD28 is a new target in this space, which aids in novelty.

While this preclinical report is mostly straightforward, several unaddressed issues limit our understanding of the translational relevance of this work.

1. As the authors mention repeatedly throughout the paper, most normal T cells express CD28. In lines 244-247, the authors state "CD28 as opposed to CD7 is not expressed on all physiologic T cells, which is why T-cell depletion and thereby T-cell immunodeficiency would supposedly be less pronounced after anti-CD28 CAR T-cell therapy". First, this statement must be corrected as CD7 is also not expressed in all T cells, and multiple clinical studies with CD7 CART show systemic enrichment for CD7-negative T cells after infusion. Second, while I truly hope that is true, no data is provided to back the authors' claim about CD28.

We sincerely thank the reviewer for this critical comment, which has prompted us to generate comprehensive experimental data addressing the on-target off-tumor effects of CD28-CAR-T cells on the physiologic immune system, illustrated in novel Figure 5. We also appreciate the opportunity to clarify and correct our initial statement.

We acknowledge that our original statement (lines 244-247) requires correction. The reviewer is absolutely correct that CD7 is also not expressed on all physiologic T cells. Multiple clinical studies have indeed demonstrated systemic enrichment of CD7-negative T cells following CD7-CAR-T cell infusion, confirming that a CD7-negative T-cell subset exists. We have revised this statement in the manuscript to accurately reflect the expression patterns of both antigens and to avoid overstating the contrast between CD28 and CD7.

Comprehensive experimental characterization of on-target off-tumor effects: To directly address the reviewer's concern about the lack of supporting data, we performed extensive co-culture experiments comparing CD28-CAR and CD7-CAR T cells with respect to their impact on autologous physiologic lymphocytes. These new data are now included in the revised manuscript and provide robust experimental evidence for our claim that CD28-directed CAR T cells have a more limited and qualitatively distinct on-target off-tumor impact compared to CD7-directed CAR T cells (Figure 5).

Experimental design: We conducted autologous PBMC co-culture experiments in which CAR T cells were labeled with CellTrace Violet (CTV) to enable tracking. Only CTV-negative (non-CAR) cells were analyzed to assess fratricide effects on bystander lymphocytes. Global lymphodepletion analysis showed, that CD7-CAR T cells caused profound depletion of both CD4+ and CD8+ T cells as well as CD56+ NK cells, consistent with broad CD7 expression across T and NK compartments and indicating substantial global lymphodepletion. In contrast, CD28-CAR T cells also reduced non-CAR T cells but to a markedly lower extent (approximately one order of magnitude less after 48 hours) and with a clear bias toward CD4+ T-cell loss, suggesting a more restrained and subset-selective fratricide pattern. Subset-specific depletion within CD4+ T cells was observed and CD28-CAR T cells preferentially depleted TH2 and TH17 helper T cells. In contrast, the frequency of TH1 helper T cells remained largely preserved relative to non-CAR controls. Importantly, regulatory T-cell frequencies (CD4+CD25highCD127low) showed no relevant changes under any condition, arguing against major loss of regulatory T-cell control as an additional safety concern in this experimental setting.

Both CAR constructs induced target antigen modulation on bystander T cells. CD7-CAR exposure reduced CD7 MFI on surviving CD4+ and CD8+ T cells, while CD28-CAR exposure caused a pronounced decrease in CD28 MFI—more marked in CD4+ but also present in CD8+ T cells. This likely reflects either preferential survival of antigen-low cells or active downregulation of the targeted molecules under CAR pressure, suggesting dynamic adaptation of the endogenous T-cell pool that may further limit sustained off-tumor killing over time.

We have now comprehensively quantified CD28 expression on T-cell subsets from healthy donors (Figure 1B&C). While virtually all CD4+ T cells in healthy young individuals are CD28-positive, approximately 25% of CD8+ T cells are CD28-negative, representing a significant CD28-negative population that would be spared from fratricidal killing. Furthermore, within CD4+ T-cell subsets, CD28 expression varies significantly across differentiation stages: expression increases from naive/stem cell memory (TN/Tscm) to central memory (TCM) T cells before decreasing progressively through effector memory (TEM) to terminally differentiated effector (TEFF) cells (Figure 5I). This expression pattern suggests that CD28-CAR T cells would preferentially target activated/memory helper cells while relatively sparing both naive CD4+ T cells and terminally differentiated effector populations.

Taken together, these findings demonstrate that compared with CD7 targeting, CD28-directed CAR T cells cause significantly less global lymphodepletion and appear to preserve key components of immune system integrity, including regulatory T cells, a substantial fraction of Th1-like CD4+ T cells, and CD28-negative CD8+ T cells. However, the observed depletion of CCR6+ and CCR4+ helper T cells and the induction of CD28 downmodulation indicate that CD28-directed CARs are not immunologically inert.

2. Comparing CD28 expression level, both per cent and MFI, in T-ALL and in normal CD45+ mature T cells in peripheral blood should be included to understand the degree of CD28 overexpression in T-ALL blasts. Supplementary Fig 1D/E shows relative frequency in bone

marrow but it is difficult to understand whether this reflects global picture and whether the expression is brighter on the malignancy.

We thank the reviewer for this important clarification request regarding CD28 expression levels in T-ALL versus normal T cells.

We have now included comprehensive comparative analyses of CD28 expression between T-ALL blasts and healthy peripheral blood T-cell subsets (Figure 1E and 1F). These data reveal that CD28 expression intensity (MFI) and frequency in T-ALL blasts does not surpass that of physiologic CD4+ T cells.

We appreciate the opportunity to clarify our use of the term "overexpression" in the manuscript. When we refer to CD28 overexpression in T-ALL, we are specifically comparing T-ALL blasts to their cell of origin - lymphoid progenitors - not to mature peripheral blood T cells. As shown in our differential expression analyses, CD28 is significantly upregulated in T-ALL blasts compared to normal lymphoid progenitor populations in the bone marrow. This distinction is critical because T-ALL arises from immature T-cell progenitors that normally express little to no CD28 during early development. The aberrant CD28 expression on malignant lymphoblasts therefore represents a developmentally dysregulated expression pattern that distinguishes them from their normal progenitor counterparts and provides a therapeutic opportunity.

The similar expression levels between T-ALL and mature CD4+ T cells explain the observed fratricide effects on CD4+ T cells in our co-culture experiments (Comment #1), while the preservation of CD28-negative CD8+ T cells (~25% in healthy donors) and subset-selective depletion pattern provide a manageable therapeutic window.

We have revised the manuscript to clarify that "overexpression" refers to the comparison with lymphoid progenitors rather than mature T cells.

3. I am confused why the authors compare CD28 levels in T-ALL vs normal CD7+ CD45dim cells in bone marrow? Committed T-cell progenitors reside in the thymus, not in the marrow. The CD7+ BM population could be comprised of mixed lineage (including NK and myeloid) cells and progenitors, only some of which could be heading down the T-cell lineage.

We thank the reviewer for pointing out this important inaccuracy in our description. The reviewer is absolutely correct that committed T-cell progenitors reside in the thymus, not in the bone marrow, and that the CD7+ CD45dim bone marrow population represents a heterogeneous mixture of lymphoid progenitors including NK and myeloid precursors, only some of which may be T-lineage committed.

We have revised the manuscript to accurately refer to the comparison population as "lymphoid progenitors" rather than "T-cell progenitors." The CD7+ CD45dim population in bone marrow indeed comprises mixed-lineage lymphoid progenitors at various stages of commitment. Our comparison demonstrates that CD28 expression is significantly upregulated in T-ALL blasts relative to this early lymphoid progenitor pool.

This comparison remains biologically meaningful because T-ALL can arise from various stages of early lymphoid development, including multipotent progenitors prior to definitive T-lineage commitment. The aberrant CD28 expression in T-ALL blasts—compared to the early lymphoid progenitor compartment from which they derive—provides the therapeutic rationale for CD28 targeting, as it distinguishes the malignant population from the earliest stages of normal lymphopoiesis. We have corrected this terminology throughout the manuscript.

4. Coculture assays of CD28 CART with freshly isolated blood T cells should be performed. The surviving CD28-negative T cell population (if exists) should be characterized for subset

composition to estimate the extent of T-cell aplasia in patients and the quality/functional status of therapy-resistant lymphocytes.

We thank the reviewer for this excellent suggestion. We have performed exactly these experiments and the comprehensive results are presented in Figure 5 and discussed in detail in our response to Reviewer #2, Comment #1.

Briefly, we conducted autologous PBMC co-culture experiments with CTV-labeled CAR T cells and analyzed the surviving CTV-negative (non-CAR) T-cell populations. Our analyses demonstrate that CD28-negative CD8+ T cells are preserved: Approximately 25% of healthy CD8+ T cells are CD28-negative (Figure 1C) and would be expected to survive CD28-CAR-T therapy. Subset-selective depletion: CD28-CAR T cells cause significantly less global lymphodepletion than CD7-CAR T cells (approximately one order of magnitude less after 48 hours) with preferential depletion of CD4+ T cells, particularly CCR6+ and CCR4+ subsets, while largely preserving CXCR3+ Th1-like cells. Preservation of regulatory T cells: Treg frequencies (CD4+CD25highCD127low) remained stable, indicating no major loss of regulatory T-cell control. Antigen modulation: Surviving T cells showed decreased CD28 MFI, suggesting either preferential survival of antigen-low cells or active downregulation under CAR pressure.

These data provide experimental evidence that CD28-CAR T therapy would result in less severe T-cell aplasia than CD7-CAR T therapy while preserving functionally important T-cell subsets including CD28-negative CD8+ T cells, Th1 cells, and Tregs.

5. Potential additional ablation of plasma B cells is a concern. Do the authors also expect total reset of that population - or this therapy is designed mainly as a bridge to alloHSCT? Discussing these aspects would help the reader understand whether CD28 CART will be safer than CD7 targeting in T-ALL.

We thank the reviewer for this highly relevant question. Despite attempts to test for plasma cell depletion in a in vitro assay, we were not able to obtain material containing sufficient plasma cells for robust monitoring. We have included, however, this issue in the discussion and discuss the potential loss of long lived, humoral immune memory bearing plasma cells by anti-CD28 CAR T cells.

6. CD28 modulation on recurrent CCRF in vivo is interesting. Adding a reference control would help understand if the downregulation is partial or complete; flow plots would be also helpful. Does this reflect a selection of a rare CD28dim/negative CCRF clone, or a genuine CD28 downregulation on leukaemia? Testing a couple of additional T-ALL xenografts, ideally patient-derived, can help understand how common this mechanism is, and whether it constitutes a general hurdle in therapeutic CD28 targeting.

We thank the reviewer for this important suggestion to clarify the nature and generalizability of CD28 modulation in vivo. In response, we repeated the CCRF-CEM in vivo experiment and confirmed that CD28 expression is indeed downregulated on bone marrow-infiltrating CCRF-CEM cells after CD28-CAR-T therapy; representative flow cytometry plots directly comparing treated versus untreated controls have been added to the Supplementary Materials, allowing visualization of the extent (partial rather than complete) of CD28 loss.

To address whether this represents selection of a rare CD28^dim/negative subclone or a broader, generalizable resistance mechanism, we performed analogous in vivo experiments in two additional T-ALL models, the Jurkat cell line and the patient-derived xenograft PDX-ALL230. In both models, CD28 expression remained stable at relapse, with no evidence of CD28 downregulation, with baseline CD28 expression being highest in CCRF-CEM cells

than in Jurkat and lower but still robust baseline expression in PDX-ALL230. CD28 expression at relapse for all three models is now shown in Figure 4k and Supplementary Figure 11.

Importantly, CD28-CAR-T cells provided effective tumor control and prolonged survival in mice bearing either Jurkat or PDX-ALL230 leukemia, as demonstrated by in vivo bioluminescence imaging and survival curves (Figure 4H and Supplementary Figure 6), indicating that therapeutic efficacy is preserved in settings where CD28 remains stable under selective pressure. Together, these data suggest that CD28 downregulation is not a common or universal resistance mechanism in T-ALL but rather a CCRF-CEM-specific phenomenon. The absence of CD28 loss at relapse in both Jurkat and the clinically more relevant PDX-ALL230 model supports the conclusion that CD28 is generally a robust and stable therapeutic target in T-ALL, and that CCRF-CEM-specific modulation likely reflects unique biology of this particular cell line rather than a broadly applicable hurdle for CD28-directed therapy.

7. The Results section pertaining to Figure 2 is all over the place. It starts with 2B, then 2G-I, then 2A and so on. I could not find a description of the experiment in Figure 2F. Figure 3A is also not referenced. 4D comes before 4C. Can the authors describe the figure in the order the panels are shown?

We thank the reviewer for his helpful comment: it is true, that chronology of figure was illogical. We have adjusted this and hope, reading of the manuscript will get smoother this way.

8. The authors' results suggest CD28 CARs mask their own CD28 which could aid in fratricide evasion. Assuming the CD28 KO efficiency in cGMP is not 100%, this leaves the possibility of "contaminating" CD28wt CD28 CART cells in the final product. Would these unedited CD28 CART be constantly revved up upon continuous cis-engagement of the CD28 superagonist binder and possibly cause a cytokine storm? If so, discussing potential mitigation strategies will help the reader.

We thank the reviewer for raising this important safety concern regarding potential cis-engagement of the TGN1412-based CD28 scFv and the possibility of inadvertent T-cell hyperactivation or cytokine storm from unedited CD28wt CAR-T cells in the final product.

An important advantage of CD28-directed CAR-T therapy over CD7-directed approaches is that CAR-T cell product generation is feasible without prior CD28 knockout. This contrasts sharply with CD7-CAR-T cell production, where T-cell counts are reduced by >90% without performance of CD7 knockout prior to CAR transduction due to severe fratricide. The ability to manufacture CD28-CAR-T cells without obligatory gene editing simplifies the production process and reduces technical complexity.

We have systematically evaluated the potential risk of T-cell hyperactivation through TGN1412 scFv-mediated CD28 engagement and found no evidence of aberrant activation: No bystander T-cell activation: When CD28+ non-CAR T cells were placed in co-culture with TGN1412-based CD28-CAR-T cells (28CAR_2), we observed no activation of the CD28+ bystander T cells (Supplementary Figure 5A-E). This demonstrates that the TGN1412 scFv does not act as a superagonist in trans to activate neighboring CD28+ T cells.

Normal cellular composition and differentiation: Analysis of 14-day expanded CAR-T cell products revealed that cellular composition and T-cell maturation profiles were not different between TGN1412-based (28CAR_2) and CD28.3-based (28CAR_14) CAR constructs (Supplementary Figure 5A, B). This indicates that incorporation of the TGN1412 scFv does not skew T-cell differentiation or alter product composition during manufacturing.

Assessment of baseline phenotype status in 14-day expanded CAR-T cells showed that 28CAR_2 (TGN1412-based) T cells did not exhibit increased activation compared to 28CAR_14 (CD28.3-based) or 7CAR-T cells (Figure 3I). This directly addresses the concern

about constitutive cis-engagement and demonstrates that the TGN1412 scFv does not drive tonic signaling or spontaneous activation.

Our comprehensive experimental data demonstrate that the "revving up" scenario described by the reviewer does not occur. The TGN1412-based CD28 scFv, when incorporated into a CAR construct, does not cause constitutive T-cell activation, does not activate bystander CD28+ cells, and does not compromise the safety profile of the CAR-T cell product. These findings support the clinical feasibility of CD28-CAR-T therapy and suggest that the presence of residual unedited CD28wt cells in the product would not pose an elevated risk of cytokine storm or aberrant T-cell activation.

9. The pattern of CD28 vs 2B4 expression is intriguing but I would not call it a meaningful correlation as only 1/3 (35%) of all data are explained by this regression.

We thank the reviewer for this accurate statistical observation. The reviewer is correct that with an $R^2$ value of approximately 0.35, only one-third of the variance in the data is explained by this regression, which does not constitute a strong correlation by conventional statistical standards.

We have revised the manuscript to more accurately reflect this finding. Rather than describing this as a "meaningful correlation," we now present it as an "intriguing pattern" or "trend" between CD28 and 2B4 expression that warrants further investigation. While the association is statistically significant, we acknowledge that the limited explanatory power suggests that CD28 and 2B4 expression are influenced by additional independent factors.

10. While the brief in vitro data on CD28 targeting in MM cell lines is of interest , stating in the abstract that CD28 is a novel immunotherapy target in MM warrants a deeper investigation.

We agree with the reviewer's assessment. The multiple myeloma (MM) data presented in our study are limited to preliminary in vitro experiments with cell lines and do not constitute a comprehensive preclinical evaluation sufficient to substantiate a claim of CD28 as a novel immunotherapy target in MM.

We have revised the abstract and manuscript to more accurately reflect the scope of our MM findings. The abstract now focuses on T-ALL as the primary indication for CD28-directed CAR-T therapy, with the MM data appropriately framed as preliminary observations suggesting CD28 as a potential therapeutic target that warrants further investigation. We have toned down claims regarding MM and clearly positioned these findings as exploratory data that require deeper preclinical validation, including in vivo studies and analysis of primary patient samples, before any definitive conclusions can be drawn about clinical applicability in MM.

Minor comment

11. To my knowledge, the 3A1f clone of CD7 mAb used for a control CD7 CAR in this study has not been validated clinically due to better-performing binders. I could be wrong.

We thank the reviewer for pointing this out. Indeed, several CD7-binding scFvs have been described, including those derived from the 3A1e, 3A1f and TH-69 antibody clones. In the seminal work by Gomes-Silva et al., these binders were compared head-to-head and 3A1e was selected for further preclinical development based on superior performance. Subsequent reviews of CD7 CAR-T cell therapy also primarily highlight TH-69 and 3A1e as the scFvs that have been carried forward into clinical trials.

In our study, we deliberately used the 3A1f-based CD7 CAR as a well-defined, experimentally tractable reference, rather than to model a specific clinical CD7 CAR product. We now clarify this point in the Methods/Discussion and explicitly state that 3A1f was chosen as a preclinical

benchmark binder, whereas most current clinical CD7 CAR programmes use alternative scFvs (such as 3A1e or TH-69).

However, in our hands, when trying to generate functional CAR molecules with published CD7 binders, CD7 CAR based on 3A1f was the best-perforrming CART which triggered us to use it as a reference in our experiments.

**Reviewer #3 (Remarks to the Author): with expertise in T lineage malignancies, CAR-T**

The authors detail the expression of CD28 on T-ALL in pediatrics and evaluate it as a novel target of CAR T cells. They do a thorough evaluation of CD28-CAR T cell utility with benchmark comparison to CD7. Novel aspects of this study include the generation of CD28ko cells which appear to retain functionality, although do have a loss of cytotoxicity in vitro and development and evaluation of CD28 specific CAR T cells which could potentially be taken to clinical trials.

1. Intro – there isn't any mention of previous work looking at CD28 expression in pre-T malignancies. This should be mentions. Consider PMCID 8748252

We thank the reviewer for highlighting prior work on CD28 expression and function in T-cell and pre-T malignancies. Our original Introduction did not sufficiently acknowledge these data. Several studies have demonstrated that CD28 can be aberrantly expressed and genetically altered in mature T-cell neoplasms, supporting its role as a disease driver and potential therapeutic target. For example, in adult T-cell leukemia/lymphoma (ATLL), CD28 overexpression is observed in approximately 55% of cases and correlates with CD28 gene fusions, copy-number variations and somatic mutations, and is associated with an unfavorable prognosis (PMID: 34738707 / PMCID: PMC8748252; PMID: 31961925; PMID: 33128207). Recurrent activating CD28 point mutations and fusion events (e.g. CTLA4–CD28, ICOS–CD28) have been described in peripheral T-cell lymphomas and ATLL, where they enhance ligand binding and downstream signaling, thereby promoting T-cell activation and growth (PMID: 28711152; PMID: 26459350; PMID: 26518022). In addition, CD28 expression has been reported on Epstein–Barr virus–positive B-cell lines and AIDS-associated non-Hodgkin lymphoma cell lines, where CD28 ligation induces CD25 and CD71 upregulation and can protect from Fas-induced apoptosis, further illustrating the oncogenic potential of aberrant CD28 signaling beyond normal T cells (PMID: 12853703).

We now cite these studies (<u>line 98</u>) in the revised Introduction and explicitly position our work as extending this concept to pediatric T-ALL by providing a systematic analysis of CD28 surface expression on pediatric T-ALL samples and by functionally evaluating CD28 knockout and CD28-directed CAR T cells in this disease context.

2. Line 94- Is there something missing after CD7? "than both CD7"

We thank the reviewer for noticing this typo. The sentence was indeed incomplete. We have now corrected it to: "CD28 expression occurs later in T-cell development than both CD7 and CD2." This statement is supported by our flow cytometric analysis of normal thymic subsets shown in Supplementary Figure 2 H–K.

3. For the experiment of CD28 ligation by CD86 – is it possible to culture with monocytes that doesn't have CD86 co-expression to compare? As the CD28 ko cell did not observe any proliferation change, was this done as well with the monocytes expressing CD86? My read is that the beginning is looking to see that ligation of CD28 and CD86 confers a proliferation signal, and this seems to be accounted for, at least in part, though IL-7. Did you look in the supernatant of the DC+CCRF-CEM to determine IL-7 levels, and in comparison to the CCRF-CEM culture and DC culture alone? Meaning can you confirm higher levels of IL-7 and also that it is a direct result of engagement of the DC+CCRF-CEM. I don't believe that this

evaluation is of utmost importance for the value of this paper, but I do think the statement of IL-7 accounting for the effect needs to be shown more thoroughly if it is going to be stated.

We thank the reviewer for these important mechanistic questions. In response, we performed additional experiments that led us to revise our interpretation of the CD28-CD86 co-culture data:

Regarding CD86-dependent signaling in moDC co-cultures: To test whether the proliferative advantage conferred by moDCs depends on specific CD28-CD80/CD86 engagement, we employed the CD28-blocking fusion protein Abatacept (CTLA4-Fc) in our co-culture system. Surprisingly, Abatacept treatment did not significantly reduce T-ALL proliferation in the presence of moDCs (new SF 6C), suggesting that direct CD28-CD80/CD86 interaction is not the primary mechanism driving this effect.

We validated the sensitivity of this approach using a positive control system: primary T cells activated by blinatumomab in the presence of CD80/CD86-overexpressing Nalm6 BCP-ALL cells showed robust proliferation that was dose-dependently suppressed by Abatacept (10-200 µg/ml; new SF 6E), confirming that our experimental system is capable of detecting CD28-dependent effects when present.

Regarding IL-7 measurements: We agree that direct cytokine quantification would strengthen the mechanistic interpretation. We have not yet performed IL-7 ELISA on co-culture supernatants. Given the negative Abatacept result, we have revised our text to state that IL-7 contributes to T-ALL proliferation under these conditions rather than implying it is a direct consequence of CD28-CD86 engagement. We now interpret the moDC co-culture effect as IL-7-dependent but CD28-CD80/CD86-independent.

To further probe CD28 functionality on T-ALL, we subjected Jurkat and CCRF-CEM cell lines to a panel of targeted inhibitors and chemotherapeutics (γ-secretase inhibitor DBZ, JAK1/2 inhibitor Ruxolitinib, MEK inhibitors Trametinib/Cobimetinib, PI3K inhibitors Idelalisib/Buparlisib/Gedatolisib, cytarabine, and dexamethasone) while simultaneously providing CD28 costimulation via conventional or superagonistic antibodies.

We observed that CD28 stimulation conferred a growth advantage specifically and only under JAK inhibition (new SF 6G). No proliferative effect of CD28 ligation was detected in standard culture conditions, serum-reduced media, or during inhibition of NOTCH, MEK, or PI3K/mTOR pathways. This suggests that pharmacologic JAK blockade unmasks a latent, JAK-independent pro-survival program downstream of CD28, likely mediated through the constitutively active PI3K/AKT pathway present in both cell lines (PTEN loss in Jurkat; upstream PI3K/AKT lesions in CCRF-CEM).

We have revised the relevant sections to reflect these findings, toning down claims about CD28-CD86-mediated proliferation and clarifying that: (1) IL-7 contributes to T-ALL growth but is not necessarily induced by CD28-CD86 engagement; and (2) CD28 functionality on T-ALL cells is context-dependent and becomes relevant primarily under conditions where JAK/STAT signaling is suppressed (Supplementary Figure 3A-H).

4. Figure 1B – from the gating – it is showing the expression of CD28 on the CD7+ gate or of the sCD3 gate – It seems like the later but the way it is presented is confusing and seems like it is only showing CD28 expression on the CD7 gate. If it is of just the CD7 gate, it would seem helpful to also show the CD28 expression on all T cells.

We apologize for the confusion in the presentation of Figure 1B. To clarify: Current gating strategy: Figure 1B shows CD28 expression (MFI) on both T-ALL blasts (CD7+ gate) and healthy T cell populations (sCD3+ gate, further subdivided into CD4+ and CD8+ subsets). We have now revised the figure legend and panel labeling to make this gating strategy explicit.

As the reviewer correctly anticipated, analyzing CD28 expression across all T cell populations reveals important context: CD28-expressing T-ALL cases display MFI levels comparable to healthy CD4+ T cells, whereas CD28-negative T-ALL cases show markedly reduced expression—lower than both CD4+ and CD8+ T cells. This comparison to normal T cell subsets strengthens the biological interpretation of CD28 heterogeneity in T-ALL.

Additional context from Figure 5I: To further illustrate physiological CD28 expression patterns, Figure 5F demonstrates CD28 variation across T cell differentiation states: CD28 is highest on naïve/stem cell memory T cells (CD62L+CD45RO−), intermediate on central memory cells (CD62L+CD45RO+), and progressively lower on effector memory (CD62L−CD45RO+) and terminal effector cells (CD62L−CD45RO−). This pattern is observed in both CD4+ and CD8+ compartments, with generally lower levels in CD8+ T cells—consistent with established T cell biology and providing a reference framework for interpreting CD28 expression on T-ALL blasts.

5. Figure 1C – what does it look like in comparison to CD7? In looking at supplemental figure, its presenting the expression on all bone marrow T cells and it looks like a substantial amount of both CD7 and CD28 is present. Why make the distinction of the expression of C28 on BMT cells v progenitor T cells?

We thank the reviewer for this question, which allows us to clarify an important distinction.

CD7 as a pan-T-cell marker: CD7 expression is nearly universal in T-ALL, with >95% of cases expressing CD7 (PMID: 28330870, 33811356). Similarly, CD7 is expressed throughout normal T cell development: it is present on early lymphoid progenitors (used as a gating marker in our analysis), thymic T cell progenitors, and mature peripheral T cells (new Supplementary Figure 4F&G). In our dataset, both CD4+ and CD8+ T cells express high levels of CD7, with comparable MFI to T-ALL blasts (CD8+ T cells showed somewhat lower expression than CD4+ T cells).

CD28 shows developmental stage-specific expression: In contrast to the ubiquitous CD7 expression, CD28 displays marked heterogeneity both in T-ALL (~60% of cases; Figure 1A-B) and across normal T cell developmental stages (Figure 1C). Critically, CD28 is absent or low on early T cell progenitors but increases during T cell maturation—a pattern distinct from CD7.

Rationale for focusing on progenitor populations: The distinction we are making is between T-ALL blasts (malignant) and physiologic T cell progenitors (normal developing thymocytes), not between lymphoid progenitors and mature bone marrow T cells. This comparison is essential for predicting T cell reconstitution kinetics after CAR T therapy. CD7-targeted CAR T cells would deplete cells across all developmental stages (progenitors through mature T cells) due to pan-stage CD7 expression, potentially causing prolonged T cell aplasia. CD28-targeted CAR T cells would spare CD28-negative early progenitors, theoretically allowing faster emergence of CD28-negative T cells from ongoing thymopoiesis.

This hypothesis is supported by clinical observations from CD7-CAR trials, which have documented emergence of CD7-negative T cell escape variants months after treatment (PMID: 28330870, 38355631), suggesting selective pressure spares or generates antigen-negative cells. The later developmental acquisition of CD28 compared to CD7 provides a mechanistic basis for anticipating shorter T cell aplasia with CD28-directed versus CD7-directed CAR therapy.

We have added Supplementary Figure 1C and 13B to provide CD7 expression as a reference point, demonstrating that unlike CD28, CD7 is uniformly expressed across T cell populations.

6. Figure 1D – is it possible to include CD7 and CD5 in this analysis?

We thank the reviewer for this suggestion. CD5 expression: Unfortunately, CD5 was not included in the flow cytometry panel used for Figure 1D, and we therefore cannot provide a direct comparison of CD5 expression relative to CD28 in this cohort. We acknowledge that CD5 represents another clinically relevant T-lineage marker and potential CAR target. CD7 expression: CD7 was expressed on all T-ALL samples analyzed in this study. We must note, however, that CD7 was used as a gating marker to identify T-ALL blasts in our flow cytometry analysis, which creates a selection bias: samples were pre-gated on CD7+ events, precluding accurate assessment of CD7-negative T-ALL cases that may exist in the broader population.

Previous large cohort studies have established that CD7 is expressed in >95% of pediatric T-ALL cases (PMID: 28330870, 33811356), whereas our data demonstrate that CD28 expression is present in approximately 60% of cases (Figure 1A-B). We therefore acknowledge that CD7 expression is substantially more frequent than CD28 in pediatric T-ALL, making CD7 a more broadly applicable target antigen. However, this ubiquity comes with the trade-off of deeper and more prolonged T cell depletion, as discussed in our response to the previous comment regarding developmental expression patterns.

Implication for target selection: While CD28's lower expression frequency (~60%) limits its applicability as a single-agent CAR target compared to CD7 (>95%), it may offer advantages in terms of: Reduced on-target off-tumor toxicity (sparing CD28-negative progenitors), Shorter T cell aplasia duration, Potential utility in combination or sequential strategies (e.g., CD7-CAR followed by CD28-CAR for residual disease),

7. Is there a decrease in cytotoxicity when signaling through a CAR in T cells with CD28 k/o? This is critically important to show that the CAR T cells will have high functionality in vivo. Did you do CD28k/o in a CD7 CAR T cells and compare its functionality? I can't follow figure 3 CDEFGH to understand which cells in the fold expansion and target killing had D28 and CD7 k/o. Perhaps this is all there already and just needs to be made more explicit.

We thank the reviewer for this critical question and apologize for any ambiguity in our figure presentation. Does CD28 KO impair CAR T cell cytotoxicity? This was indeed one of our primary concerns when designing this project. While we did not specifically test CD28 KO in CD7-CAR T cells, we performed several experiments that collectively address whether CD28 deletion compromises T cell effector function: Primary evidence - TCR-redirected killing as a CAR surrogate (Figure 2J): We generated CD28 KO and wild-type (WT) primary T cells, expanded them for 14 days, then assessed cytotoxic capacity using Nalm6 target cells in the presence of blinatumomab. This bispecific T cell engager redirects T cells to kill CD19+ targets similarly to CAR-mediated cytotoxicity—both involve TCR/CD3 signal transduction leading to target recognition and lysis. Results: At 1:1 E:T ratio: No difference in killing between CD28 KO and WT T cells (Figure 2J, left panel) With CD86-overexpressing Nalm6: No significant difference in cytotoxicity. With CD80-overexpressing Nalm6 only: CD28 KO T cells showed modestly reduced killing. Interpretation: CD28 KO T cells retain full cytotoxic capacity under standard conditions and even against CD86-expressing targets (the physiologic, low-affinity CD28 ligand). Only supraphysiologic CD80 overexpression caused modest impairment.

8. I do not understand the relevance of the TGN1412 experiment (lines 157-162). Perhaps there is a different way to stress the point made in this experiment. I presume this links to the importance of lines 184-186 but I am not following the T cell independent effect on ALL.

We aimed to determine whether the TGN1412 monoclonal antibody exerts any intrinsic anti- or pro-proliferative effects on T-ALL cells, given that this antibody has previously been reported to strongly activate and promote proliferation of human $CD28^+$ $CD4^+$ T cells, resulting in severe cytokine release syndrome during a clinical trial in healthy volunteers (PMID: 16908486). In our study, we demonstrate that (i) wild-type autologous T cells are not activated by the superagonistic TGN1412 scFv incorporated into 28CAR_2 T cells (Supplementary Figure 5), and (ii) the soluble TGN1412 monoclonal antibody moderately reduces proliferation of CCRF-CEM cells, whereas a non-superagonistic CD28.3 antibody has no effect (Supplementary Figure 8A,B).

9. Discussion: if loss of CD28 leads to decreased cytotoxicity of T cells, what are the implications of CD28k/o T cells – this should at least be discussed. Potentially when discussing about CD7 CAR T cells and potential advantages of using CD28 CAR T cells.

We thank the reviewer for his comment. We would argue, that we demonstrate in our manuscript that CD28 KO does not impair cytotoxicity (as elaborated in the second last response to Reviewer 3).

**Response to Reviewer #1**

"1) … however, it is a shame that the paper is missing clear data on mechanism. The showed the correlation between interferon gamma and killing but to their credit they found that the addition of blocking antibodies did not affect killing. Unfortunately, the paper does not appear to show a TNF-α blocking assay. Since TNF-α can directly induce apoptosis in some tumor cells--did they try this straightforward experiment? If not, the clearly perforin-GZM is most likely--they show some perforin correlation data however this evidence in support of this pathway would be clearer with some data on granzyme A or B expression (if possible) or the blocking of cell-cell contact with anti-LFA-1 needed for perforin-GZM killing or some data showing in vitro killing of targets with the CARs... This may not be obligatory for acceptance but it would greatly improve the paper and the importance attached to the paper--something that demonstrates how CAR CD28 is operating mechanistically—"

We are grateful for the reviewer's positive overall assessment and for the detailed mechanistic suggestions.

**1) Mechanism of CAR T cell–mediated killing (TNF-α, perforin/granzyme, cell–cell contact)**

The reviewer noted that our initial submission lacked direct evidence to distinguish between cytokine-mediated killing and perforin–granzyme–dependent cytotoxicity, and suggested TNF-α blocking experiments as well as additional data on granzyme expression and cell–cell contact–dependent killing.

In response, we have performed the following additional experiments:

**TNF-α blockade:** We carried out co-culture experiments with tumor target cells in the presence of a TNF-α–blocking antibody and assessed CAR T cell–mediated cytotoxicity. In line with our prior IFN-γ blocking data, TNF-α inhibition did not reduce tumor cell killing, indicating that direct TNF-α–mediated apoptosis is not the dominant mechanism of cytotoxicity in our system. These new data are described in the Results section (page 6, lines 177 ff) and presented in Supplementary Figure 12B.

**Granzyme B expression:** To directly assess involvement of the perforin–granzyme axis, we quantified granzyme B expression in anti-CD28 CAR T cells. We observed that anti-CD28 CAR T cells express granzyme B at high levels, exceeding those of CD19 CAR T cells. In addition, to confirm active release of cytotoxic effector molecules during target cell engagement, we performed co-culture experiments of CAR T cells with the T-ALL cell line CCRF-CEM and analyzed the supernatants using a Legendplex assay (Biolegend). In these experiments, we observed markedly increased granzyme B levels of up to 100 ng/ml and, to a lesser extent, granzyme A upregulation, together with concomitant perforin upregulation of up to 500 pg/ml in the co-culture supernatant. These findings further support active engagement of the perforin–granzyme cytotoxic axis in anti-CD28 CAR T cells. This strong granzyme expression and secretion pattern supports the notion that perforin–granzyme–mediated cytotoxicity is a major mechanism underlying the killing observed with anti-CD28 CAR T cells. These data are now included in the Results (page 6, from line 178) and are shown in detail in Supplementary Figure 16A-E.

**LFA-1 blockade and contact dependency:** Following the reviewer's suggestion to investigate cell–cell contact–dependent killing, we performed co-cultures in the presence of blocking antibodies against LFA-1. In our system, LFA-1 blockade did not result in a consistent substantial reduction of CAR T cell–mediated killing. Mechanistically, cytotoxic granule–mediated killing can remain effective even when LFA-1–mediated adhesion is impaired, because CAR T cells can rely on alternative adhesive molecules and synapse-stabilizing

interactions (such as CD2–LFA-3 or other integrins) to form a functional immunological synapse. Moreover, CAR signaling can be sufficiently strong to allow serial, short-lived contacts that still permit efficient granule delivery, particularly under the relatively high effector-to-target cell ratios used in our in vitro assays. Thus, the lack of a marked reduction in cytotoxicity upon LFA-1 blockade is consistent with a model in which perforin–granzyme–dependent killing remains the predominant mechanism, but is not dependent on LFA-1 (we observed the same for anti-CD7 CAR T cells in this assay). This data is discussed in but not included in the revised manuscript (line 183ff).

Taken together, the negative IFN-γ and TNF-α blocking results, combined with high granzyme B expression in anti-CD28 CAR T cells and the release of granzyme A, granzyme B, and perforin into the co-culture supernatant, support the conclusion that the perforin–granzyme pathway is the principal mechanism of tumor cell killing in our model, rather than direct cytokine-induced apoptosis.

**2) Expansion of the CD28 introduction and signaling background**

"2) the introduction on CD28 needs a major improvement with more information on CD28, how it signals via PI 3K, GRB2, Lck-PKCtheta, mRNA stability, issues related to previous clinical work with autoimmunity and its role in different contexts with the corresponding references (June, Thompson, Rudd, Altman etc.) --this is important—"

We appreciate the reviewer's request for a more comprehensive introduction on CD28 biology, signaling, and prior clinical experience. In response, we have substantially expanded the Introduction (lines 67–76) to better contextualize CD28 costimulation in T cell activation and CAR T cell function, explicitly integrating the key studies suggested by the reviewer.

Specifically, we now:

- More clearly describe CD28 as a co-stimulatory receptor that synergizes with the TCR to enhance lymphokine production, promote T cell activation and survival, and reduce susceptibility to immunosuppressive signals, based on the foundational work by June and colleagues on the role of CD28 in T-cell activation (reference 10: June et al., *Immunology Today* 1990).
- Summarize CD28 signaling via PI3K and Akt and its impact on metabolic reprogramming, emphasizing that CD28 engagement regulates glucose metabolism to support sustained T cell proliferation and effector function, as demonstrated by Frauwirth et al. (reference 11: Frauwirth et al., *Immunity* 2002).
- Highlight that, subsequent to CD28 engagement, PI3K-independent signaling via VAV and SLP-76 can be triggered, linking CD28 to adaptor complexes and cytoskeletal remodeling during T cell activation, in line with the model proposed by Rudd and Raab (reference 12: Rudd & Raab, *Immunological Reviews* 2003).
- Describe how efficient activation of NF-κB, NFAT, and AP-1 requires integration of TCR and CD28 signals, with PKCθ acting as a key effector in CD28-dependent transcriptional control of IL-2 and other cytokines, as reviewed by Isakov and Altman (reference 13: Isakov & Altman, *Annual Review of Immunology* 2002).
- Point out that CD28 costimulation can further increase IL-2 production by stabilizing IL-2 mRNA, drawing on the work by Ragheb et al. showing that CD28-mediated regulation of mRNA stability depends on sequences within the IL-2 coding region (reference 14: Ragheb et al., *Journal of Immunology* 1999).

Together, these additions more explicitly anchor our Introduction in the established CD28 signaling literature and clarify how CD28-dependent pathways can mechanistically contribute to the enhanced effector function of our anti-CD28 CAR T cells.

At the same time, the previous version already mentions the surprising results of the first-in-man phase 1 clinical trial of TGN1412 in six healthy volunteers at a contract research organization and do cite the relevant NEJM publication on this issue. This is of particular interest as one of our most functional anti-CD28 CAR constructs does use the TGN1412 scFv.

**Response to Reviewer #2**

We thank the reviewer for the positive evaluation and the statement that the previous comments have been adequately addressed. No additional changes were requested.

**Response to Reviewer #3**

We are grateful to the reviewer for the careful review and for confirming that the prior concerns have been appropriately addressed. No further modifications were requested.

Rebuttal to Reviewer #1, Comment 2

Reviewer #1, point 2:

"The assertion that anti-LFA-1 blockade has no effect on cytotoxicity is highly unexpected... The need to include the data, even as a supplementary figure, and include proper positive controls demonstrating that the concentration of the anti-LFA-1 blocking antibody used are biologically active and sufficient to successfully inhibit LFA-1 binding/adhesion in their experimental setup. Without this control, it is not really possible to determine whether LFA-1 is truly dispensable or if the blockade was simply ineffective."

Response:

We thank the reviewer for raising this important point. These co-culture experiments had already been performed prior to submission of the revised manuscript in June 2026. Because the observed effects on cytotoxicity were modest, we were previously hesitant to include the full dataset and only referenced it briefly in the text. In light of the reviewer's comment, we have now included the requested data in a new Supplementary Figure 17 and revised the corresponding Results text (lines 187–199, track-changes version attached) as follows:

"As cytotoxic lymphocyte killing relies on the LFA-1/ICAM-1/2 adhesion complex, we asked whether LFA-1 has a role in anti-CD28 CAR T cell killing. To that end, we performed co-cultures of primary human CAR T cells with repetitive addition of GFP-labelled Jurkat cells in the presence or absence of an anti-LFA-1 blocking antibody (Supplementary Fig. 17). In these assays, LFA-1 blockade consistently increased the fraction of surviving Jurkat cells in two independent donors for anti-CD28 CAR T cells using 28CAR_2 and 28CAR_14 and for anti-CD7 CAR T cells. While killing after first T-ALL challenge was attenuated in one donor, only third T-ALL elimination was reduced in the other. No detectable difference between antibody conditions were observed for poorer-performing 28CAR_12. Together, these data indicate that LFA-1 blockade has a modest impact on repetitive T-ALL killing by anti-CD28 CAR T cells, while overall cytotoxic capacity is maintained. This is in line with recent findings that LFA-1 primarily enhances cytotoxic lymphocyte synapse stability and cytotoxic efficiency rather than acting as an absolute requirement for target cell killing."

Regarding the reviewer's request for a positive control confirming that the anti-LFA-1 antibody concentration used is biologically active: the same clone (R7-1, BE0192, Bio X Cell) at the same concentration (10 µg/ml) that we used has been independently shown in a peer-reviewed functional adhesion assay to reduce leukocyte (monocyte and neutrophil) adherence to approximately 20–30% of isotype control levels, a statistically significant effect (PMID: 23179077, Stark et al., Nat Immunol 2013, Supplementary Fig. 1c). This confirms that this

reagent and dose are sufficient to effectively block LFA-1-mediated adhesion. This external validation is further supported by our own data: the reproducible, statistically significant reduction in cytotoxicity upon LFA-1 blockade across three independent CAR T cell constructs (28CAR_2, 28CAR_14, 7CAR; Supplementary Fig. 17B, C, E) — paired with the absence of any effect in the poorly-performing 28CAR_12 (Supplementary Fig. 17D) — would not be expected from an inactive reagent, and instead indicates that the antibody is functionally active in our assay while LFA-1 makes only a modest contribution to overall killing capacity.

We hope that the inclusion of these data, the revised wording, and the independent functional validation of the blocking antibody address the reviewer's concern.
